# High-Throughput Microarray Approaches for Predicting the Stability of Drug–Polymer Solid Dispersions

**DOI:** 10.1021/acs.molpharmaceut.4c00955

**Published:** 2024-12-21

**Authors:** Noha F. Ghazi, Jonathan C. Burley, Ian L. Dryden, Clive J. Roberts

**Affiliations:** † School of Pharmacy, University of Nottingham, University Park, Nottingham NG7 2RD, United Kingdom; ‡ Department of Statistics, University of South Carolina, Columbia, South Carolina 29208, United States; § School of Life Sciences, University of Nottingham, University Park, Nottingham NG7 2UH, United Kingdom; ∥ Department of Pharmaceutics, Faculty of Pharmacy, Mansoura University, Mansoura 35516, Egypt

**Keywords:** high-throughput microarray, miniaturization, inkjet printing, solid dispersion, polarizing microscopy

## Abstract

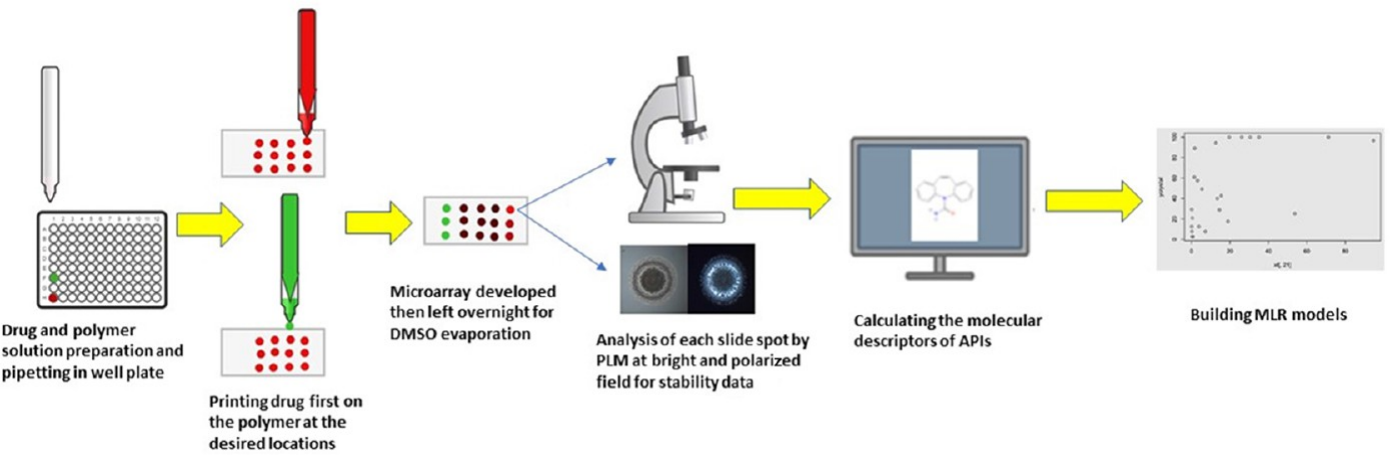

Amorphous solid dispersions (ASDs) offer a well-recognized strategy to improve the effective solubility and, hence, bioavailability of poorly soluble drugs. In this study, we developed an extensive library of a significant number of solid dispersion formulations using a library of chemically diverse drugs combined with a water-soluble polymer (polyvinylpyrrolidone vinyl acetate, PVPVA) at different loadings. These formulations were printed as microarrays of solid dispersion formulations, utilizing minimal material amounts (nanograms). They were subjected to a six-month stability study under accelerated conditions (40 °C and 75% relative humidity). Physical stability outcomes varied significantly among the different drug–polymer combinations, with stability ranging from immediate drug crystallization to several days of stability. The comprehensive data set obtained from this high-throughput screening was used to construct multiple linear regression models to correlate the stability of ASDs with the physicochemical properties of the used Active Pharmaceutical Ingredients (APIs). Our findings reveal that increased stability of ASDs is associated with a lower number of hydrogen bond acceptors alongside a higher overall count of heteroatoms and oxygen atoms in the drug molecules. This suggests that, while heteroatoms and oxygen are abundant, their role as hydrogen bond acceptors is limited due to their specific chemical environments, contributing to overall stability. Additionally, drugs with lower melting points formed more stable ASDs within the polymer matrix. This study, hence, highlights the importance of minimizing repulsive drug–polymer interactions to yield a physically stable ASD. The developed models, validated through Leave-One-Out Cross-Validation, demonstrated good predictability of stability trends. Hence, the high-throughput 2D inkjet printing technique that was used to manufacture the microarrays proved valuable for assessing drug–polymer crystallization onset risks and predicting stability outcomes. In conclusion, this study demonstrates a novel approach to solid dispersion formulation physical stability screening, enhancing efficiency, minimizing material requirements, and expanding the range of samples evaluated. Our findings provide insights into the critical physicochemical properties influencing ASD stability, offering a significant advancement in developing stable ASDs.

## Introduction

Around 40% of drugs in the market and up to 75% of new chemical entities (NCE) in small-molecule drug pipelines have poor water solubility issues and, hence, face limitations in formulation approaches, clinical applications, and marketability due to their reduced bioavailability and therapeutic effectiveness.^1,2^ This increases the risk of delay and failure in NCEs’ formulation development.

Several formulation techniques have been investigated to address the supersaturation of poorly aqueous drugs in the gastrointestinal tract (GIT), dissolution, and bioavailability, including crystal form modification, particle size reduction, lipid formulations, and amorphous solid dispersions.^3^ Solid dispersion formulations have gained interest in tailoring the physicochemical properties of drugs, including a meaningful increase in drug dissolution and suitable supersaturation conditions in the GIT.^4,5^ This has been achieved through dispersing the drug as small particles or as a molecular dispersion in an inert water-soluble carrier, commonly a hydrophilic polymer, to present the drug in an amorphous form and thus produce an improved dissolution profile compared to its crystal counterpart.^5,6^ Solid dispersions have been used successfully to enhance drugs’ bioavailability in various marketed products, as summarized in Table 1. Despite the advantages of solid dispersions, there are still few commercially available products due to challenges in design and manufacture, such as scaling up and formulations’ physicochemical instability, which can cause phase segregation and API recrystallization, especially during storage. Such recrystallization reduces the dissolution behavior of the drug in the GIT due to its conversion from the amorphous to crystalline form. In an effective solid dispersion, the polymer stabilizes the drug in the amorphous drug state sufficiently to achieve a suitable shelf life and then high supersaturation levels in the GIT through dissolution on administration. Due to the time scale for stability assessments and the preparation of solid dispersions utilizing large-scale methods, developing solid dispersions can also be challenging due to the amounts of material required and the time required.^1,7^

**Table 1:** Summary of Marketed Solid Dispersions in Oral Dosage Forms, Including the Trade Name, the APIs they Contain, the Polymer Carrier Used, Different Uses and the Manufacturer (Modified from^1,8,13−16^)^a^

Trade name	API/Drug	Polymer Carrier	Class/Use	Manufacturer
Belsomra	Suvorexant	PVPVA	Insomnia	Merck
Cesamet	Nabilone	PVP	Anticancer	Valeant Pharmaceuticals
Crestor	Rosuvastatin	HPMC	Hyperlipidemia	AstraZeneca
Cymbalta	Duloxetine	HPMCAS	Depression	Lilly
Epclusa	Sofosbuvir/Velpatasvir	PVPVA	Chronic hepatitis C	Gilead Sciences Ireland
Erleada	Apalutamide	HPMCAS	Anticancer	Janssen
Fenoglide	Fenofibrate	PEG	Hyperlipidemia	Valeant Pharmaceuticals
Gris-PEG	Griseofulvin	PEG	Fungal infection	VIP Pharma
Kaletra	Lopinivir and Ritonavir	PVPVA	Protease inhibitor (AIDS virus)	AbbVie Ltd.
Kalydeco	Ivacaftor	HPMCAS	Cystic fibrosis	Vertex
Lynparza	Olaparib	PVPVA	Anticancer	AstraZeneca
Modigraf	Tacrolimus	HPMC	Immunosuppressant	Astellas Pharma
Nimotop	Nimodipine	PEG	Calcium antagonist	Bayer AG
Onmel	Itraconazole	HPMC	Onychomycosis	Merz Pharma
Orilissa	Elagolix	PVP	Endometriosis pain	AbbVie
Zelboraf	Vemurafenib	HPMCAS	Anticancer	Roche

a PVP: polyvinylpyrrolidone, HPMC: hydroxypropylmethylcellulose, PEG: polyethylene glycol, HPMCAS: hydroxypropylmethylcellulose acetyl succinate, and PVPVA: Polyvinylpyrrolidone vinyl acetate.

Hot melt extrusion and spray drying are the most commonly used manufacturing techniques for solid dispersion formulations. These are generally relatively easy to scale up and produce well-mixed dispersions.^8,9^ However, each method requires a substantial amount of carrier-API formulation at the screening stage and significant amounts of material, which potentially are unavailable in early stage development.^10,11^ The carrier polymer itself is crucial to achieve suitable physical stability of solid dispersions due to the need for miscibility between the drug and the polymer to limit molecular drug mobility and, hence, nucleation/crystallization processes that lead to physical instability.^1,12^

Assessment in practice is typically based on trial and error evaluations of bulk samples applying a single drug with a small range of polymers for a very limited number of selected drug/polymer compositions.^7^ It would, hence, be helpful to develop reliable and relatively rapid screening processes that do not require large amounts of the drug to produce an extensive library of poorly soluble drugs with polymers.

In this regard, Eerdenbrugh and Taylor have developed a small-scale screening method to investigate the ability of seven polymers to inhibit the crystallization of eight model compounds using seven drug–polymer ratios for each compound with individual polymer.^7^ Films were prepared by rapid evaporation from the solution, applying a spin coating method as 200 μL of drug–polymer solution was spread out the coverslips. Their results were compared to those of bulk powders prepared by a rotatory evaporator, concluding that miniaturized screening could be considered a powerful technique for evaluating drug–polymer chemistry’s role in stabilizing amorphous solid dispersions.^7^ Nonetheless, this method has some limitations, such as ample storage space for the prepared samples and difficulty automating formulation preparation and controlling the deposited amount of materials on the coverslips.

Tanabe et al. developed a nanospot approach that allows for nanogram-scale evaluation of the crystalline form through some modifications to the nanospot method previously reported in the literature. This improved nanospot approach could be applicable for screening API polymorphs or new crystal forms (salts, solvates), not only cocrystals. Additionally, the newly adopted low-frequency Raman spectroscopy made detecting the crystalline form possible.^17^

High-throughput methods such as using well plates^18,19^ and inkjet printing are promising techniques for screening and formulating different APIs.^20,21^ Inkjet printing is an umbrella term encompassing a wide range of approaches to the digitally controlled formation and placement of small liquid drops.^21^ Many diverse materials have been successfully printed by inkjet, including genes,^22,23^ cells,^24^ proteins,^25^ colloids,^26^ curable-antifouling monomers,^27^ polymers,^28^ screening of polymer features to assess their suitability with the microarray manufacturing^29^ nanomaterials and pharmaceutical formulations.^30,31^ Scoutaris et al. demonstrated the feasibility for printing and rapid screening of pharmaceutical cocrystals for the first time by exploring a wide range of parameters.^32^ Liberski et al. established a link between the high-throughput 2D printing technique and the crystallization screening of commercial drugs as a high-throughput method for studying polymorphism in small molecules was presented. This technique allowed three small molecule compounds to be screened with 128 polymers consuming approximately 27 μg of each polymer and around 3.5 mg of selected drug compound for the whole experiment.^20^

Inkjet printing technology has been exploited, especially in the area of new biomaterials^33^ and drug discovery, namely, combinatorial chemistry and high-throughput screening.^21,34^ However, research in screening amorphous solid dispersion is limited. Taresco et al.^35^ proposed a new screening process capable of combining many of the advantages of the previous screening methods, namely, the miniaturization,^7^ the addressability, and the high throughput.^20^ This approach potentially offers significant efficiency in pharmaceutical formulation screening, with each experiment in the microarray format requiring samples of 3 to 6 orders of magnitude lower than conventional screening methods.^35^

The choice of polymer carrier and processing method is crucial to ensuring the stability of the solid dispersion. The formulation route for a new poorly soluble drug is still primarily determined by the formulator’s expertise and increasingly by experimental screening assays that might be significantly enhanced by a knowledge-based computational tool able to predict an optimum formulation technique.^36,37^ Assistance tools can be utilized to speed up this process, including guidance maps, high throughput screening, and statistical models.^38^ Although significant work has been done in creating such tools, their accessibility is relatively restricted.^37,39^ A number of models were developed to predict the glass-forming ability (GFA), with some also predicting the stability of APIs in their amorphous form.^37,40−44^ Additionally, glass transition temperature (*T*_g_) was predicted using molecular dynamics (MD) simulations and used to model interactions between drugs and polymers in solid dispersions and the impact of water in the system.^45−49^ Hansen solubility parameters or the Flory–Huggins equation have also historically been used theoretically to assess interactions and miscibility and predict likely stability.^50−52^ However, recent work using a high-throughput method which examined nine drugs and six polymers has called into question the applicability of this approach.^53^ Nurzyńska et al. were the first to develop a model to successfully predict the long-term amorphous stability of neutral poorly soluble drugs based on calculated, predicted, and measured parameters for 25 typical compounds utilizing the variables with the strongest correlations with the amorphous stability.^41^ Furthermore, 60 solid dispersion formulations were formulated using ten poorly soluble APIs and three polymers by two different techniques: melt extrusion and spray drying by Fridgeirsdottir et al.^52^ A single composition was employed, namely, a 10% loading of drug in each polymer. Several linear regression models were built to correlate the physicochemical characteristics of the API with the stability data collected to show which processing technique and polymer carrier combination were the most likely to produce a stable solid dispersion at 10% drug loading.^52^

The current study aims to develop a miniaturized, high-throughput assay for screening an expanded set of drug–polymer formulations in different drug loadings using a 2D picoliter inkjet printer, followed by a stability study of the printed microarrays for six months in accelerated conditions. This allowed the development of multiple linear regression (MLR) models. MLR model is a statistical technique used to understand the relationship between one dependent variable and two or more independent variables.^54^ MLR assumes a linear relationship between the variables, meaning that the change in the dependent variable is expected to be a linear combination of the changes in the independent variables. In the MLR, each independent variable is assigned a coefficient that quantifies its influence on the dependent variable. The model also includes an intercept term, representing the value of the dependent variable when all independent variables are zero. The MLR models developed in our study were used to link this experimental data set (the onset of crystallization collected from the six-month accelerated conditions) with a combined set of calculated and measured molecular descriptors of the employed APIs to predict which physicochemical properties of the APIs could be correlated with the stability of ASD formulations.

## Materials and Methods

### Model Drugs and Polymer

**Figure 1:**
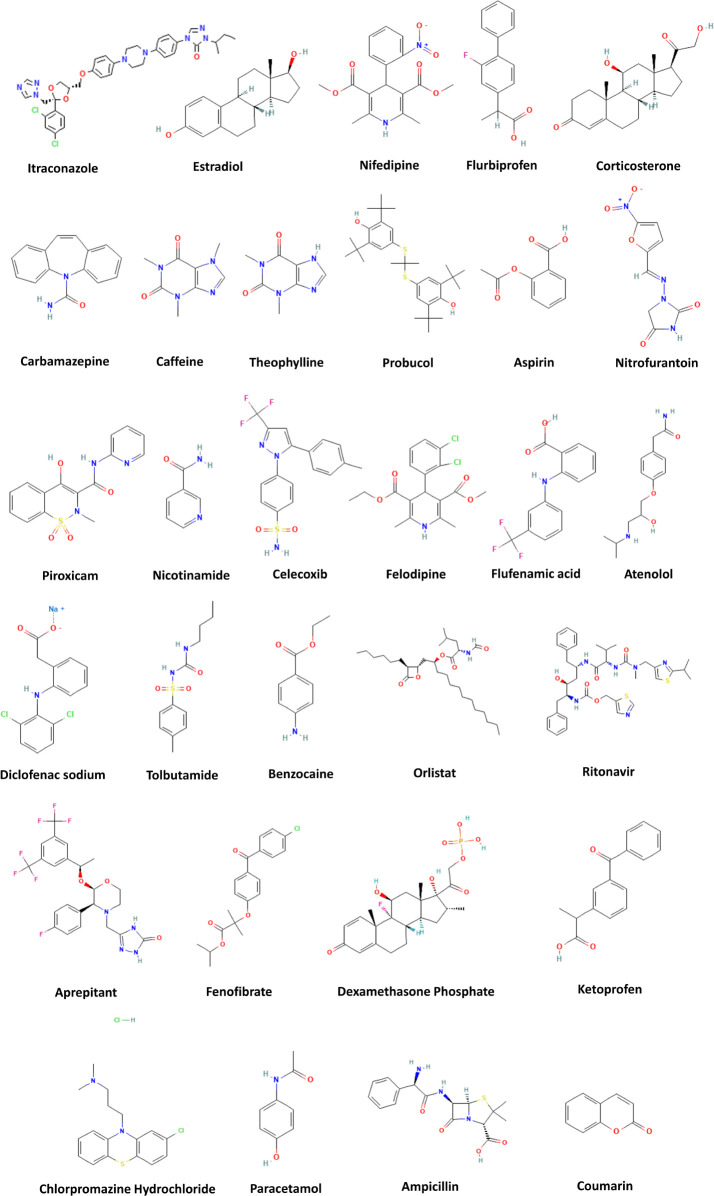
Chemical structures of the APIs used in this study.^55^

Structures of the model drugs selected for this study are listed in Figure 1. The chosen compounds have diverse chemistries and include examples of different functional groups with varying molecular weights. PVPVA was selected as the soluble matrix polymer because it is commonly available commercially, inexpensive, and widely utilized in the literature for this purpose. It is also frequently used in marketed solid dispersions, as shown in Table 1. The APIs were Nifedipine (CAS 21829–25–4), Celecoxib (CAS 169590–42–5), Fenofibrate (CAS 49562–28–9), Orlistat (CAS 96829–58–2), Flurbiprofen (CAS 5104–49–4), Acetylsalicylic acid (Aspirin ≥98%) (CAS 50–78–2), Caffeine (anhydrous) (CAS 58–08–2), Atenolol (≥98%) (CAS 29122–68–7), Carbamazepine (CAS 298–46–4), Tolbutamide (CAS 64–77–7), Piroxicam (≥98%) (CAS 36322–90–4), Theophylline (CAS 58–55–9), Flufenamic acid (CAS 530–78–9), Nicotinamide (≥99.5%) (CAS 98–92–0), Corticosterone (CAS 50–22–6), and Nitrofurantoin (CAS 67–20–9) were purchased from Merck life science UK Ltd. Felodipine (CAS 72509–76–3) was obtained from Carbosynth Ltd., UK. Ritonavir (CAS 155213–67–5), Itraconazole (CAS 84625–61–6), and Aprepitant (CAS 170729–80–3) were purchased from Acros Organics, Fisher Scientific UK Ltd. B-Estradiol (CAS 50–28–2) was purchased from AlfaAesar, Thermo Fischer Scientific, UK. Probucol (CAS 23288–49–5) was obtained from MP Biomedicals, LLC, France. Diclofenac sodium salt (CAS 15307–79–6) was purchased from MP Biomedicals, LLC, Germany. Dexamethasone phosphate (CAS 2392–39–4), Ketoprofen (CAS 22071–15–4), Chlorpromazine hydrochloride (CAS 69–09–0), Paracetamol (CAS 103–90–2), Ampicillin (CAS 69–53–4), and Coumarin (CAS 91–64–5) were purchased from Merck life science UK Ltd. All APIs were of high purity (98–99%) and were used as received without further processing.

**Figure 2:**
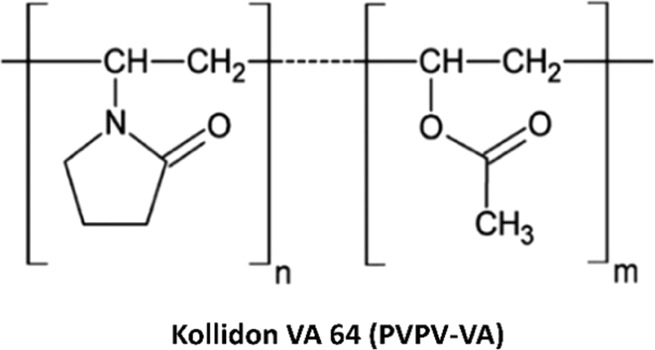
Chemical structure of the polymer used in this study.^56^

Polyvinylpyrrolidone-vinyl acetate copolymer K 28 or Copovidone 64 as Kollidon VA 64 Fine (PVP-VA) (CAS 25086–89–9) with molecular weight (45,000–70 000 Da) was received as a generous gift from BASF SE, Germany (chemical structure is shown in Figure 2). Sodium chloride (CAS 7647–14–5) was purchased from Acros Organics, Fisher Scientific UK Ltd., to create the 75%RH accelerated condition for microarrays storage. Dimethylformamide (DMF) > 99.5% (CAS 68–12–2) HPLC grade as a working liquid to enable control of system pressure in the printer was obtained from Fisher Scientific UK Ltd. Dimethyl sulfoxide (DMSO) > 99.5% (CAS 67–68–5) was purchased from Sigma-Aldrich, France, and was used as a common solvent to dissolve all the printable materials and their blends. Polycrystalline gold with 30 nm film thickness on special flat glass 25 mm × 75 mm in size, precoated with titanium for better adhesion; roughness <1 nm) was used as a substrate for the printing and was purchased from George Albert PVD- Beschichtungen, Silz (Germany). Gold-coated glass slides were chosen as they provide a surface with a higher contact angle than bare glass slides, limiting printed droplet splashing and spreading. 384-well polypropylene microplates with flat bottoms were purchased from Greiner Bio-One Ltd. and were used as reservoirs to contain the stock solutions of drugs and polymers.

### Piezoelectric Inkjet Printing

**Figure 3:**
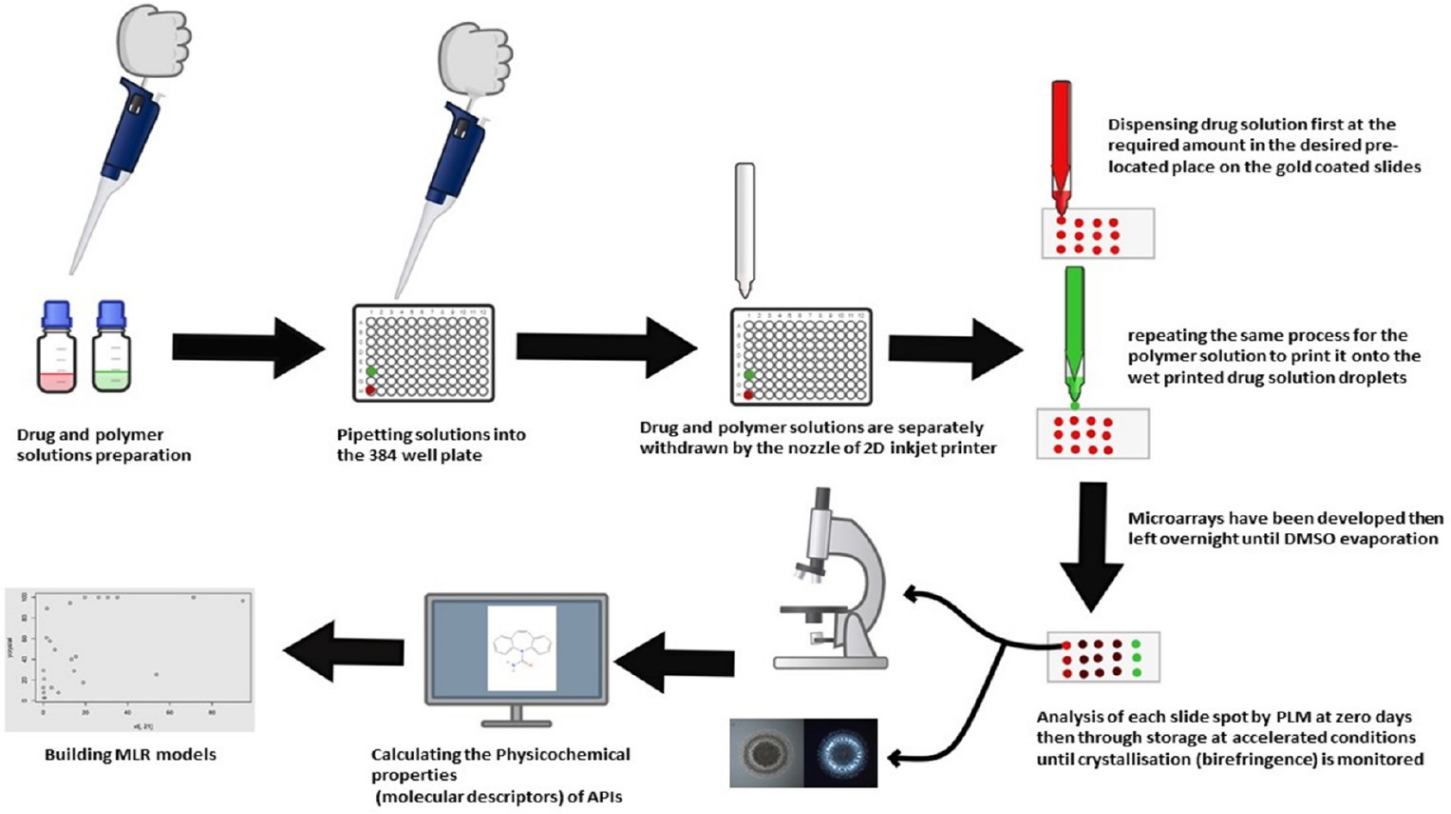
Schematic representation of steps followed in manufacturing and analyzing microarrays, including drug and polymer solutions preparation, pipetting into well-plate, withdrawing drug solution through the printer nozzle and then dispensing at the required amount in the desired prelocated place on the substrate followed by repeating the same process for the polymer solution to print it onto the printed drug solution droplets.

A miniaturized, high-throughput technique was used to print microarrays of the drug–polymer mixtures using a piezoelectric inkjet printer Sciflexarrayer S5, Scienion (inkjet printer software sciflexarrayer (Scienion A, version 2.09.002)), and 70 μm orifice nozzle type 2 coating (Scienion, Germany). The droplet size was controlled and optimized by adjusting the voltage and electrical pulse values. As depicted in Figure 3, each drug and polymer were dissolved separately in DMSO to reach a final concentration of 10 mg/mL. The prepared solutions were sonicated for 5 min to aid the dissolution. Spots of 500 droplets with an average droplet volume of 200 pL with a final deposited mass of around 1000 ng were dispensed by adjusting the voltage between 82 to 90 V and the pulse duration between 48 to 58 μs. DMF was used to wash the nozzle between each printing cycle as a part of an automated printing washing program to avoid cross-contamination of the samples. Printed microarrays were stored in the printer chamber overnight at around 25 °C and 55% RH to allow the DMSO to evaporate. The dried printed microarrays were monitored by polarized light microscopy (PLM).

**Figure 4:**
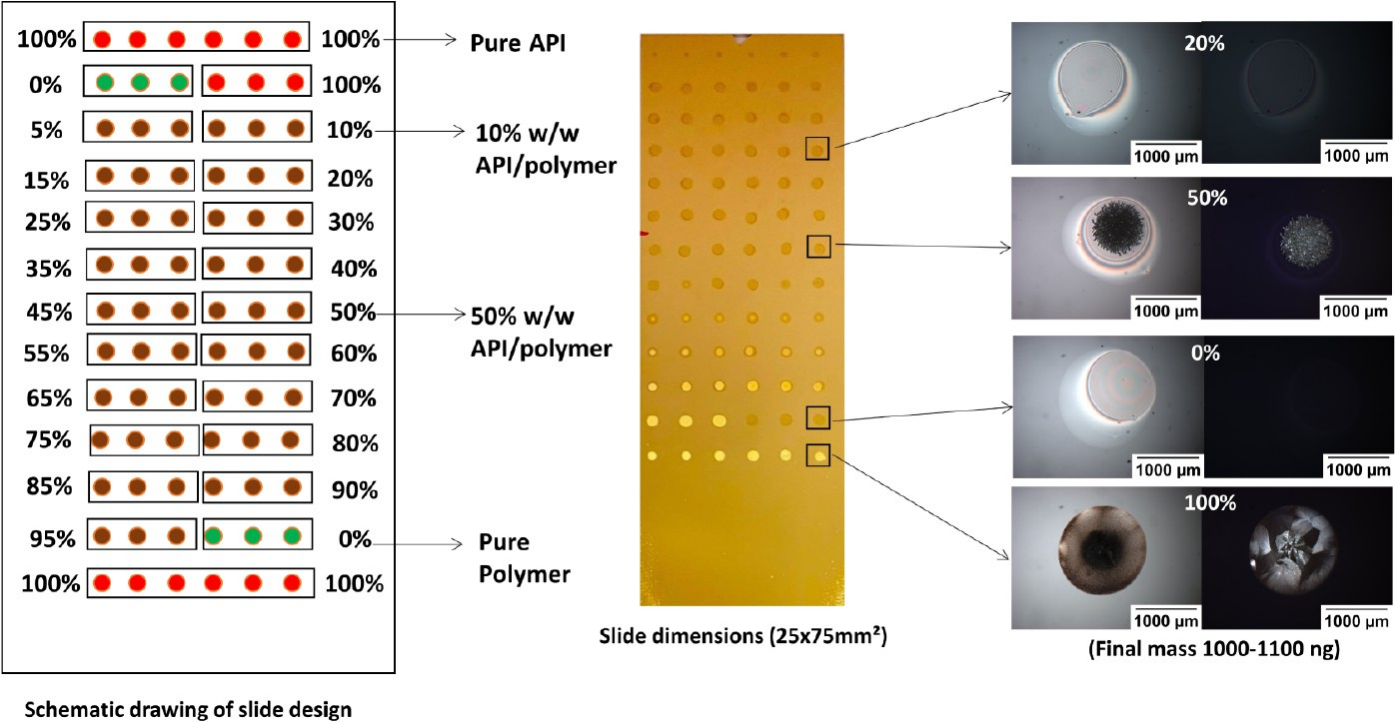
A schematic drawing of the slide design is shown on the left side of the Figure, as API and polymer were printed as a reference in the top and bottom two rows. In between, API/polymer mixtures were printed in triplicates starting from 5% with 5% w/w increment until 95%. An image of a printed microarray of Estradiol and PVPVA as a model for the APIs used in the study is shown in the middle. On the right side, microscopic images using bright (left) and cross-polarized field (right) of four printed spots are shown as examples of the crystallization assessment, as the first and third rows (0%, 20%) show no birefringence (no crystallization). The second and fourth rows (50%, 100%) show the presence of birefringence (crystallization). Images shown here were taken after six months of storage in accelerated conditions (75% relative humidity and 40 °C in a stability oven). The dimensions of gold-coated glass slides are (25 × 75 mm^2^).

Microarrays were designed using the calculated amounts of each drug solution printed onto the gold-coated slides, subsequently combined with calculated amounts of polymer to give microdots containing different drug/polymer ratios in w/w ranging from 5 to 95% in 5% increments, as shown in detail in Figure 4. Each drug–polymer loading was printed in triplicate.

### Stability Testing

Following ICH guidelines,^57^ a six-month stability analysis was carried out on all formulations (printed microarrays) under accelerated conditions (40 °C ± 2 °C/75% RH ± 5% RH) to monitor the physical stability of different printed microarrays of drug/polymer ratios. The microarray slides were kept in a closed plastic box inside the stability oven, and a data logger, a Thermopro TP49, was used to monitor the environment.

### Polarized Light Microscopy (PLM)

In this study, PLM was utilized as the primary detection method due to its sensitivity and speed in identifying small amounts of crystalline material within amorphous matrices. The formulation state was initially assessed using PLM (Advanced Polarizing HS1 microscope, Prior LuxPOL), mainly employing a 4× objective to detect the appearance of birefringence, indicating crystallization, or its absence, indicating amorphicity. This instrument has an integrated 30 W halogen lamp with variable brightness control polarized light source to detect the presence of birefringence in the printed spots to interpret crystallinity, a routine use in the pharmaceutical field.^58^ Microscopic images were taken with and without cross-polarized filters for the printed spots, including pure drugs and various drug–polymer mixtures were recorded within 24 h of printing as zero time and at different intervals during six months of exposure to 40 °C/75% RH. They were examined every day for the first 2 weeks and then every week for the following six months.

### Evaluation of Crystallization Behavior

The onset of crystallization of each printed polymer blend was determined from the number of days until the first obvious birefringence was observed within the PLM investigation of the spot, as the birefringence is considered the sign of drug crystallization from the polymeric matrix.

Drug–polymer miscibility refers to the ability of the drug to uniformly disperse within a polymer matrix, forming a single phase without phase separation or crystallization. In this study, the crystallization onset risk was established as an indication of this miscibility by identifying the highest drug/polymer loading where no crystallization was observed and above which birefringence was detected in the printed sample. This limit indicates the polymer ratio capable of preventing API crystallization. The crystallization onset risk was confirmed by the appearance of birefringence at the following two higher drug/polymer loadings for each API-PVPVA system; for example, if the crystallization onset risk (no birefringence detected) was 20%, the results were confirmed by the appearance of birefringence at 25 and 30% in the printed microarrays.

### Drop-Cast Method of Drug/PVPVA Dispersion

The drop-cast technique involves placing a controlled volume of solution onto a substrate and allowing the solvent to evaporate over time, forming a solid film. This slow evaporation process and the longer drying time allow maximum molecular rearrangement, increasing the likelihood of crystallization. In contrast, a faster evaporation rate in the case of inkjet printing reduces the time available for molecular rearrangement, which may lead to the formation of an amorphous form instead of a crystalline. The drop cast method was employed in this study to compare the relative crystallization behavior of the printed nanoscale formulation with the milligram scale formulation and to investigate the influence of different evaporation rates on drug crystallization and the formation of solid dispersions.

Pure drugs as well as three drug/PVPVA ratios were chosen for this method. Using a micropipet, 200 μL of each solution was dropped onto gold-coated glass slides to form large spots of final mass around 2 mg, done in triplicates. The gold-coated slides were left in the printer cage for 3–4 days to allow complete DMSO evaporation, which took longer than the printed microarray spots due to the scaled-up size of the experiment. Then, the dried drops were analyzed by PLM, as previously mentioned.

### Calculated Physicochemical Properties of APIs

The selection of parameters for the modeling process was based on the principle of maximum diversity, utilizing all available descriptors to capture a broad range of physicochemical properties. There was a careful consideration of the physicochemical properties known to influence the behavior, interactions, and characteristics of molecules in pharmaceutical and chemical contexts, as well as the parameters that have been used in the other modeling for predicting the stability of solid dispersions on smaller data sets in the literature.^41,52^ Each parameter is computationally accessible and interpretable, minimizing the need for additional experiments while allowing meaningful correlations with modeled outcomes. This ensures that the results are scientifically robust and scientifically grounded.

While glass-forming ability (GFA), an experimentally derived parameter from DSC data, is a key factor in predicting crystallization, it may not be sufficient alone to predict outcomes. Although all the drugs in our study had similar GFA classes, they exhibited different behaviors. This highlights the importance of additional factors, such as drug–polymer interactions, which are critical in preventing crystallization. Even drugs with moderate GFA can remain amorphous with a compatible polymer that enhances miscibility. External conditions like temperature and humidity also influence crystallization and miscibility limits. Thus, GFA should be considered alongside environmental factors and drug–polymer interactions to predict crystallization more accurately.

The program Chemicalize from ChemAxon^59^ and ChemSpider^55^ was used to calculate and predict the physicochemical properties of the different APIs based on the molecular structure of each API. The physicochemical properties of the used APIs are shown in Table 2.

**Table 2:** Physicochemical Parameters of the APIs Used in This Study as Calculated from Chemicalize from Chemaxon^59^ and Their Melting Points as Obtained from ChemSpider^55^

property	Caffeine	Theophylline	Carbamazepine	Piroxicam	Corticosterone	Nitrofurantoin	Diclofenac Sodium	Atenolol	Itraconazole	Nicotinamide	β-Estradiol	Nifedipine
HBD	0	1	1	2	2	1	1	3	0	1	2	1
HBA	3	3	1	5	4	5	3	4	9	2	2	5
LogP	–0.55	–0.77	2.95	0.39	2.02	–0.22	4.26	0.425	7.311	–0.39	3.75	1.815
MW	194.2	180.17	236.27	331.35	346.5	238.2	318.13	266.34	705.6	122.13	272.4	346.34
mp	236	273	190	199	180	270	288	149	166.2	130	176	173
nChir	0	0	0	0	7	0	0	0	3	0	5	0
nHet	6	6	3	8	4	9	5	5	14	3	2	8
sp^2^	5	5	15	14	4	7	13	7	22	6	6	12
sp^3^	3	2	0	1	17	1	1	7	13	0	12	5
nAliphRing	0	0	1	1	4	1	0	0	2	0	3	1
nArRing	2	2	2	2	0	1	2	1	5	1	1	1
nHetRing	2	2	1	2	0	2	0	0	4	1	0	1
LogS_0_ (M) at 7.4	–0.44	–0.82	–3.79	–0.93	–3.68	–3.21	NA	0.3	–7.72	–0.38	–3.99	–2.9
LogS_0_ (M)	–0.44	–0.96	–3.79	–3.27	–3.68	–3.27	NA	–1.57	–7.72	–0.38	–3.99	–2.9
ASA (Å^2^)	368.4	323.99	370.91	489.16	425.2	416.5	433.35	535.74	992.1	276.72	395.1	533.46
nAtom	24	21	30	36	55	23	30	41	87	15	44	43
avPol (Å^3^)	17.87	16.13	26.79	32.56	37.64	19.24	28.92	29.09	71.59	12.28	31.31	33.98
HLB	12.1	12.39	10.6	19.99	4.34	19	24.27	19.15	5.5	14.5	3.787	19.25
nC	8	7	15	15	21	8	14	14	35	6	18	17
nCl	0	0	0	0	0	0	2	0	2	0	0	0
nF	0	0	0	0	0	0	0	0	0	0	0	0
nN	4	4	2	3	0	4	1	2	8	2	0	2
nO	2	2	1	4	4	5	2	3	4	1	2	6
nS	0	0	0	1	0	0	0	0	0	0	0	0
nRing	2	2	3	3	4	2	2	1	7	1	4	2
nRot	0	0	0	2	2	3	4	8	11	1	0	6
tPSA (Å^2^)	58.44	69.3	46.33	99.6	74.6	118.1	52.16	84.58	100.8	55.98	40.46	107.77
vdW-SA (Å^2^)	269.1	235.19	312.24	411.56	543.6	279.9	359.64	440.26	965.4	171.42	436.6	466.23
Fsp^3^	0.37	0.29	0	0.07	0.81	0.12	0.07	0.5	0.37	0	0.67	0.29
MRef (cm^3^/mol)	49.83	44.93	71.89	87.04	96	52.11	86.3	73.51	200.4	32.98	79.9	91.61
nHeavy	14	13	18	23	25	17	20	19	49	9	20	25
nAsymmetric	0	0	0	0	7	0	0	1	3	0	5	0
FC	0	0	0	0	0	0	0	0	0	0	0	0
vdW-Vol (Å^3^)	164.3	146.69	210.15	267.89	338.5	174.8	234.5	261.34	603.9	108.08	269.7	299.78
MinProjArea (Å^2^)	30.01	28.75	40.19	39.47	48.89	31.59	44.3	36.85	99.42	18.73	37.85	57.61
MaxProjArea (Å^2^)	61.86	56.51	68.29	94.14	96.43	62.05	77.08	87.58	155.9	44.32	84.51	87.31
MinProjRad (Å)	4.44	4.34	4.48	4.95	4.66	3.76	4.71	4.19	7.02	3.3	4.02	6.22
MaxProjRad (Å)	5.03	4.98	5.76	7.82	7.96	6.97	5.91	9.12	12.51	4.72	7.3	6.46

property	Flurbi profen	Flufen amic acid	Tolbut amide	Aspirin	Pro bucol	Celecoxib	Felodipine	Ritonavir	Fenofibrate	Aprepitant	Orlistat
HBD	1	2	2	1	2	1	1	4	0	2	1
HBA	2	3	3	3	2	3	3	6	3	6	3
LogP	3.94	5.25	2.3	1.24	10.57	4.01	3.44	5.22	5.278	4.5	8.11
MW	244.3	281.2	270.35	180	516.8	381	384	721	360.8	534	495.8
mp	111	134	129	139	125	158	145	121	81	250	45
nChir	1	0	0	0	0	0	1	4	0	3	4
nHet	3	6	6	4	4	9	7	13	5	14	6
sp^2^	13	13	7	8	12	15	12	21	14	14	3
sp^3^	2	1	5	1	19	2	6	16	6	9	26
nAliphRing	0	0	0	0	0	0	1	0	0	1	1
nArRing	2	2	1	1	2	3	1	4	2	3	0
nHetRing	0	0	0	0	0	1	1	2	0	2	1
LogS_0_ (M) at 7.4	–1.96	–1.02	–3.38	–1.9	–10.5	–5.5	–4	–6.24	–5.86	–5.3	–9.98
LogS_0_ (M)	–4.24	–4.34	–3.38	–1.9	–10.5	–5.5	–4	–6.24	–5.86	–5.5	–9.99
ASA (Å^2^)	436.5	445.9	554.15	369	874.6	603	581	982	643.5	700	985.3
nAtom	31	30	36	21	83	40	44	98	46	58	88
avPol (Å^3^)	26.96	24.52	27.81	17.2	61.95	36.4	37	75.7	38.18	43	56.15
HLB	4.74	5.65	11.79	10.1	3.059	10	13.8	19.3	5.73	11	8.07
nC	15	14	12	9	31	17	18	37	20	23	29
nCl	0	0	0	0	0	0	2	0	1	0	0
nF	1	3	0	0	0	3	0	0	0	7	0
nN	0	1	2	0	0	3	1	6	0	4	1
nO	2	2	3	4	2	2	4	5	4	3	5
nS	0	0	1	0	2	1	0	2	0	0	0
nRing	2	2	1	1	2	3	2	4	2	4	1
nRot	3	4	4	3	8	4	6	18	7	8	23
tPSA (Å^2^)	37.3	49.33	75.27	63.6	40.46	78	64.6	146	52.6	75	81.7
vdW-SA (Å^2^)	347.4	348.15	421.08	246	916.6	485	493	1066	533.6	658	925.7
Fsp^3^	0.13	0.07	0.42	0.11	0.61	0.12	0.33	0.43	0.3	0.4	0.9
MRef (cm^3^/mol)	67.29	67.77	70.27	44.5	159.3	92.2	99.2	195	97.13	127	139.9
nHeavy	18	20	18	13	35	26	25	50	25	37	35
nAsymmetric	1	0	0	0	0	0	1	4	0	3	4
FC	0	0	0	0	0	0	0	0	0	0	0
vdW-Vol (Å^3^)	219.2	223.8	244.75	155	534.2	299	322	663	325.4	414	529.4
MinProjArea (Å^2^)	36.95	36.51	51.75	33.3	85.41	58.3	60.7	120	45.12	79	81.63
MaxProjArea (Å^2^)	72.36	80.4	72.98	56	131	93.3	87.6	163	102.6	115	157.4
MinProjRad (Å)	4.27	4.65	5.08	4.5	6.27	6.19	5.6	8	4.33	6.9	7.66
MaxProjRad (Å)	6.98	6.59	7.24	5.15	8.56	7.33	6.3	9.48	9.72	8.3	13.13

The following abbreviations are used: MW, molecular weight; LogP, lipophilicity; HBA, number of H-bond acceptors; nRing, number of rings; HBD, number of H-bond donors; nAtom, number of atoms; nChir, number of chiral atoms; tPSA, topological polar surface area; ASA, water accessible surface area; HLB, hydrophilicity–lipophilicity balance; nCl, number of chlorine atoms; vdW-SA, van der Waals surface area; vdW-Vol, van der Waals volume; LogS_0_, water solubility as log_10_(molar solubility); mp, melting point; nAliphRing, number of aliphatic rings; nArRing, number of aromatic rings; nO, number of oxygen atoms; nC, number of carbon atoms; nF, number of fluorine atoms; avPol, average polarizability; nRot, number of nonterminal rotatable bonds; nS, number of sulfur atoms; nHet, number of heteroatoms (atoms other than carbon and hydrogen); nN, number of nitrogen atoms; sp^3^, number of sp^3^-hybridized carbon atoms; sp^2^, number of sp^2^-hybridized carbon atoms; nHetRing, number of heterorings; nHeavy, number of heavy atoms; LogS_0_ at 7.4, water solubility as log_10_(molar solubility) at pH 7.4; Fsp^3^, number of sp^3^-hybridized carbons/total carbon count; nAsymmetric, number of asymmetric atoms; FC, formal charge (the electric charge of an atom in a molecule); MRef, molar refractivity (a measure of the total polarizability of a mole of a substance); MinProjArea, minimum projection area; MaxProjArea, maximum projection area; MinProjRad, minimum projection radius; MaxProjRad, maximum projection radius.

### Multiple Linear Regression (MLR) Model

The publicly available software R^60^ and R Studio (version 1.0.143) were used to build multiple linear regression models to fit the experimental data. The calculated and measured parameters, shown in Table 2, were used as input variables in the model equations. Stability data was a record of when, in days, a given formulation exhibited textured opaque areas in transmitted light microscopy, producing birefringence in cross-polarized optical microscopy. A MLR model is a statistical technique used to understand the relationship between one dependent variable and two or more independent variables.^54^ MLR assumes a linear relationship between the variables, meaning that the change in the dependent variable is expected to be a linear combination of the changes in the independent variables. In MLR, each independent variable is assigned a coefficient that quantifies its influence on the dependent variable. The model also includes an intercept term, representing the value of the dependent variable when all independent variables are zero.

These data were inputted into the MLR workflow as a natural log of days to crystallization (number of days +1) to mitigate issues related to zero values. When a drug failed to crystallize within the observed time frame, a default value of 500 days for stability was used. MLR models were built with one model for each drug/polymer percentage. Input data of the onset of crystallization of nanoarrays are provided in Table S1. To facilitate the selection of covariates for the regression models, the “leaps” package within R was used. Following the selection of linearly independent parameters, these were employed to construct a regression model, thus establishing a linkage between the physicochemical properties of the APIs and the natural logarithms of the measured stability data of the solid dispersion formulations. Leave-one-out cross-validation (LOOCV) was conducted to assess the model’s predictability and identify the most appropriate model.

## Results and Discussion

PLM is a contrast-enhancing technique used to distinguish between crystalline and amorphous materials. Crystalline domains, being anisotropic, exhibit birefringence under polarized light, while amorphous materials, which are isotropic, show a similar color to the background. The advantage of PLM is that only small amounts of material are required; therefore, it is a valuable screening technique.^61^ PLM has been utilized in various studies to assess the crystallization behavior of amorphous systems and to determine the most suitable polymers for solid dispersions.^7,62−64^ Therefore, PLM was employed in our study as the method of choice for crystallization screening.

All solutions were filtered prior to printing, significantly reducing the likelihood of any crystalline material being present in the initial printed solutions. If any crystals had been present, it would have likely resulted in nozzle blockages during printing. Given the very thin and nanogram-scale spots printed during our study, the likelihood of crystals being obscured is minimal. We implemented a thorough monitoring approach, directing the slide in multiple orientations and utilizing various magnification powers to ensure that any absence or presence of birefringence was confirmed. Additionally, we tracked the onset of crystallization by observing the appearance of birefringence at different drug loadings over six months, proving that initial birefringence observations were consistent and not measurement artifacts. As all the analytical instruments have a detection limit, in our study, the sensitivity of the PLM was adequate for identifying crystallization events (absence or presence of birefringence) at the nanogram scale. This capability is critical for accurately assessing the crystallization behavior of our formulations.

In our study, the onset of crystallization for each polymer blend was determined by the first detection of birefringence and monitored over six months to ensure consistency. PLM also provided insight into the miscibility limit by identifying the drug-to-polymer ratio at which no birefringence was detected and above which birefringence first appeared. Parekh et al. previously utilized a similar definition for drug–polymer miscibility using a film-casting method.^65^

Different solvents could significantly affect the stability of the ASDs. Therefore, solvent selection is a critical factor that influences the behavior of the formulation and must be carefully considered when evaluating ASDs. In this study, drugs and polymers were prepared in DMSO, which was the solvent of choice for printed microarrays, as it has low volatility, thus reducing the likelihood of nozzle clogging.^35^ Additionally, it is a common solvent for many drugs and polymers.^66,67^ It has high surface tension, thus, forming droplets on the substrate with a high contact angle and minimal droplet spreading. Gold-coated slides were used as substrates due to the high-water contact angle for the gold surface (79.5° ± 0.41), which aided the production of well-formed microdots after evaporation of the DMSO.

The inkjet printing predictions in our study are based on solvent-based manufacturing with DMSO selected for its compatibility with the 2D inkjet printer and its effectiveness in dissolving both drugs and polymers. Using the same solvent for all APIs and formulations ensured consistent conditions, allowing for reliable comparisons. This uniformity minimized variables such as differing evaporation rates or solubility, enabling accurate assessment of crystallization behavior and miscibility of the APIs in the PVPVA polymer matrix. This approach contributed to the stability and reproducibility of the results across the formulations.

To ensure the homogeneous mixing of the drug and polymer solutions in the final printed spots, both were separately dissolved in DMSO at a 10 mg/mL concentration. During the inkjet printing process, which ensures precise control of droplet deposition, the drug solution is first printed onto the substrate, followed by the polymer solution on top, all in nanoliter quantities. Since both drug and polymer solutions use the DMSO, molecular-level mixing occurs directly on the printed substrate as the droplets merge. The rapid evaporation of DMSO further helps to achieve uniform distribution of the drug and polymer in the small printed area, minimizing the chance of phase separation and ensuring homogeneity. The good reproducibility of our printed arrays suggested that poor mixing is not a concern. A similar approach was successfully employed in a previous study, where nanoliter amounts of drug–polymer solutions were printed to create microarrays, demonstrating effective mixing and stability of solid dispersions.^35^

Although we did not directly measure residual DMSO content using techniques such as Thermogravimetric Analysis (TGA), visual inspection confirmed that evaporation was complete within a few hours at most, which minimizes the potential for early crystallization and ensures consistent mixing of the drug and polymer on the substrate.

### Crystallization Behavior Following 2D Inkjet Printing of Pure APIs and Upon Storage

**Figure 5:**
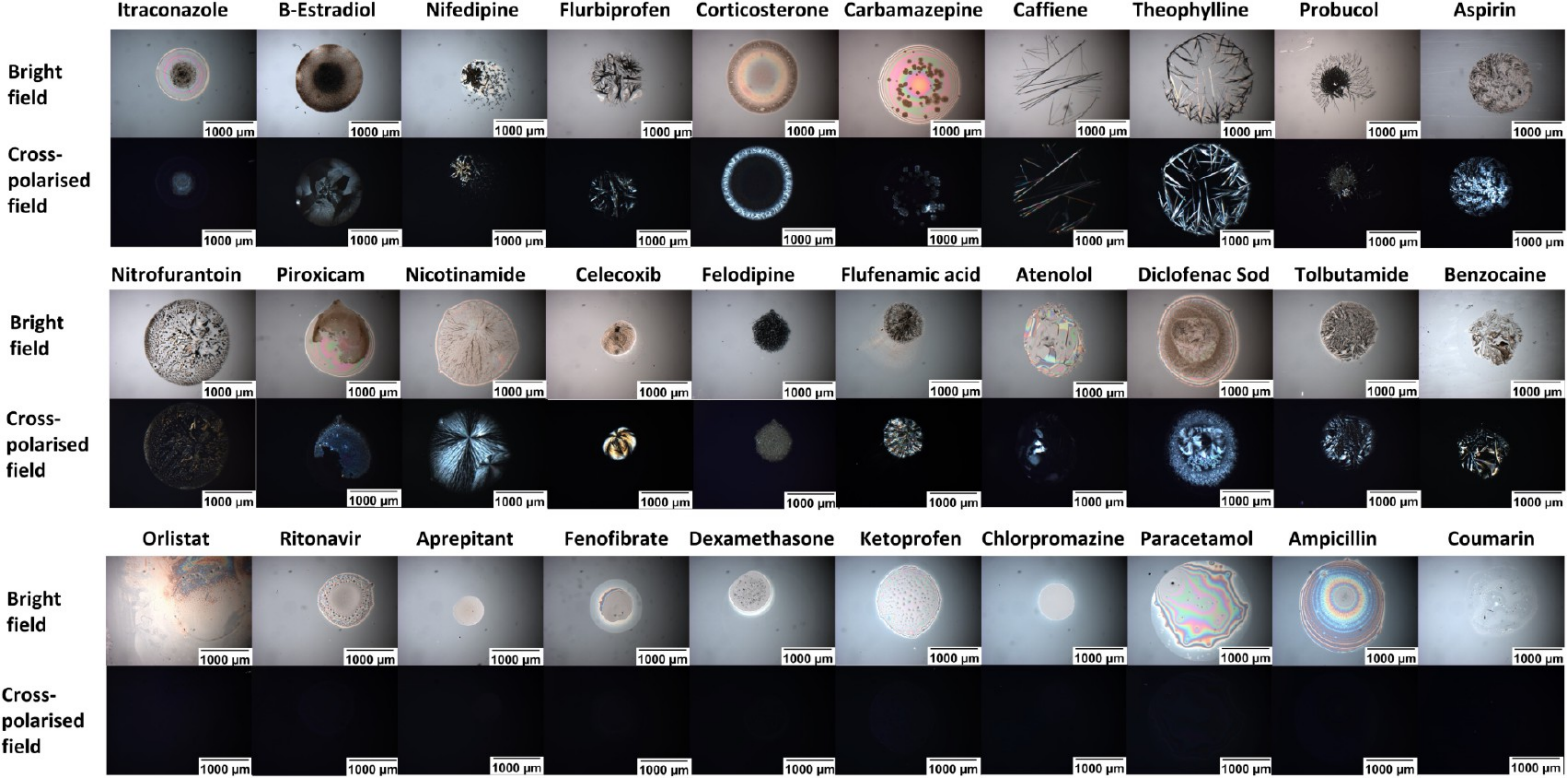
Different APIs recrystallization profiles used in this study (final mass 1000–1100 ng). All images are reported without (top row) and with (bottom row of each set) cross-polarized filters. Images of all APIs are depicted six months from solvent evaporation while storage in accelerated conditions (75% relative humidity and 40 °C in stability oven).

In our study, various pure APIs were screened for crystallization behavior. Figure 5 presents bright-field and cross-polarized images of all 30 pure drug spots six months after printing, stored at 40 °C/75% RH. The images have been arranged with samples showing opaque spots with obvious structure (bright field) and birefringence (cross-polarized) toward the top of the Figure, while spots presented as mainly transparent, with little to no structure and no birefringence at the bottom. Twenty spots which were opaque from visual inspection exhibited birefringence; the other ten, which appeared mainly transparent, exhibited no birefringence.

The presence of opaque material and structures in bright-field and birefringence under cross-polars is taken to indicate crystallization. Likewise, transparency, lack of obvious structure, and no birefringence indicate a lack of crystallization and the presence of mainly amorphous material.

Itraconazole, Estradiol, Flurbiprofen, Corticosterone, Carbamazepine, Caffeine, Theophylline, Aspirin, Nitrofurantoin, Piroxicam, Nicotinamide, Flufenamic acid, Atenolol, Diclofenac sodium, Tolbutamide, and Benzocaine showed obvious structure, opaqueness, and birefringence (signs of crystallization) in the optical and cross-polarized images through the first day after solvent evaporation. Nifedipine showed birefringence through the fifth day after solvent evaporation upon storage under accelerated conditions. Felodipine, Celecoxib, and Probucol had a comparatively extended time in the amorphous state until they showed signs of birefringence, detected within 56 days after solvent evaporation while being stored in accelerated conditions. In contrast, Orlistat, Ritonavir, Aprepitant, Fenofibrate, Dexamethasone, Ketoprofen, Chlorpromazine, Paracetamol, Ampicillin, and Coumarin did not show any signs of crystallization as they remained transparent during the six months storage in accelerated conditions.

These results can be compared in a limited manner with the work of van Eerdenbrugh et al.^68^ We first note that the storage conditions employed differ. We employed storage at 40 °C/75% RH for six months, whereas van Eerdenbrugh reported results after 7 days of storage at room temperature in very dry conditions. We would, therefore, expect that our storage conditions would provide far more opportunity for samples to crystallize than van Eerdenbrugh. Also, the two compound libraries overlap only for 14 compounds. Of the samples for which crystallization was not observed after six months in the present work, there is complete agreement with van Eerdenbrugh, who classified Paracetamol, Ketoprofen, Fenofibrate, and Ritonavir as class II or III, based on limited or no crystallization observed after 7 days. For the samples that were observed to crystallize in the present study, the results are more mixed, but this is to be expected given the differences in storage conditions between the two studies. If van Eerdenburgh did not observe crystallization within 7 days for a given sample, it might be that crystallization did happen at a later point outside this time window, and hence, the present study would potentially differ. There are no occasions on which van Eerdenburgh et al. observed crystallization but where the current research does not, indicating the consistency of the two studies. From the data and analyses presented above, we conclude that our results from miniaturized inkjet printing at ca. 1000 ng are reasonable and representative of results from a related set of more bulk-like samples prepared by spin coating. We, therefore, undertook the printing of mixed drug–polymer samples, and these results are reported below.

From our printed set of 30 APIs, 16 could be classified as Class I (rapid crystallization), 4 APIs as Class II (intermediate crystallization), and 10 as Class III (slow crystallization) using the classification scheme of Van Eerdenburgh. These data are tabulated in Table S1.

### Effect of Drug/PVPVA Ratio and Time on Drug Crystallization within the PVPVA Matrix

**Figure 6:**
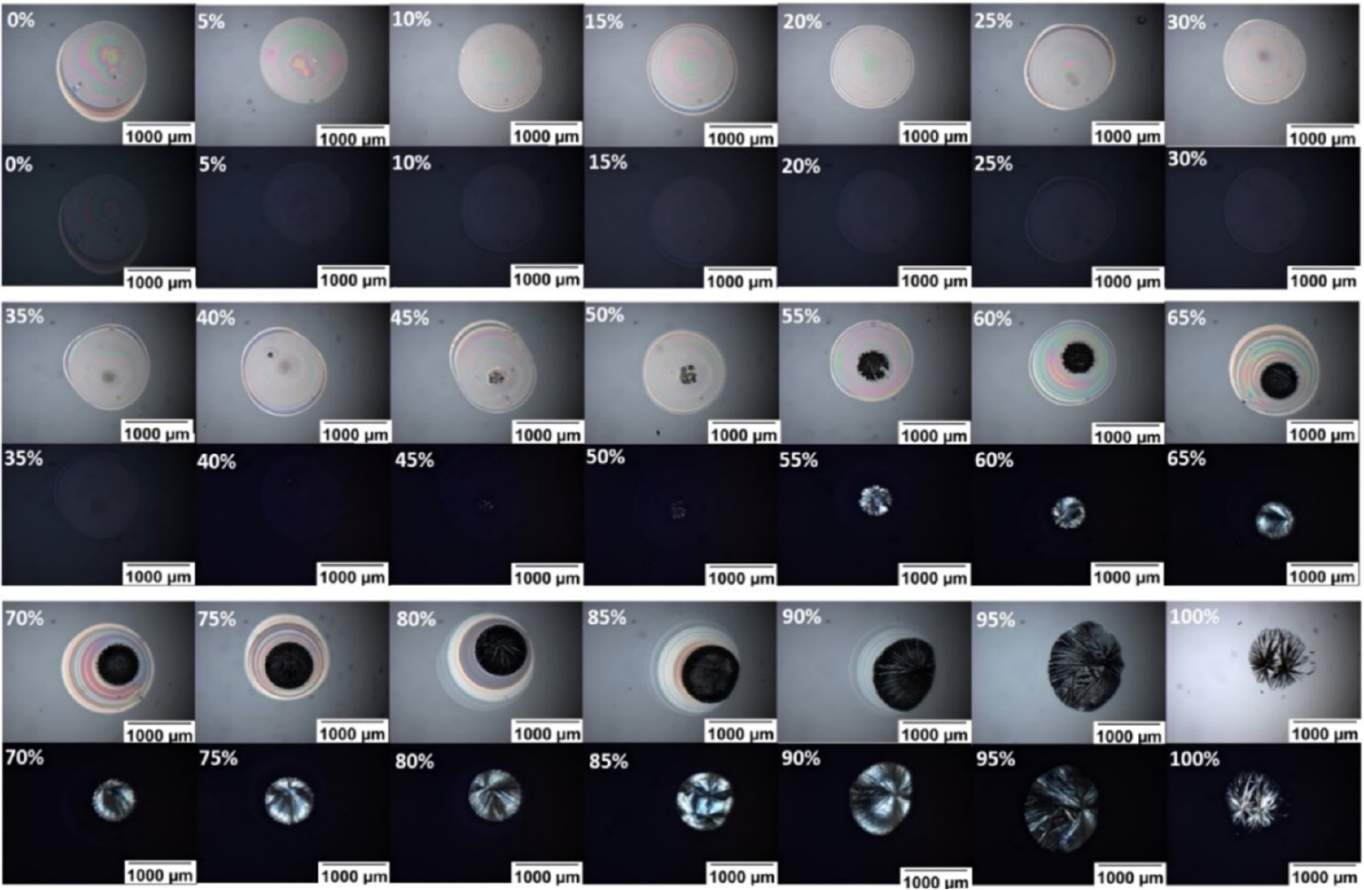
Example of an array section depicted by PLM. All of the spots are reported with bright field and cross-polarized filters (top and bottom of each section, respectively). Images of all Flurbiprofen/PVPVA (for example, as one of the drugs used in this study) at all of the different ratios (starting from 0% until 100% with a 5% increment) are depicted after 6 months of storage in accelerated conditions (75% relative humidity and 40 °C in stability oven). The final mass of individual spots is 1000–1100 ng.

All the APIs that showed crystallization through inkjet printing after six months of storage under challenge conditions were printed in the same scheme. Flurbiprofen is reported here as an example of the drug–polymer printing results, as shown in Figure 6. Detailed data on crystallization behavior and the onset of crystallization of the remaining APIs can be found in the Supporting Information. This figure shows bright-field and cross-polarized images of spots of 21 Flurbiprofen-PVPVA formulations for compositions 0, 5, 10,···95, 100% drug loading six months after printing, having been stored at 40 °C/75% RH within this period. The images have been arranged with the bright field images on the top of each section and the cross-polarized images on the bottom showing opaque/transparent structure (bright field) and presence/absence of birefringence (cross-polarized).

As the loading of Flurbiprofen increased from 0 to 100%, there is a trend for the printed spots to change from visually transparent and unstructured to visually opaque with the appearance of structure. These changes are accompanied by the emergence of birefringence. The spots up to 20% API loading are transparent; above this value, parts of the spot appear opaque, and for 95 and 100% API loading, the spots appear wholly opaque. Notably, birefringence only emerges for 45% loading and above despite the appearance of some small opaque areas in the spots for 20% API loading and above.

The appearance of birefringence is taken to be an indication of the presence of drug crystals in the spots and is therefore unambiguous evidence of API-polymer phase segregation. This is therefore interpreted as an upper estimate of the drug miscibility in the polymer. The small opaque areas for 20–40% API loadings may indicate the beginnings of phase segregation but as insufficient to lead to birefringence were taken to be below the instability threshold.

The same approach was adopted for all the other APIs with PVPVA. The time of appearance of birefringence was recorded for all samples and was used as input for developing a model linking stability (time to crystallization) with molecular and material properties. These model input data are presented in Table S2 ranked by time. Raw data used to determine the appearance of birefringence are presented in the Supporting Information.

The trend toward early crystallization of higher drug loading spots over lower drug loading spots is, as expected, given the increased likelihood of nucleation as a function of the total amount of drug molecules present in a printed spot.

### Crystallization Onset Risk of Different APIs within PVPVA Polymeric Matrix

**Figure 7:**
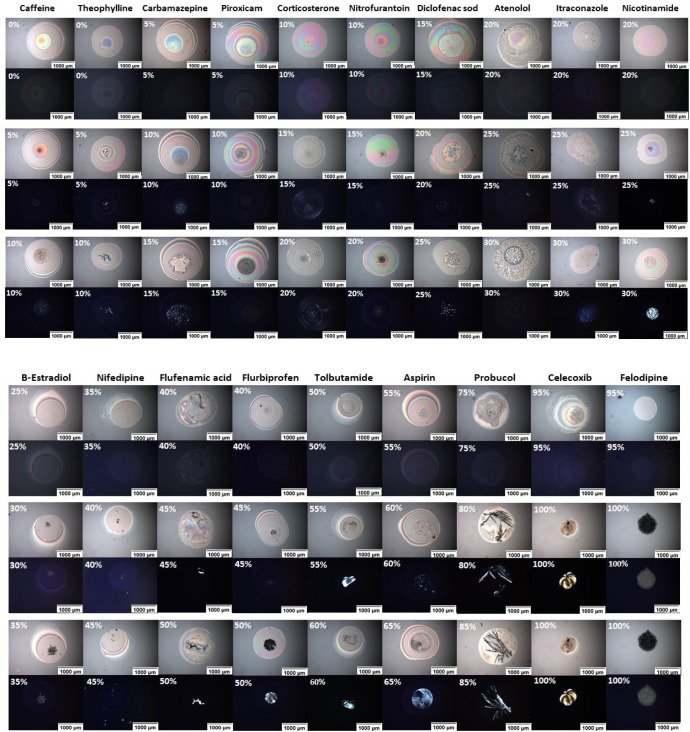
Examples of different APIs/PVPVA printed dispersions. All images are reported without a cross-polarized filter (top part of each section) and with a cross-polarized filter (bottom part). The images were arranged in ascending order according to the displayed drug–polymer ratio. The top section shows the API/polymer loading ratios with no birefringence (no crystallization monitored). In contrast, the middle section shows the API/polymer loading ratios that show the start of birefringence detection (start of crystallization), and the bottom section shows the API/polymer loading ratios that show apparent birefringence detection (crystallization). Three different ratios for API/polymer loading were displayed to compare the assessment of crystallization by POM. All images are reported after storage of the microarrays in accelerated conditions for 6 months (75% relative humidity and 40 °C in stability oven). Final mass: 1000–1100 ng.

The crystallization onset risk of different drugs in PVPVA matrices was thoroughly screened. Figure 7 presents bright-field and cross-polarized images of three different API/polymer loading ratios, first with no birefringence (no crystallization monitored), second with ratios that show the start of birefringence detection (start of crystallization), and finally, ratios that show birefringence detection (crystallization) six months after printing, having been stored at 40 °C/75%RH for that time.

Caffeine and Theophylline, which have a similar chemical structure, appeared to crystallize immediately after solvent evaporation at all drug–polymer ratios. We, therefore, deduce that they had a low crystallization onset risk with the polymer tested here as the polymer could not stabilize either compound in an amorphous state. Conversely, from GFA Class I, Aspirin and Tolbutamide had a high crystallization onset risk with PVPVA, and this drug crystallizes only at high drug loadings in PVPVA (>50% and 55%, respectively). From GFA Class II, Celecoxib and Felodipine showed amorphicity at all ratios with a very high apparent crystallization onset risk. Other different APIs showed variable apparent crystallization onset risks within the polymeric matrices, as shown in Figure 7, as well as different onsets of crystallization, as (Table S2 and Figures S1–S20).

A comparison was made to the literature for Flufenamic acid and Itraconazole, whereby formulations and weight ratios reported in the literature by spin coating and film casting^7,65^ were matched with the presented high-throughput miniaturized method. A crystallization onset risk of 45% (w/w) of Flufenamic acid in the polymeric matrix in the microarray compared to previously published outcomes obtained by spin coating of around 60%.^7^ The printed spots of Itraconazole/PVPVA blends showed a crystallization onset risk at 25% (w/w), compared to results obtained by a film-casting technique that showed that Itraconazole was miscible with PVPVA up to 2:8 (i.e., 20%) (w/w) drug to polymer ratio.^65^ There is, hence, a reasonable agreement between the microarray drug-in-polymer miscibility and previous literature, at least where such comparisons can be meaningfully made under such different experimental conditions. The amount of material needed for analysis and prediction using the microarray approach is perhaps the key benefit of the current study. In reality, for each drug/polymer ratio formulation, a maximum of 3 μg was deposited using the microarray approach against an approximate value of 5–15 mg following a drop-casting methodology.^7,68^

### Drop-Cast Method of Drug/PVPVA Dispersion

**Figure 8:**
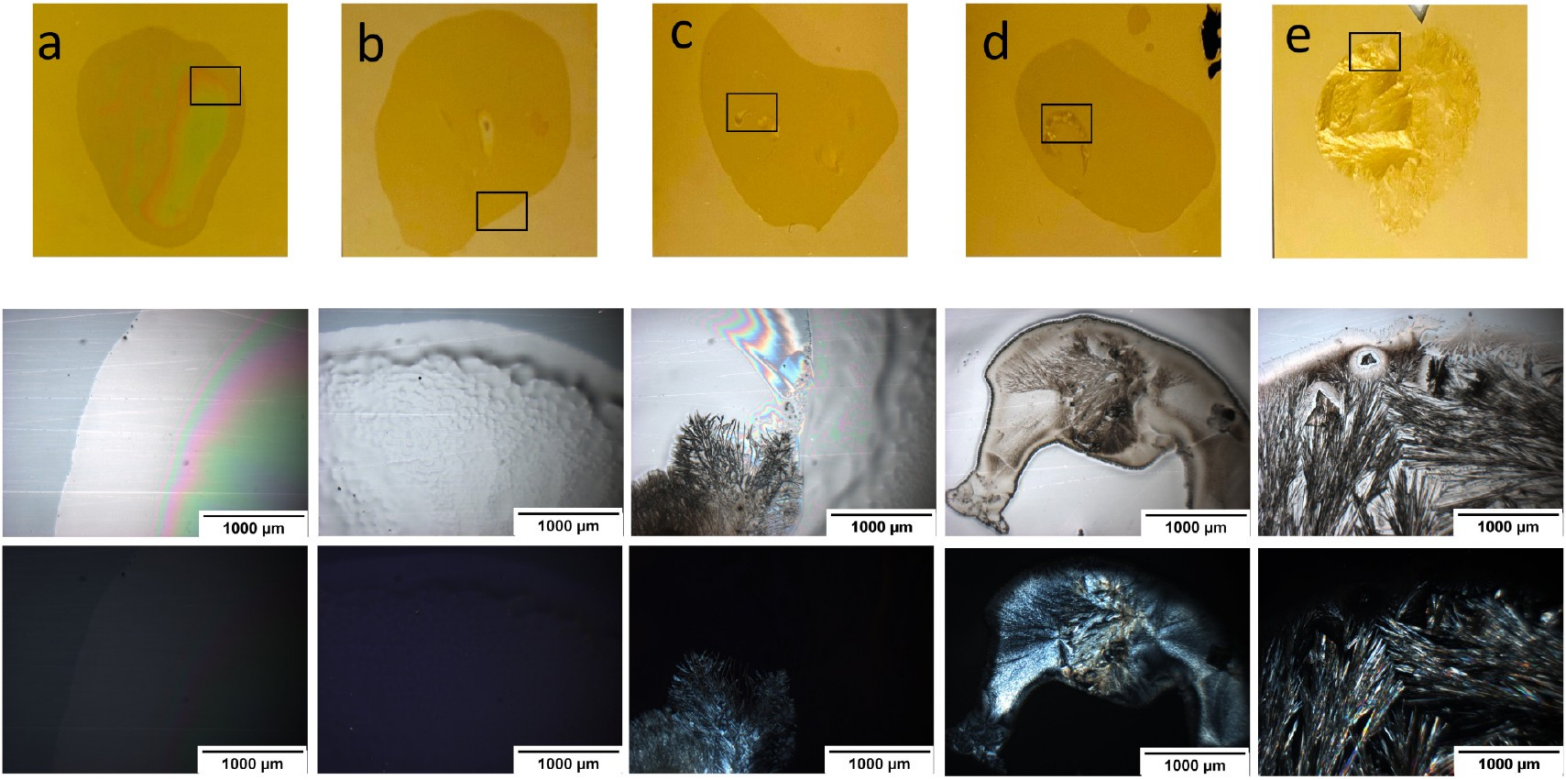
Top row shows the spots by drop cast technique for Flurbiprofen/PVPVA pipetted on gold-coated glass slides as a substrate. a) Pure polymer PVPVA (0%), b) 40% Flurbiprofen/PVPVA, c) 45% Flurbiprofen/PVPVA, d) 50% Flurbiprofen/PVPVA, e) Pure Flurbiprofen. (100%),images resembled a small section with optical (middle row) and cross-polarized (bottom row).

To assess inkjet printing as a manufacturing technique to facilitate predicting the drug–polymer formulations suitable as solid dispersions, three drug/polymer ratios at the crystallization onset risk and below and above this level were selected to be further analyzed at a milligram scale. Figure 8 represents the drop-casted samples in the top row and then bright-field and cross-polarized images of drop-casted three Flurbiprofen-PVPVA loadings and pure Flurbiprofen and PVPVA six months after printing, stored at 40 °C/75%RH, data for other APIs are available (Figures S21–S24).

Drop-cast results were comparable to our microarray results; for example, no evidence of birefringence was found for the pure polymer and the 40% Flurbiprofen/polymer ratio in both employed techniques. This can indicate the predictability of our nanoscale microarray technique to the behavior of APIs on a larger scale. For the 40% drug/polymer ratio, the total deposited mass of the printed spots by 2D inkjet printing as a miniaturized approach was 400 ng drug and 600 ng polymer, while for by drop cast method was 0.8 mg drug and 1.2 mg polymer (Table 3). Furthermore, at ratios of 45% and 50%, as well as the pure Flurbiprofen in the drop-cast samples, PLM observation showed birefringence similar to that in the printed microarray samples.

**Table 3:** Comparing the Printed Volume and Deposited Mass of Two Techniques, the Drop- Cast Method and 2D Inkjet Printing

	Drop-cast technique (Pipetted volume is 200 μL)		2D inkjet printing (500 droplets, droplet size is 200 pL)			
	Deposited mass		Printed volume		Deposited mass	
Formulation	Drug	Polymer	Drug	Polymer	Drug	Polymer
100% Flurbiprofen	2 mg	0	100 nL	0	1000 ng	0
100% Pure PVPVA	2 mg	0	100 nL	0	1000 ng	0
40% Flurbiprofen/PVPVA	0.8 mg	1.2 mg	40 nL	60 nL	400 ng	600 ng
45% Flurbiprofen/PVPVA	0.9 mg	1.1 mg	45 nL	55 nL	450 ng	550 ng
50% Flurbiprofen/PVPVA	1 mg	1 mg	50 nL	50 nL	500 ng	500 ng

Hence, by comparing the results of 2D inkjet printing with the drop-cast method, we found that both methods produced consistent and comparable results for the same drugs and drug/polymer ratios. This agreement confirms that the 2D inkjet printing method is efficient and reliable for screening drug–polymer solid dispersion formulations as the results were not impacted by the different evaporation rates between the two techniques.

Overall, 2D inkjet printing as a miniaturization technique may offer significant efficiency in pharmaceutical formulation screening, with each experiment in the nano microarray format requiring at least 3 orders of magnitude lower amounts of sample than even thin-film screening methods.

Inkjet printing satisfies the needs of a high-throughput-miniaturized screening approach, not only in drug-solid dispersion but in those areas where crystallization needs to be assessed (cocrystallization, for example). These factors include tailored flexibility in terms of materials and solvents adopted,^69^ high degrees of automation, speed of execution,^70^ combined with low error^71^ and wastage.^21^ Even though only a few drugs and polymers were employed in the present study, 400–500 spots can easily fit on a single microscope slide and can be produced at a rate of about 1000 spots per hour. In around 45 min, PLM analysis and taking a record of a single slide can be completed.

While inkjet printing facilitates high-throughput formulation screening, stability testing remains time-intensive due to the necessity of long-term evaluation under harsh conditions (40 °C/75% RH for six months). Shorter durations could provide faster insights, but our extended time frame offers a more cautious estimate of stability. Regarding PLM, its manual and qualitative nature is acknowledged; however, its sensitivity and minimal sample requirements make it a suitable tool for detecting crystallization in high-throughput screening settings.

The availability of large amounts of data related to significant chemical and physical diversity is beneficial to develop models that can predict the stability of solid dispersion (API/polymer blend) depending on the APIs’ and polymer matrix physicochemical properties.

### Development of MLR Model

Multiple linear regression models, one model for each drug/polymer combination, were developed. The selection of parameters for the modeling process was based on consideration of physicochemical properties that are known to influence the behavior, interactions, and characteristics of molecules in pharmaceutical and chemical contexts, as well as the parameters used in other modeling on smaller data sets in the literature.^41,52^

To circumvent the challenge of linear dependency among covariates, they were subjected to an assessment of intercorrelation. The correlation matrix that displays the pairwise linear correlations between the covariates and log stability within PVPVA is provided in Figure S25. When the correlation coefficient exceeded a threshold of 0.9, indicating a high level of multicollinearity, additional iterations of the covariate selection were performed.

In this study, multiple linear regression (MLR) models were developed for each drug–polymer combination to identify variables associated with the logarithmic stability of drugs across a range of polymer percentages. MLR was chosen because it offers a simple, interpretable final model that requires no special expertise and is easy to interpret from a chemical perspective. It handles multiple variables, allowing us to quantify the impact of each molecular descriptor on the stability while maintaining statistical robustness. Its straightforward implementation and validation make it a reliable approach and provide a baseline for exploring more advanced models in future studies.

Analysis of the data set, which included drug–polymer loadings from 5 to 100% in 5% increments, revealed varying frequencies of physicochemical properties within the MLR models. Notably, properties such as hydrogen bond acceptors (HBA), the number of heteroatoms (nHet), melting point (mp), and oxygen content (nO) were frequently included in the models, suggesting their significant impact on drug stability. Conversely, other properties like atom count (nAtom) and van der Waals surface area (vdW.SA) appeared less often, indicating a potentially lower role in stability. These findings highlight the importance of specific physicochemical properties in influencing the stability of drugs in PVPVA matrices.

Using the data collected for all of the compositions, one equation that best represents our model was developed. The representative equation for the MLR model is expressed in eq 1, while the representative equations for each drug/polymer loading and the adjusted R^2^ values are detailed in Table S3.
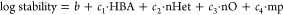
1where *b* is the intercept, HBA represents the number of hydrogen bond acceptors, nHet is the number of heteroatoms, nO is the number of oxygen atoms, mp is the melting point, and *c*_1_–*c*_4_) are the coefficients for each variable.

The fitted R^2^ extracted from the multiple linear regression models for the PVPVA data set, using all drug/polymer loadings (5–100% with 5% increment) for the various employed APIs, is shown in Figure S26. The adjusted R-squared values offer valuable insights into the model’s predictability across different drug/polymer loadings. At the outset, with lower drug loadings (around 5%), the model has an adjusted R-squared value of 0.19, indicating that only 19% of the variability in the log stability is captured by the model. At higher drug loadings, the adjusted R^2^ values exhibit a consistent increase to a value of 0.69 at 95% loading. As the loading of drug molecules increases, the model gains access to a greater number of molecular interactions, such as hydrogen bonding, van der Waals forces, and steric effects that significantly influence the stability and behavior of the amorphous solid dispersions. This enhanced understanding enables the model to reflect the system’s complexities more accurately, ultimately better fitting the experimental outcomes observed in our study. When the drug loading exceeds a certain threshold, specifically, the maximum solubility of the drug in the polymer, two significant phenomena may occur, local supersaturation and subsequent nucleation. Local supersaturation can lead to the formation of small crystals, even in areas where the bulk system remains homogeneous. This shift can trigger processes of crystallization, where nuclei form and grow, potentially compromising the physical stability of the amorphous solid dispersion. As these processes occur, they introduce additional complexities that may impact the model’s predictive accuracy. The model’s predictive capability is shown to be high, particularly for high drug/polymer loadings. The model more accurately represents these formulations. Experimental data further corroborate these findings. It is observed that formulations with higher drug loadings display an increased propensity for recrystallization. The fluctuation in the coefficients (*c*_1_, *c*_2_, *c*_3_, and *c*_4_) for the key model parameters (HBA, nHet, nO, and mp) as a function of drug/polymer loading (0–100%) is shown in Figures S27–S30. That enables an understanding of how different descriptors contribute to log stability at various drug loadings.

To evaluate whether assigning a nominal stability of 500 days to noncrystallized drug formulations impacted the accuracy of model outputs, stability was tested within the model at extended nominal durations of 1000, 10,000, and 100,000 days. The comparison revealed that the model consistently selected the same key parameters: hydrogen bond acceptors (HBA), number of oxygen atoms (nO), number of heteroatoms (nHet), and melting point (mp), regardless of the assigned stability duration for samples that had shown no crystallization. Therefore, using 500 days as a nominal stability for noncrystallized drugs is a reasonable and practical choice within the tested range.

### Comparative Analysis of Predicted versus Measured Stability across Various Drug Formulations Using LOOCV

**Figure 9:**
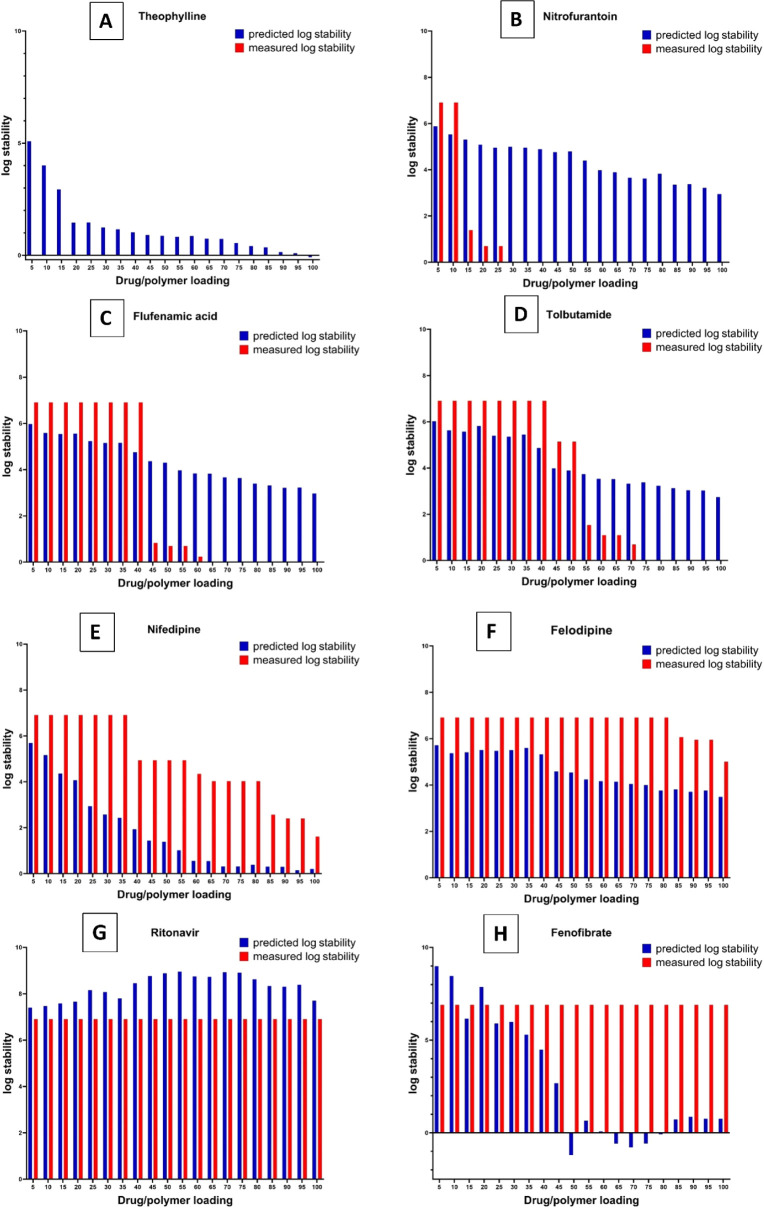
Comparison of predicted log stability, determined using the LOOCV based on the multiple linear regression model, versus measured log stability from experimental microarray data for Theophylline (A), Nitrofurantoin (B), Flufenamic Acid (C), Tolbutamide (D), Nifedipine (E), Felodipine (F), Ritonavir (G), and Fenofibrate (H) across a range of drug loadings within the PVPVA polymeric matrix. The blue bars indicate the predicted stability, while the red bars show the measured stability, allowing for an evaluation of the predictive model’s accuracy at different drug loadings.

The LOOCV approach was utilized to assess the accuracy and reliability of our model.^72^ This approach involves sequentially removing the data for a single drug and applying the model to the remaining data set. The data of the omitted drug were then used to validate the model. This process was repeated for each drug in the data set, ensuring a robust evaluation of the model’s performance. Figure 9 presents a comprehensive comparison of the log stability predicted using multiple linear regression and LOOCV versus the experimentally measured log stability for eight distinct drugs that showed different crystallization behaviors experimentally within the PVPVA polymeric matrix. The data for the other drugs are provided in Figures S32 and S35.

In this context, the stability profiles of various drug–polymer dispersions are evaluated through a comparison of the predicted and measured log stability values across different drug loadings. Theophylline’s measured stability consistently shows immediate crystallization across all drug/polymer loadings, contrasting with the predicted stability, which initially starts high but declines rapidly beyond 20% loading. This discrepancy suggests limitations in the prediction model’s reliability for Theophylline and Caffeine, possibly due to the immediate crystallization behavior at all the drug/polymer loadings in both APIs making similar entries of the stability data at all loadings. Nitrofurantoin exhibits high measured stability at lower loadings, declining sharply to zero at higher loadings, which is partially reflected in the predicted stability trend. Despite discrepancies in magnitude, the model captures the overall trend of decreasing stability with increasing drug/polymer ratios. Moreover, Flufenamic acid displays stable measured log stability at lower loadings, reducing abruptly to zero at loadings above 60% due to crystallization, whereas the predicted stability decreases more gradually. Similarly, Tolbutamide shows a decline in measured stability as loading increases, with both predicted and measured data indicating a stable environment at lower loadings and reduced stability at higher loadings above 50%. Nifedipine’s measured stability decreases with increasing drug/polymer loading, reflected in the predicted trend that mirrors this decline. Felodipine exhibits consistently high measured stability across lower loadings, with a notable decrease at higher loadings, a trend that is somewhat mirrored in the predicted stability, albeit with less pronounced drops. In contrast, Ritonavir demonstrates consistently high measured stability across all loadings, whereas the predicted stability, while generally elevated, exhibits some fluctuations and tends to overestimate the stability compared with measured data. Fenofibrate’s stability remains high across all loadings in measured data, but the predicted stability shows fluctuations and decreases notably at higher loadings, indicating a divergence between model predictions and experimental results beyond 45% loading. Overall, while the prediction model captures general trends in stability for most formulations, discrepancies exist, particularly in cases of abrupt stability changes due to crystallization at higher loadings. This suggests areas where the model may benefit from further development with larger data sets to enhance its predictive accuracy, especially in formulations prone to sudden stability shifts.

Further analysis of the eight representative drugs within the PVPVA polymeric matrix has provided key insights into the prediction model’s capabilities and limitations. Several consistent observations emerged for multiple drugs. First, the observation of uniformly high stability across multiple drug/polymer loadings for a range of drugs, including Ritonavir, Fenofibrate, Aprepitant, Orlistat, Felodipine, Probucol, and Celecoxib, highlights a specific trend that might have impacted the predictive accuracy of our model due to the limited variability in the data set. This is a particular issue in scenarios where no crystallization was detected.

Additionally, in cases like Flufenamic acid, Tolbutamide, Nifedipine, Nitrofurantoin, Atenolol, Nicotinamide, Diclofenac sodium, Flurbiprofen, Aspirin, Carbamazepine, Estradiol, Piroxicam, and Corticosterone, the predictive model captures the overarching trend of stability across various drug–polymer loadings, even if it did not precisely predict the exact measured stability values. This improved predictive capability can be primarily attributed to the diverse range of stability values observed across drug–polymer loadings.

In contrast, the observed immediate crystallization of drugs such as Theophylline and Caffeine across all drug loadings presented significant challenges for the predictive accuracy of our model, again due to lack of diversity within this cluster.

The comprehensive analysis of data, encompassing all drugs formulated within PVPVA and covering a broad spectrum of drug loadings and physicochemical properties, helped elucidate the complex nature of stability in solid dispersions. Despite the inherent complexity and variability of the models, this analysis allowed for the identification of overarching trends and significant correlations that can influence stability. It can be concluded that several parameters correlate with increased stability of the solid dispersions, including notably HBA, nHet, nO, and mp.

The analysis in our model indicates that the number of HBA in a drug molecule plays a significant role in the stability of solid dispersions with PVPVA. Several studies have investigated the significance of hydrogen bonding interactions in influencing the thermodynamic activity and dynamic characteristics of drugs and polymers in their mixtures.^73,74^ Xiang and Anderson studied the molecular structure of ibuprofen (IBP) in various polymeric mixtures by using molecular dynamics simulations. They found that hydrogen bonding between IBP and polyvinyl pyrrolidone (PVP) in amorphous dispersions competes with IBP-IBP hydrogen bonding, with this disruption being more pronounced in IBP-PVP dispersions compared to those with PVPVA and PVA.^75^ Another study examined the impact of intermolecular hydrogen bonds between drugs and the PVPVA polymer on the stability of ASDs over 60 days. It found that hydrogen bonds have varying effects, with the most significant impact on stability in ASDs with drugs that have a moderate crystallization tendency, also influencing drug loading capacity.^76^ Kestur et al. studied how different polymers affect the crystal growth rates of bifonazole and nimesulide, focusing on hydrogen bonding interactions. They found that the effectiveness of a polymer in inhibiting crystallization depends on the specific chemistry of the drug and the availability of hydrogen bonding groups in the polymer.^64^

The type of polymer significantly affects the formation and stability of solid dispersions due to its properties, such as molecular weight, solubility, Tg, and moisture uptake. Selecting a polymer with higher Tg or strong drug–polymer interactions can enhance stability by reducing crystallization risk.^77^ In our study, we focused on PVPVA as the primary polymer matrix, examining its interactions with APIs of varying physicochemical properties to assess its influence on stability and crystallization behavior.

Numerous studies have also discussed the significant impact of hydrogen bonding on the miscibility of drugs within a polymer matrix. However, the stability of solid dispersions concerning hydrogen bonding has not been addressed in these studies. Fridgeirsdottir et al. developed six statistical MLR-based models using one drug/polymer loading (10% w/w) of 10 APIs with three polymers through two manufacturing methods. They investigated the correlation of hydrogen bonding in the drug molecules with the stability of ASD, as increased stability correlates with a decreased number of HBDs in the drug molecule.^52^

From our developed model, the increase in the number of hydrogen bond acceptors (HBA) on the drug molecule (while keeping nHet, nO, and mp fixed) was found to negatively correlate with stability, particularly at mid to high drug loadings when formulated with PVPVA. The stability is influenced by the compatibility and arrangement of hydrogen bond donors (HBD) and HBA between the drug and polymer. At higher drug loadings, the likelihood of drug–drug or drug–water bonding increases, which can lead to crystallization and reduced stability, highlighting the significant role of hydrogen bonding interactions.

Specifically, when using PVPVA, a polymer that contains only HBA groups, the negative correlation may be attributed to electrostatic repulsion between the drug’s HBA groups and the polymer’s HBA groups (while keeping nHet, nO, and mp fixed). This repulsion can disrupt the uniform distribution of the drug, leading to potential drug clustering and phase separation. Such segregation may promote the formation of crystalline regions, further diminishing the stability of the ASD. This novel finding has been identified and thoroughly addressed in our study.

The number of heteroatoms (nHet) in a drug molecule strongly correlates with the stability of ASD formulations (while keeping HBA, nO, and mp fixed), particularly at mid to high drug loadings. The coefficient here is positive for all systems at all loadings, which suggests that an increase in nHet stabilizes the ASD. Due to their electronegativity, heteroatoms like oxygen and nitrogen create regions of partial negative charge that interact favorably with the polymer matrix, stabilizing the amorphous form and reducing the likelihood of nucleation and crystallization. Additionally, these heteroatoms enhance hydrogen bonding and other intermolecular forces, helping to maintain the disordered state necessary for stability. This effect is due to enhanced drug–polymer intermolecular interactions such as dipole–dipole for drugs with a high number of heteroatoms.^78^ The nHet can affect the stability by decreasing the chance of molecular alignment into a crystalline structure. Mahlin et al.’s model using PLS-DA further supports the positive impact of electronegative heteroatoms on a compound’s ability to form and maintain an amorphous state, with characteristics such as branched carbon skeletons and molecular asymmetry also contributing to enhanced stability.^79^ Although there is a strong correlation between HBA and nHet, this does not affect the model predictions, but care must be taken with interpretation of the estimated coefficients.

An increased number of oxygen atoms (nO) was associated with enhanced stability of the solid dispersions, particularly at mid to higher drug loadings. This suggests that compounds with more oxygen atoms have a tendency to form more stable interactions with the polymer matrix (PVPVA). Oxygen atoms can participate in hydrogen bonding, which may contribute to this enhanced stability. The moderate correlation between nO and HBA (0.6) indicates that while related, they represent distinct structural factors that both play important roles in the overall stability of solid dispersions.

Melting point exhibits a negative effect on the stability of solid dispersions. The coefficient here is negative for all systems at all loadings, suggesting that higher melting point drugs have a stronger tendency to phase separate from the drug–polymer ASD than lower melting point drugs. This suggests that lower melting point drugs tend to be more stable as ASD formulations within the polymeric matrix than drugs with higher melting points, particularly for typical pharmaceutical loadings (5–25%).

Our study suggests that both repulsive and attractive interactions should be considered when formulating an ASD. While various previous studies have explored attractive interactions in the formulation of ASDs,^52,64,80^ our research highlights the significance of considering both repulsive and attractive interactions to enhance the stability and performance of these systems.

## Conclusions

This study has demonstrated the potential of using printed microarrays with picoliter quantities of various APIs for preformulation and as a promising approach for early stage solid-form screening. An extensive library of 437 drug/polymer loadings (1311 loadings in triplicate) allowed for detecting and comparing crystallization onset risks of drugs within the PVPVA polymeric matrix. Results from the 2D printed arrays were consistent with drop cast techniques, confirming the reliability of our approach when compared to more “bulk” approaches. The number of formulations examined in our study surpasses those in previous studies while using minimal material quantities. The ability to fit 400–500 spots on a single microscope slide and produce about 1000 spots per hour enhances the efficiency. The 2D inkjet printing technique offers significant advantages in material conservation, rapid sample preparation, and analysis, meeting the requirements for high-throughput miniaturized screening, despite limitations related to ink viscosity and material compatibility. The results reported herein apply to the APIs investigated in combination with PVPVA. Further work would be required to extrapolate the findings to different polymers, as polymer selection plays a crucial role in influencing miscibility, stability, and crystallization behavior.

Statistical modeling using MLR was employed to identify key physicochemical properties correlated with the stability of solid dispersions. By constructing models based on an extensive data set of various APIs within PVPVA polymeric matrices, we identified critical variables such as the number of hydrogen bond acceptors, heteroatoms, oxygen atoms, and melting points influencing stability. Specifically, a decreased number of hydrogen bond acceptors and a lower melting point were associated with higher stability, while an increased number of heteroatoms and oxygen atoms also contributed positively to stability. This study highlights the importance of minimizing repulsive drug–polymer interactions to yield a stable ASD. The MLR models demonstrated strong predictive capabilities, as validated by leave-one-out cross-validation, which ensured that the predicted log stability closely matched the experimental outcomes, thereby reinforcing the credibility of our findings.

The promising results obtained in this study indicate a significant potential for applying knowledge-based computational models in the field of pharmaceutical formulation development. The models developed through our research could serve as valuable resources for guiding the early stages of formulation design.

Future research might expand the data set by incorporating a more comprehensive range of APIs with various heteroatom compositions and other properties to develop different models, as while the data set used in this study encompasses a wide range of APIs in terms of size and shape, it may be somewhat biased toward oxygen- and nitrogen-rich compounds. Additionally, expanding to other solvent systems could be explored, while noting the limitation of droplet formation on the substrates and generating reliable microarrays. Future studies may extend to a broader range of polymers, exploring their role in enhancing or diminishing stability in drug–polymer systems and providing a more comprehensive perspective on formulation design. While the molecular descriptors selected in this study effectively capture key aspects of ASD physical stability, ASD stability can be influenced by additional factors such as environmental conditions and polymer characteristics. Future work could incorporate descriptors related to molecular dynamics and environmental sensitivity, such as Tg and hygroscopicity, to refine the model and enhance understanding of ASD behavior under varying conditions. Future studies could involve the development of a classifier model to identify parameters specific to systems that crystallized versus those that did not or took much longer to crystallize. This approach would provide valuable insights into the factors influencing crystallization behavior. External validation of the models and exploring advanced techniques to improve the understanding of solid dispersion stability could be included. Nonetheless, this study represents a significant step forward in understanding API-polymer interactions and their role in the stability of solid dispersions. Integrating machine learning could enhance predictive accuracy, making the models more reliable for pharmaceutical development. This study underscores the potential of computational models to improve drug formulation and efficacy.

## Supporting Information Available

Detailed data regarding crystallization onset and behavior of the various APIs within the PVPVA polymeric matrix, along with raw data for detecting the appearance of birefringence. Drop-cast images for additional APIs are provided, along with detailed comparisons to literature and stability data for other drugs. Detailed steps involved in developing the model, including a correlation matrix of covariates and log stability, equations for various drug/polymer loadings, and their corresponding adjusted R^2^ values. Comparisons of the predicted versus experimentally measured log stability for the other APIs are provided (PDF)
