# Supplementary material for: High-Throughput Microarray Approaches for Predicting the Stability of Drug–Polymer Solid Dispersions
Source: Mol Pharm. 2024 Dec 21;22(1):343–62. doi: 10.1021/acs.molpharmaceut.4c00955 (PMC11707727; doi:10.1021/acs.molpharmaceut.4c00955)
Supplement: Supplementary file 1 — mp4c00955_si_001.pdf [file mp4c00955_si_001.pdf]

High-Throughput Microarray Approaches For Predicting The Stability Of Drug-Polymer Solid Dispersions

Noha F. Ghazi, [a*, d] Jonathan C. Burley, [a] Ian L. Dryden, [b] and Clive J. Roberts [a, c]

[a] School of Pharmacy, University of Nottingham, University Park, Nottingham, NG7 2RD, United Kingdom

[b] Department of Statistics, University of South Carolina, Columbia, SC 29208, United States

[c] School of Life Sciences, University of Nottingham, University Park, Nottingham, NG7 2UH, United Kingdom

[d] Department of Pharmaceutics, Faculty of Pharmacy, Mansoura University, Mansoura, 35516, Egypt

* Email: dr.noha.fawzy@gmail.com, noha.ghazi@nottingham.ac.uk

Supporting Information (SI)

1. Stability data of printed microarrays of APIs within the PVPVA matrix

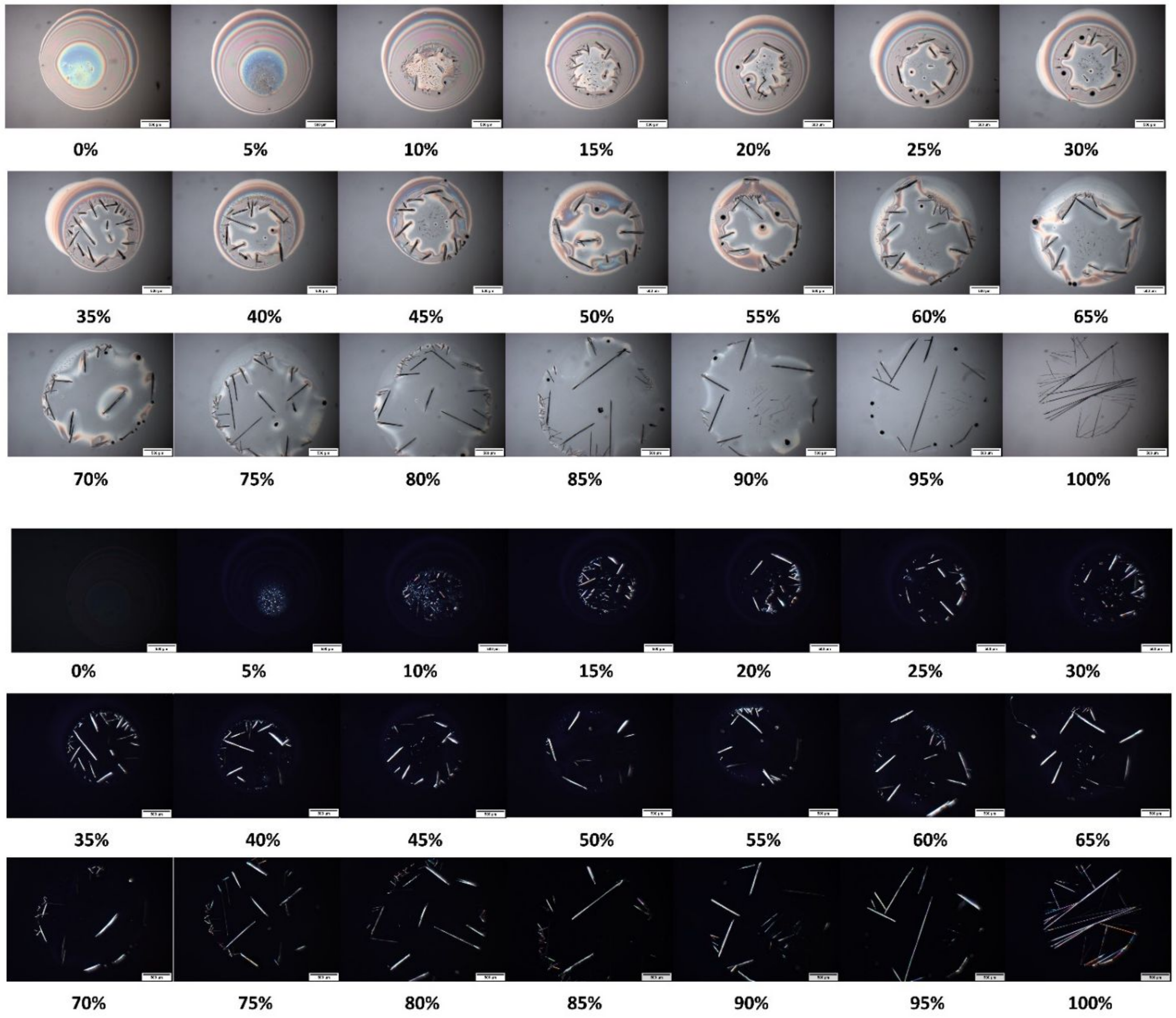

Figure S1. Microarray of Caffeine/PVPVA depicted by PLM. All the spots are reported with the bright field in the upper part of the figure and cross-polarised filters in the lower part of the figure. The images show all different ratios of Caffeine/PVPVA starting from 0% to 100% with a 5% Caffeine increment. The final mass of individual spots is 1000-1100 ng. The scale bar represents 500μm.

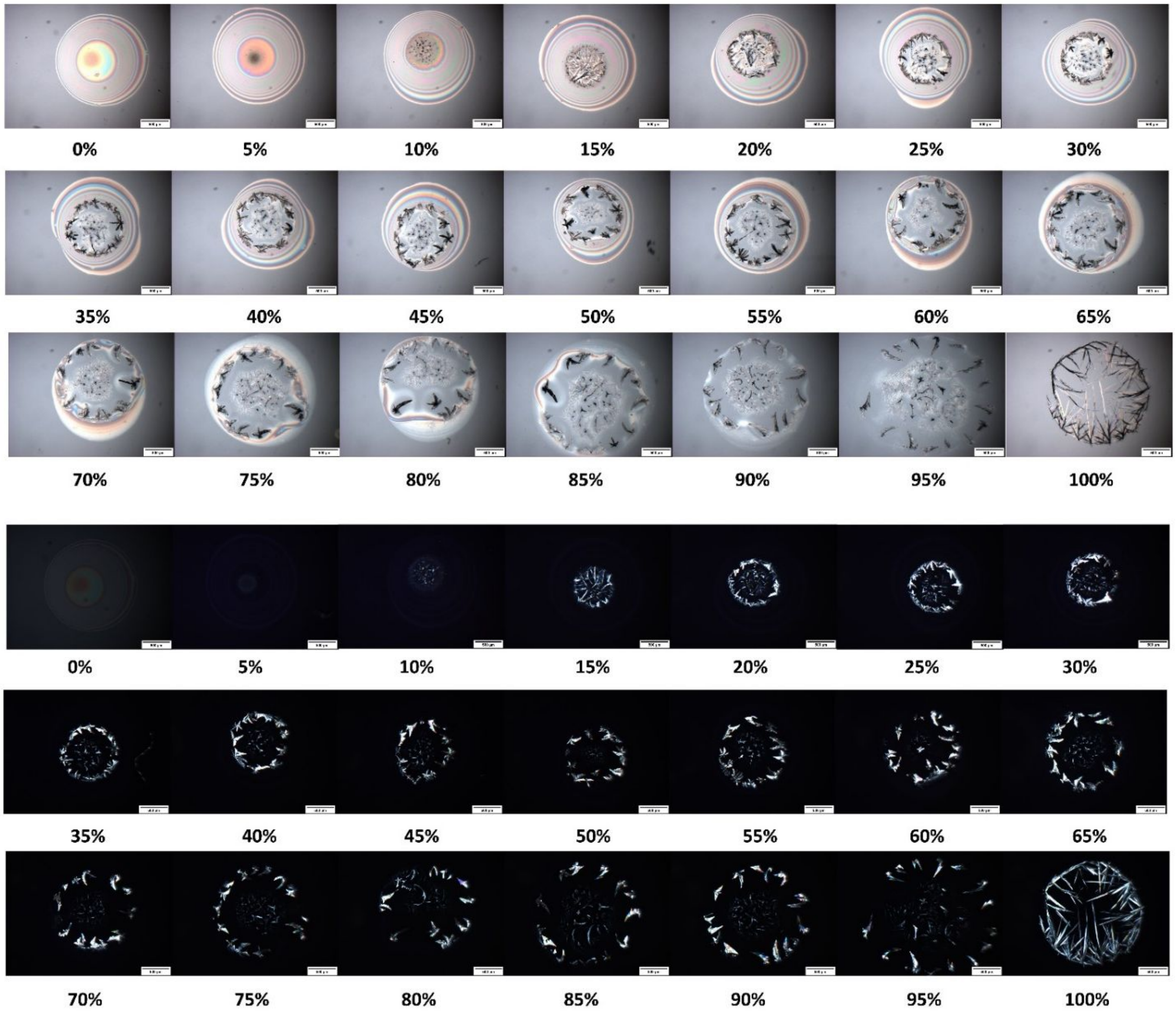

Figure S2. Microarray of Theophylline/PVPVA depicted by PLM. All the spots are reported with the bright field in the upper part of the figure and cross-polarised filters in the lower part of the figure. The images show all different ratios of Theophylline/PVPVA starting from 0% to 100% with a 5% Theophylline increment. The final mass of individual spots is 1000-1100 ng. The scale bar represents 500μm.

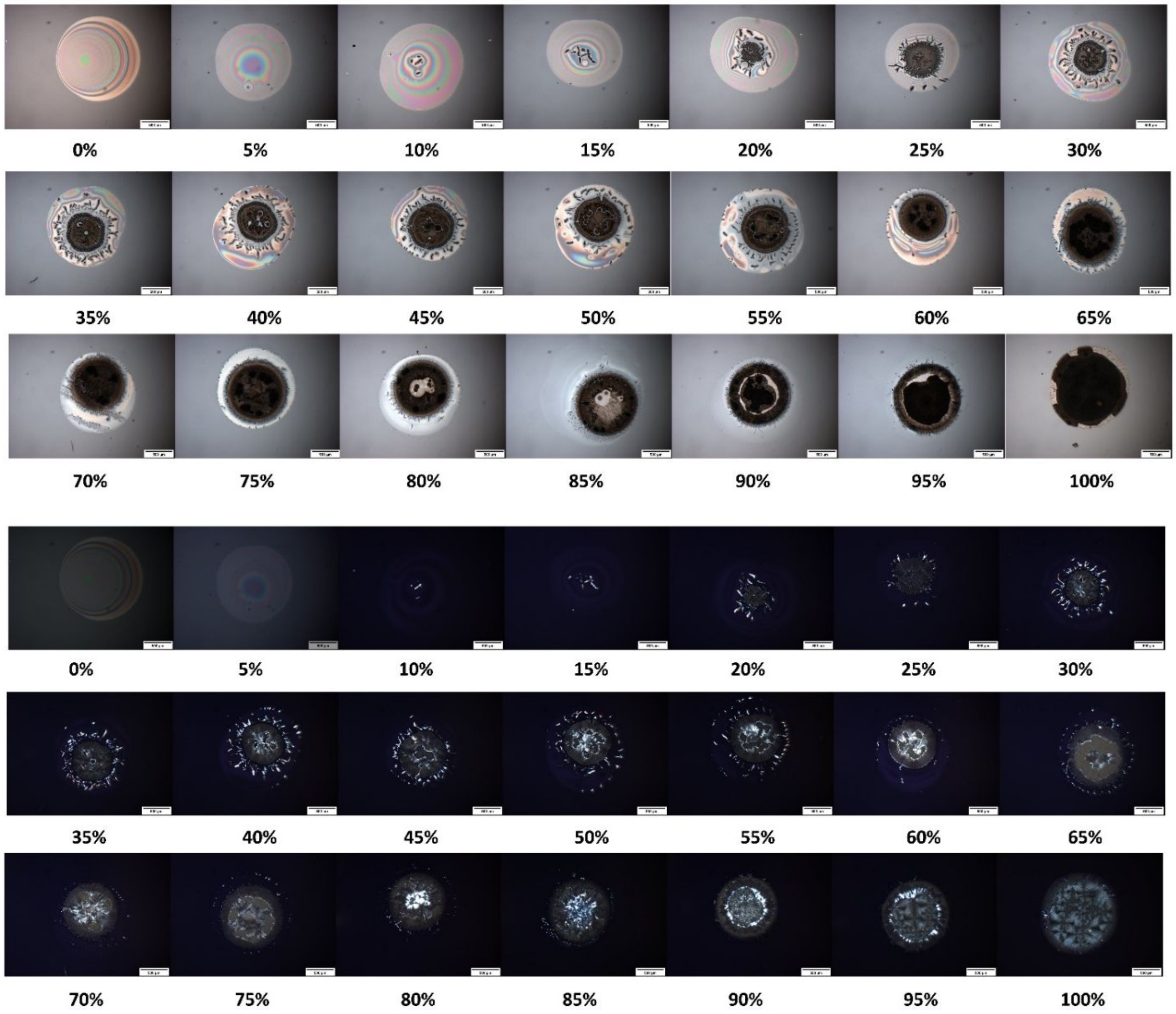

Figure S3. Microarray of Carbamazepine/PVPVA depicted by PLM. All the spots are reported with the bright field in the upper part of the figure and cross-polarised filters in the lower part of the figure. The images show all different ratios of Carbamazepine/PVPVA starting from 0% to 100% with a 5% Carbamazepine increment. The final mass of individual spots is 1000-1100 ng. The scale bar represents 500μm.

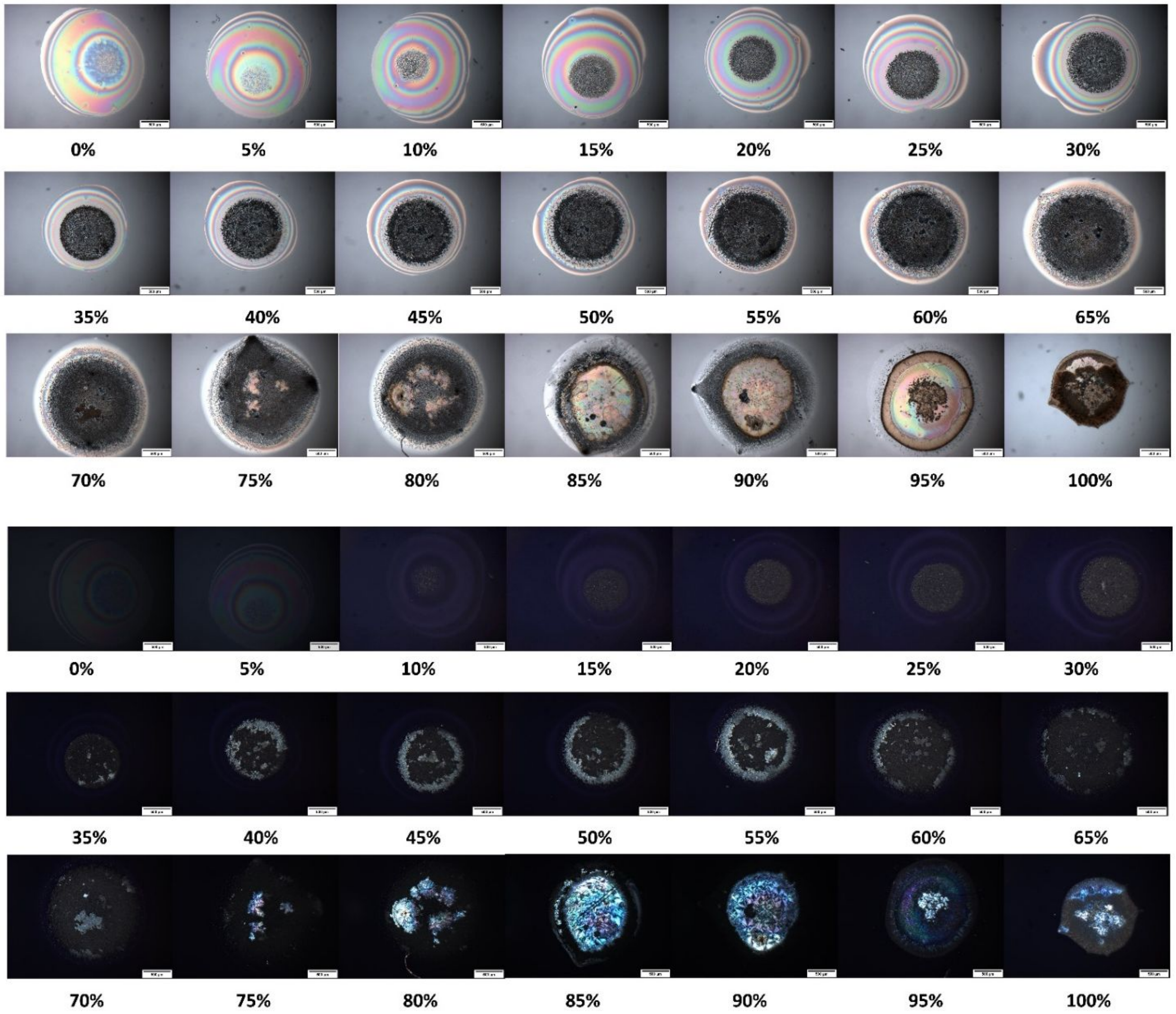

Figure S4. Microarray of Piroxicam/PVPVA depicted by PLM. All the spots are reported with the bright field in the upper part of the figure and cross-polarised filters in the lower part of the figure. The images show all different ratios of Piroxicam/PVPVA starting from 0% to 100% with a 5% Piroxicam increment. The final mass of individual spots is 1000-1100 ng. The scale bar represents 500µm.

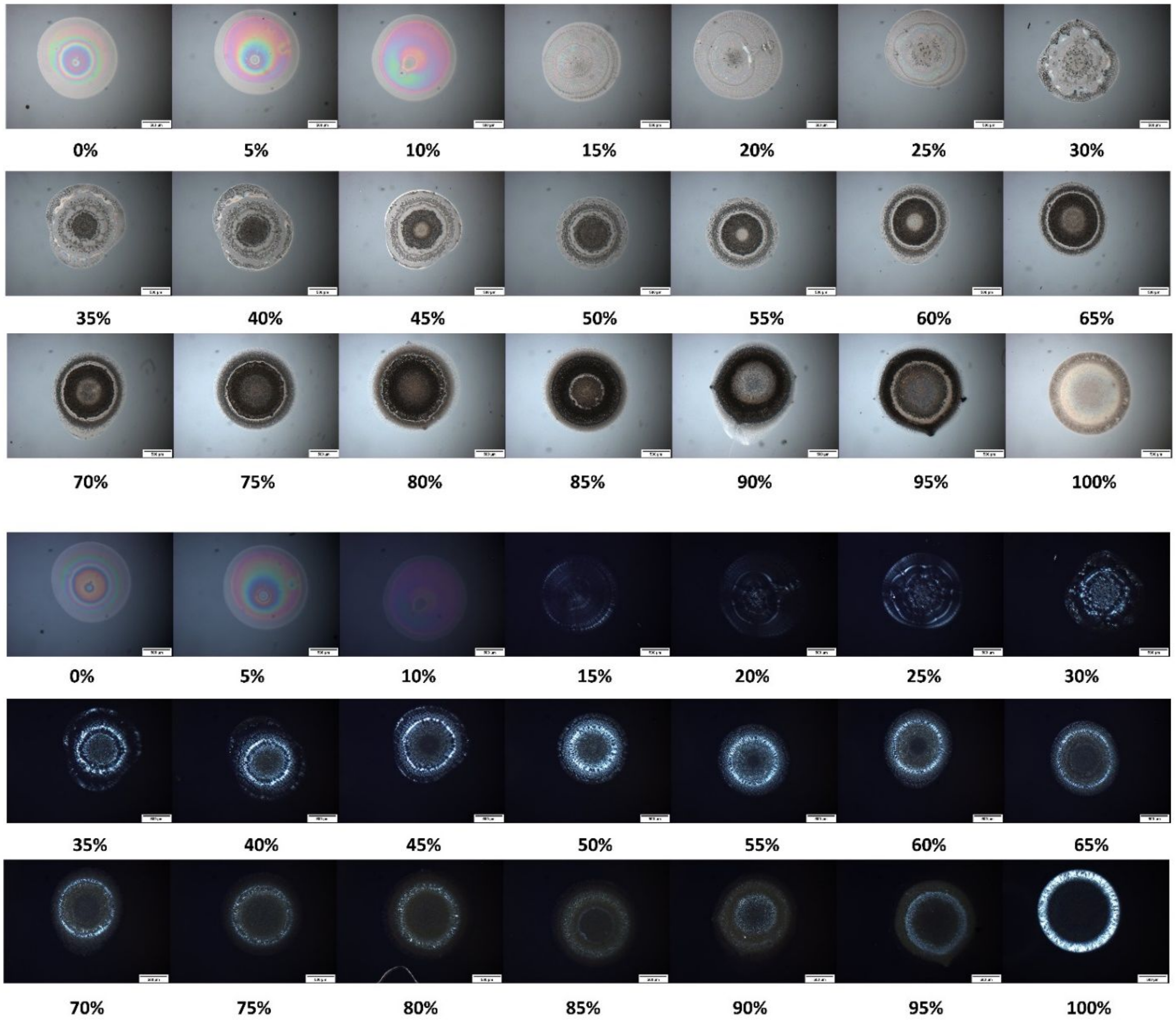

Figure S5. Microarray of Corticosterone/PVPVA depicted by PLM. All the spots are reported with the bright field in the upper part of the figure and cross-polarised filters in the lower part of the figure. The images show all different ratios of Corticosterone/PVPVA starting from 0% to 100% with a 5% Corticosterone increment. The final mass of individual spots is 1000-1100 ng. The scale bar represents 500μm.

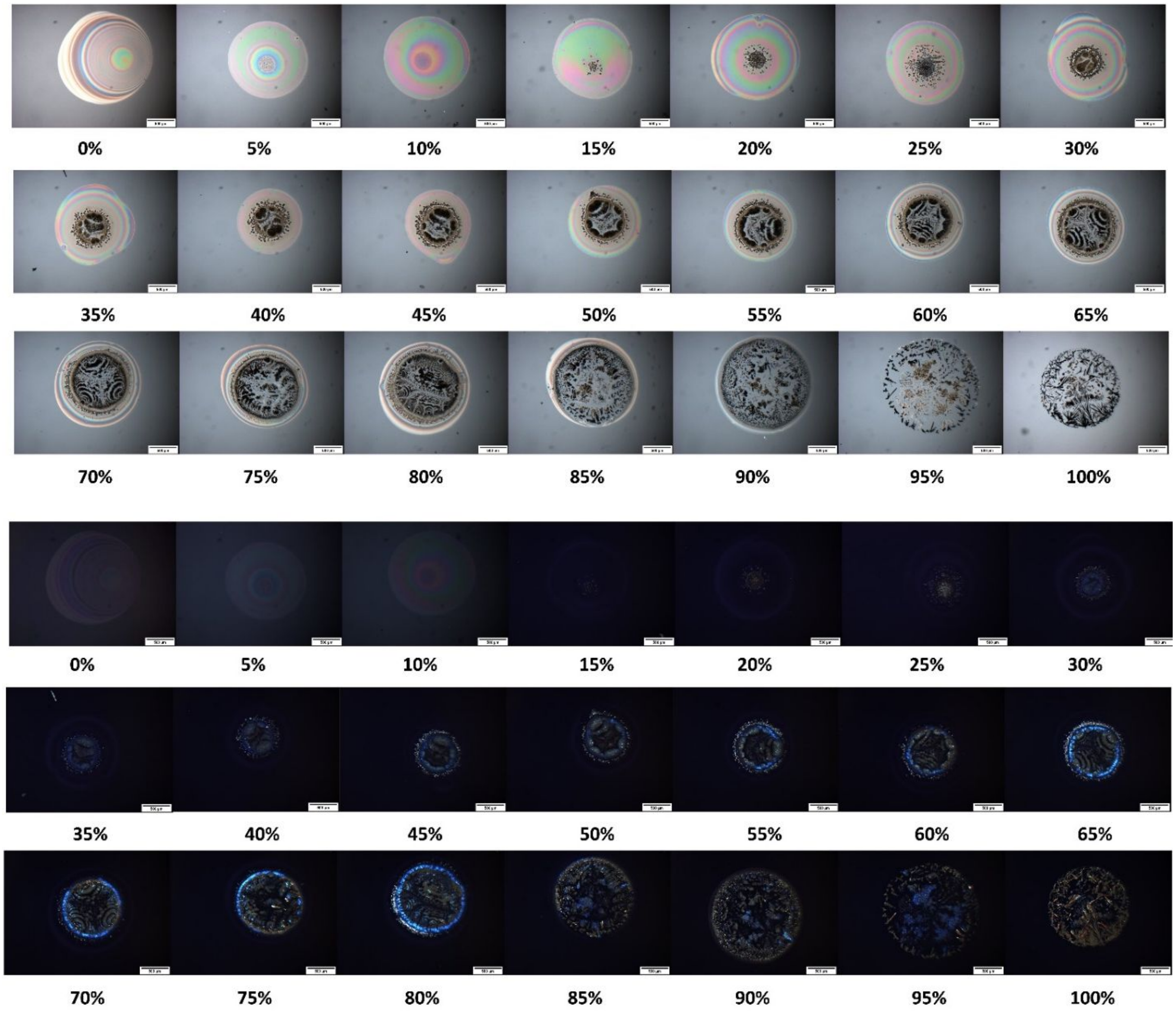

Figure S6. Microarray of Nitrofurantoin/PVPVA depicted by PLM. All the spots are reported with the bright field in the upper part of the figure and cross-polarised filters in the lower part of the figure. The images show all different ratios of Nitrofurantoin/PVPVA starting from 0% to 100% with a 5% Nitrofurantoin increment. The final mass of individual spots is 1000-1100 ng. The scale bar represents 500μm.

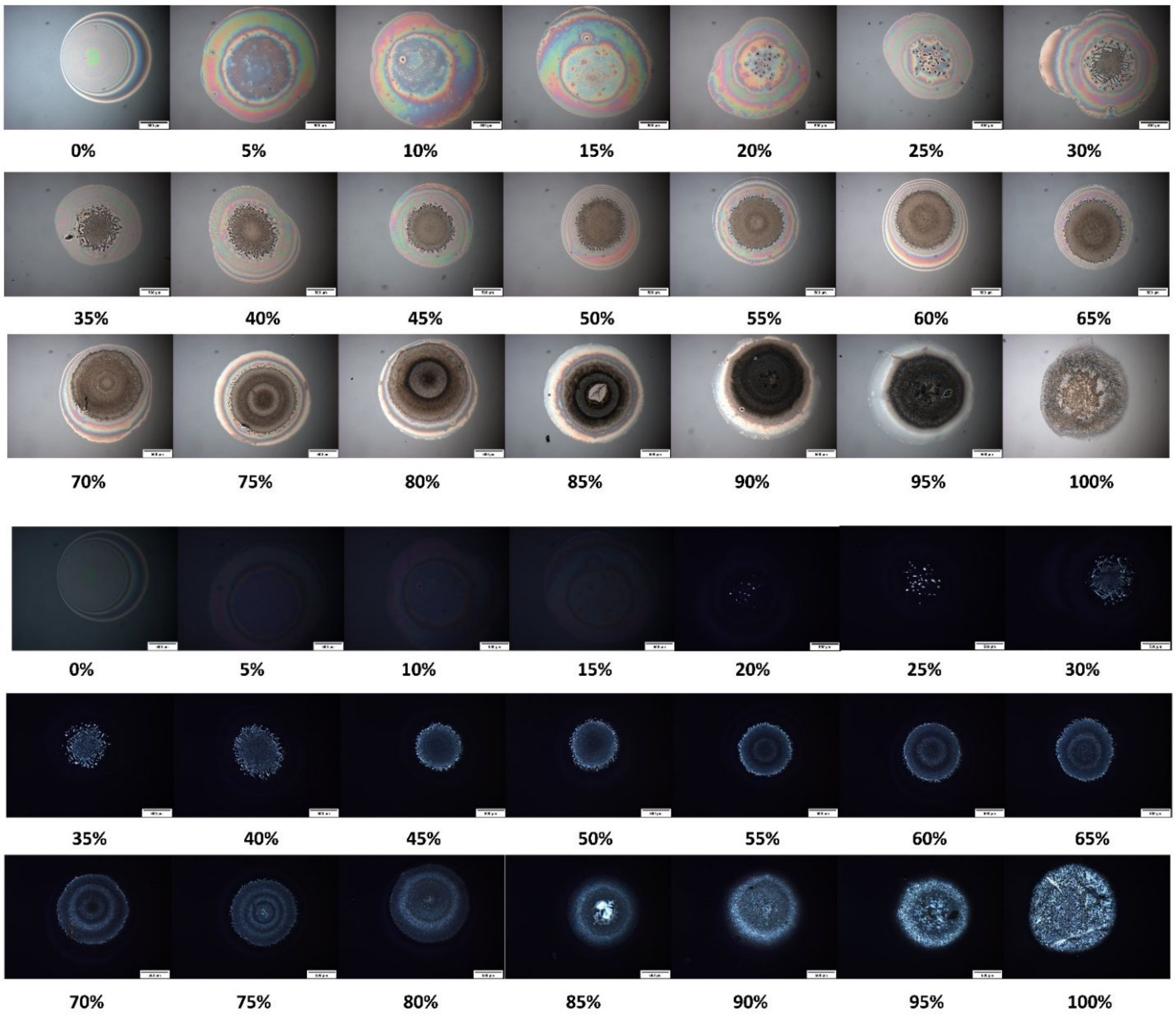

Figure S7. Microarray of Diclofenac sodium/PVPVA depicted by PLM. All the spots are reported with the bright field in the upper part of the figure and cross-polarised filters in the lower part of the figure. The images show all different ratios of Diclofenac sodium/PVPVA starting from 0% to 100% with a 5% Diclofenac sodium increment. The final mass of individual spots is 1000-1100 ng. The scale bar represents 500µm.

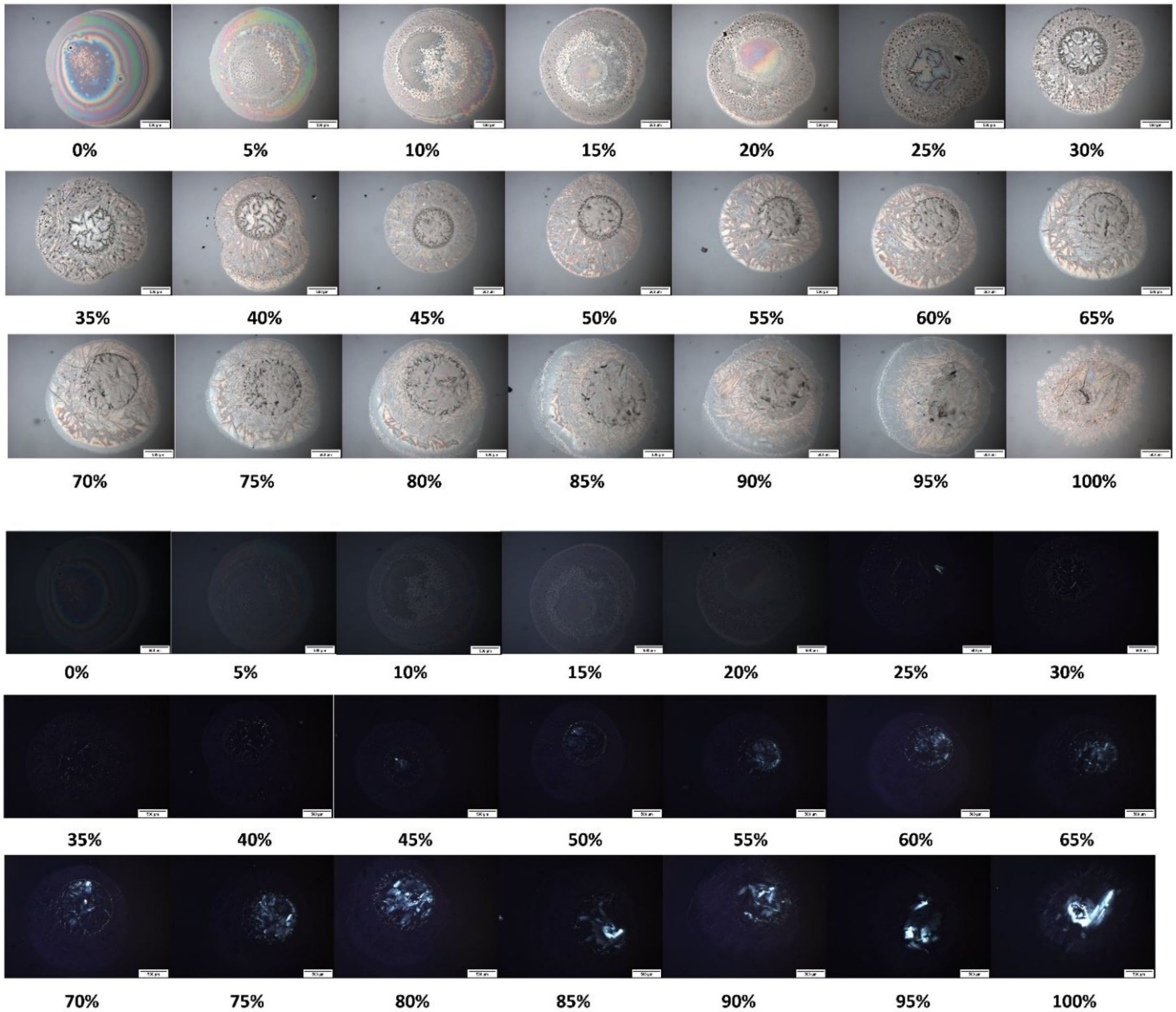

Figure S8. Microarray of Atenolol/PVPVA depicted by PLM. All the spots are reported with the bright field in the upper part of the figure and cross-polarised filters in the lower part of the figure. The images show all ratios of Atenolol/PVPVA, starting from 0% to 100% with a 5% Atenolol increment. The final mass of individual spots is 1000-1100 ng. The scale bar represents 500µm.

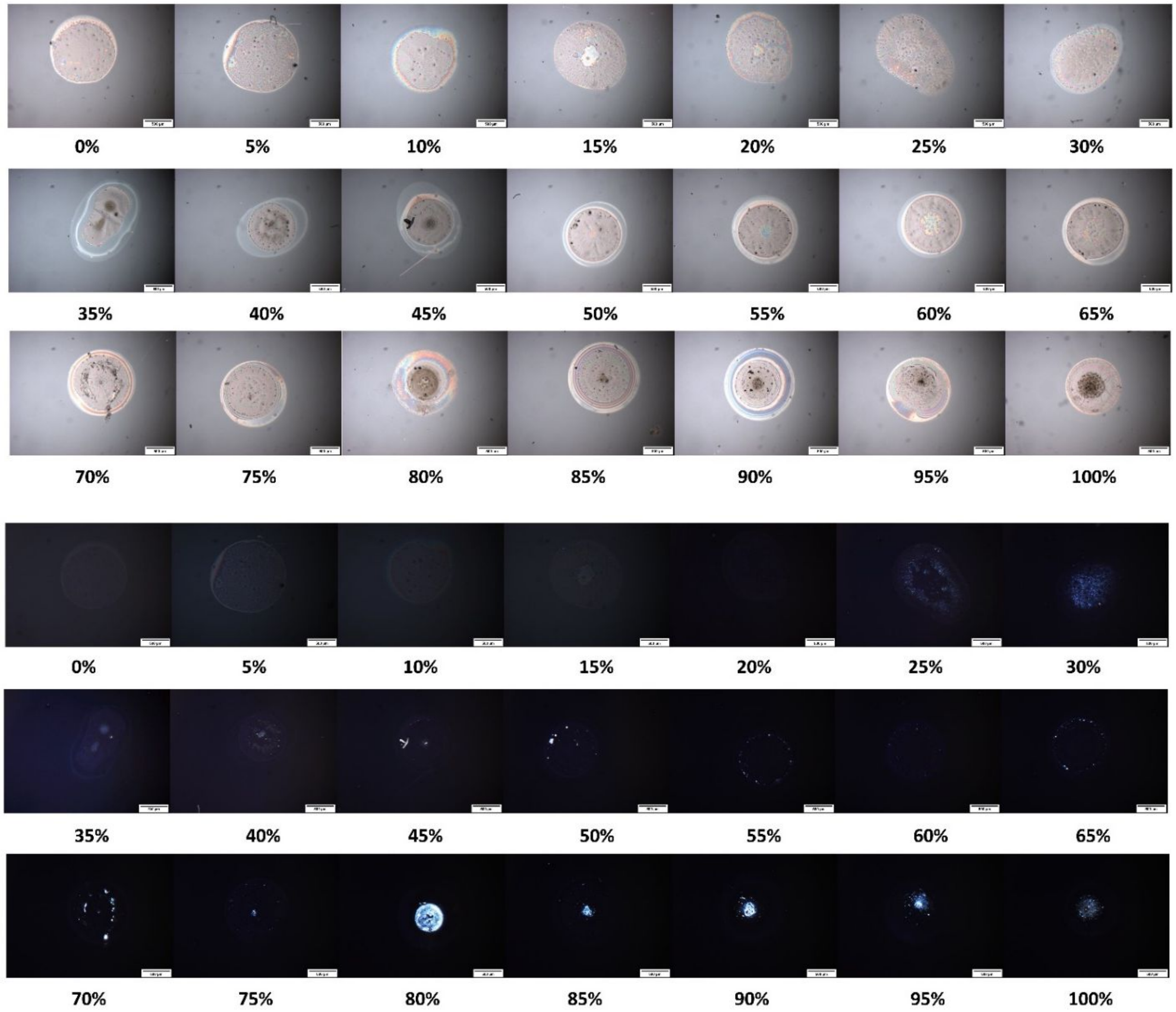

Figure S9. Microarray of Itraconazole/PVPVA depicted by PLM. All the spots are reported with the bright field in the upper part of the figure and cross-polarised filters in the lower part of the figure. The images show all different ratios of Itraconazole/PVPVA starting from 0% to 100% with a 5% Itraconazole increment. The final mass of individual spots is 1000-1100 ng. The scale bar represents 500µm.

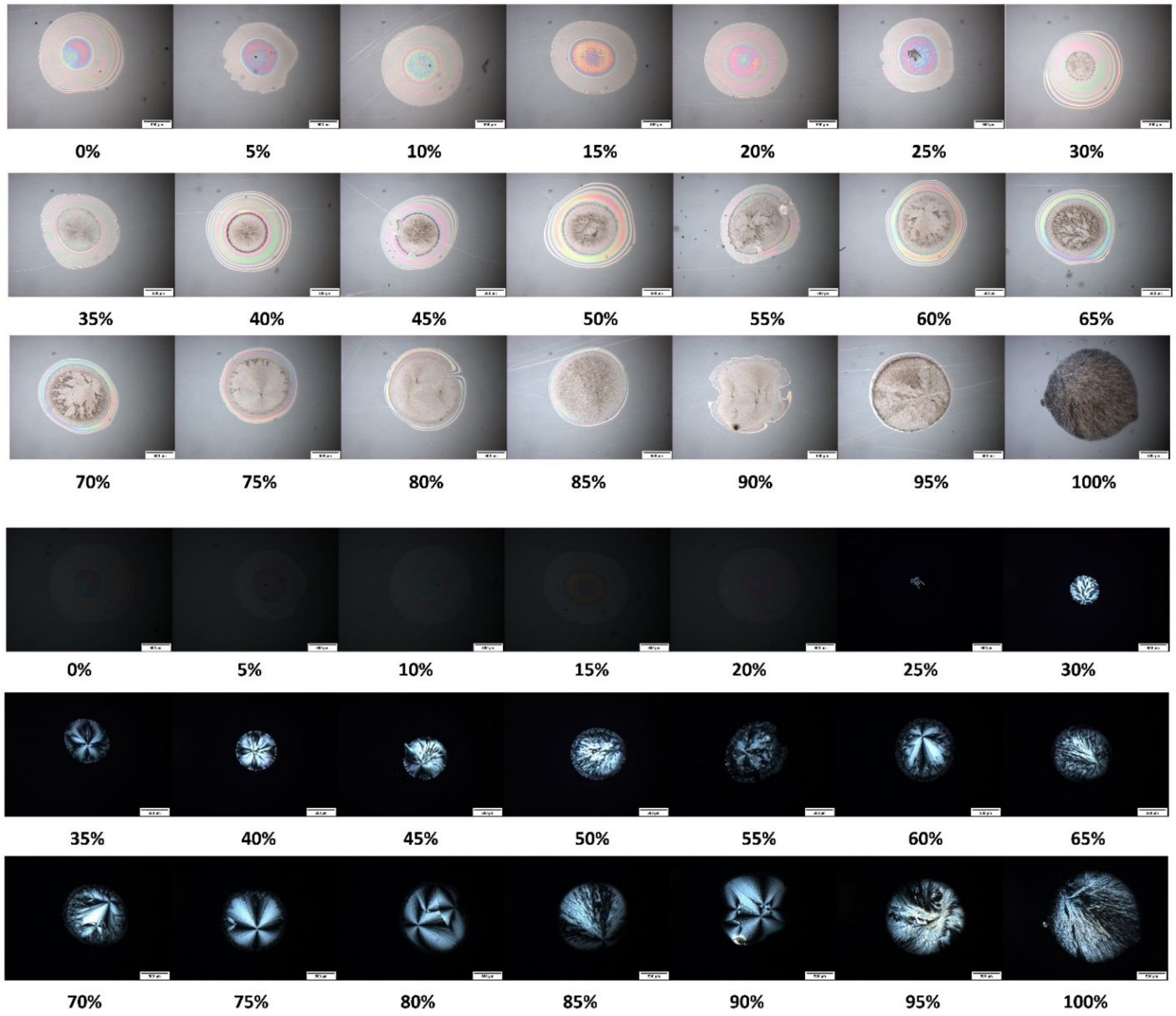

Figure S10. Microarray of Nicotinamide/PVPVA depicted by PLM. All the spots are reported with the bright field in the upper part of the figure and cross-polarised filters in the lower part of the figure. The images show all different ratios of Nicotinamide/PVPVA starting from 0% to 100% with a 5% Nicotinamide increment. The final mass of individual spots is 1000-1100 ng. The scale bar represents 500μm.

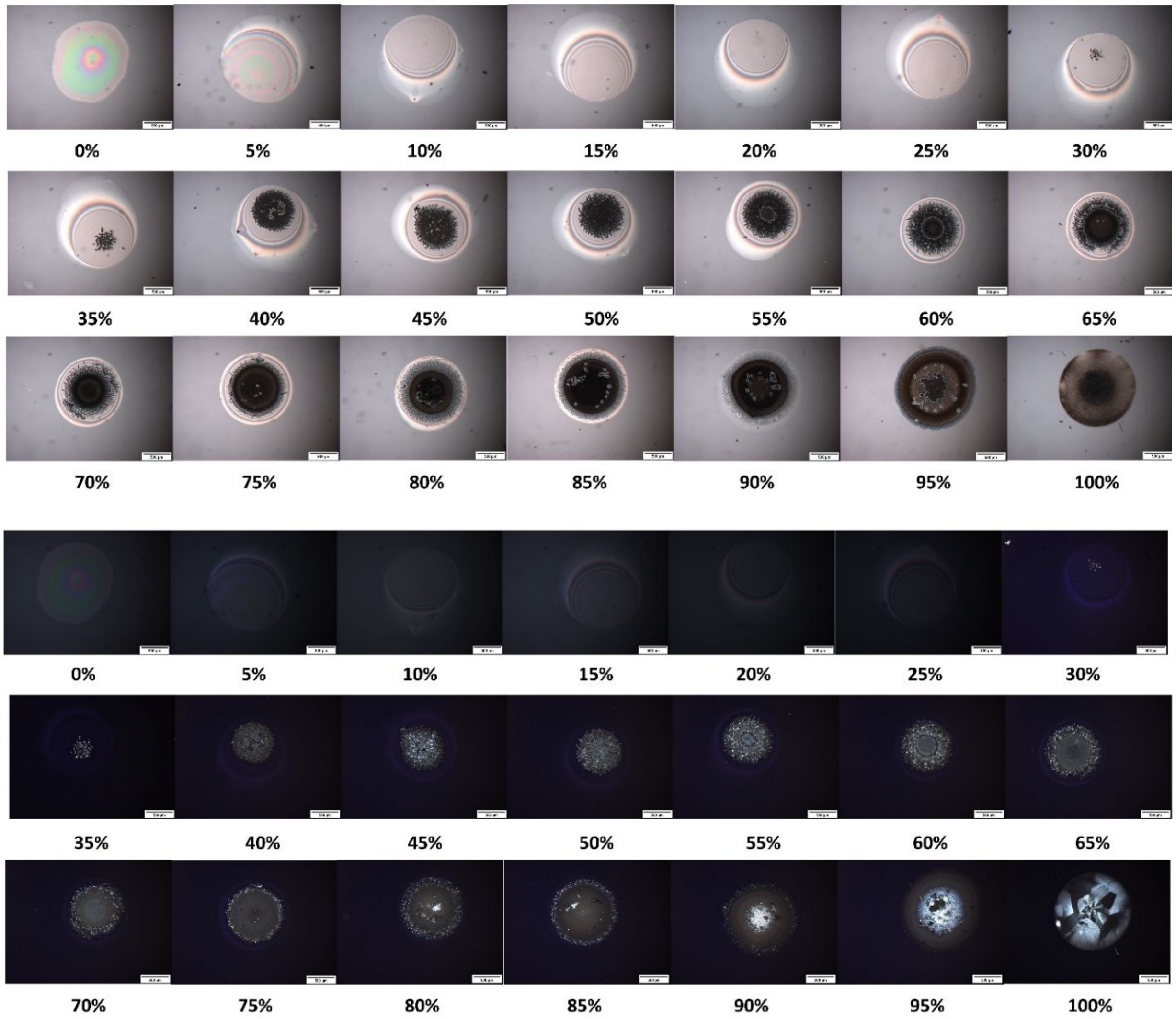

Figure S11. Microarray of ß-Estradiol/PVPVA depicted by PLM. All the spots are reported with the bright field in the upper part of the figure and cross-polarised filters in the lower part of the figure. The images show all different ratios of ß-Estradiol/PVPVA starting from 0% to 100% with a 5% ß-Estradiol increment. The final mass of individual spots is 1000-1100 ng. The scale bar represents 500µm.

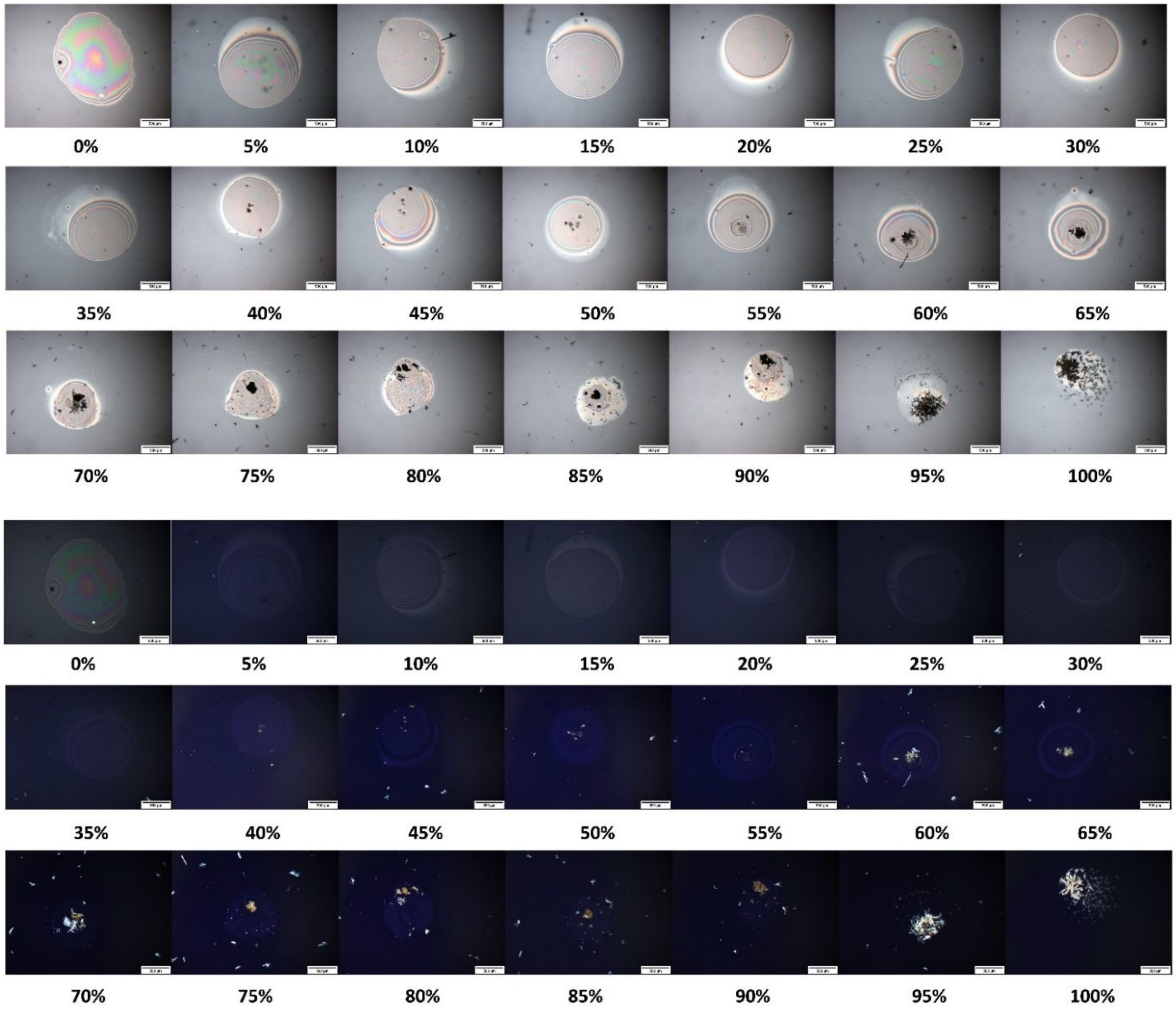

Figure S12. Microarray of Nifedipine/PVPVA depicted by PLM. All the spots are reported with the bright field in the upper part of the figure and cross-polarised filters in the lower part of the figure. The images show all different ratios of Nifedipine/PVPVA starting from 0% 100% with a 5% Nifedipine increment. The final mass of individual spots is 1000-1100 ng. The scale bar represents 500μm.

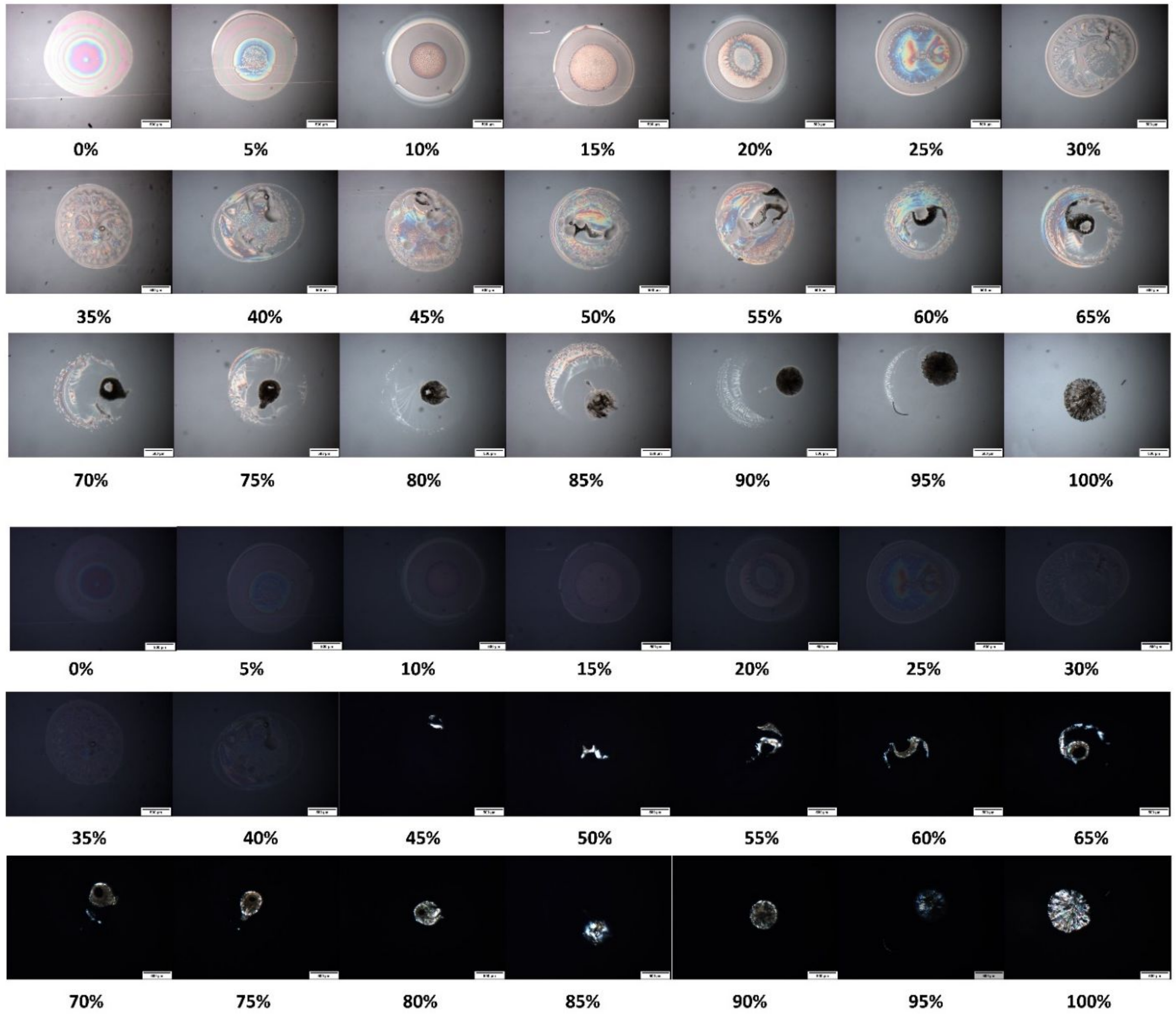

Figure S13. Microarray of Flufenamic acid/PVPVA depicted by PLM. All the spots are reported with the bright field in the upper part of the figure and cross-polarised filters in the lower part of the figure. The images show all different ratios of Flufenamic acid/PVPVA starting from 0% to 100% with a 5% Flufenamic acid increment. The final mass of individual spots is 1000-1100 ng. The scale bar represents 500μm.

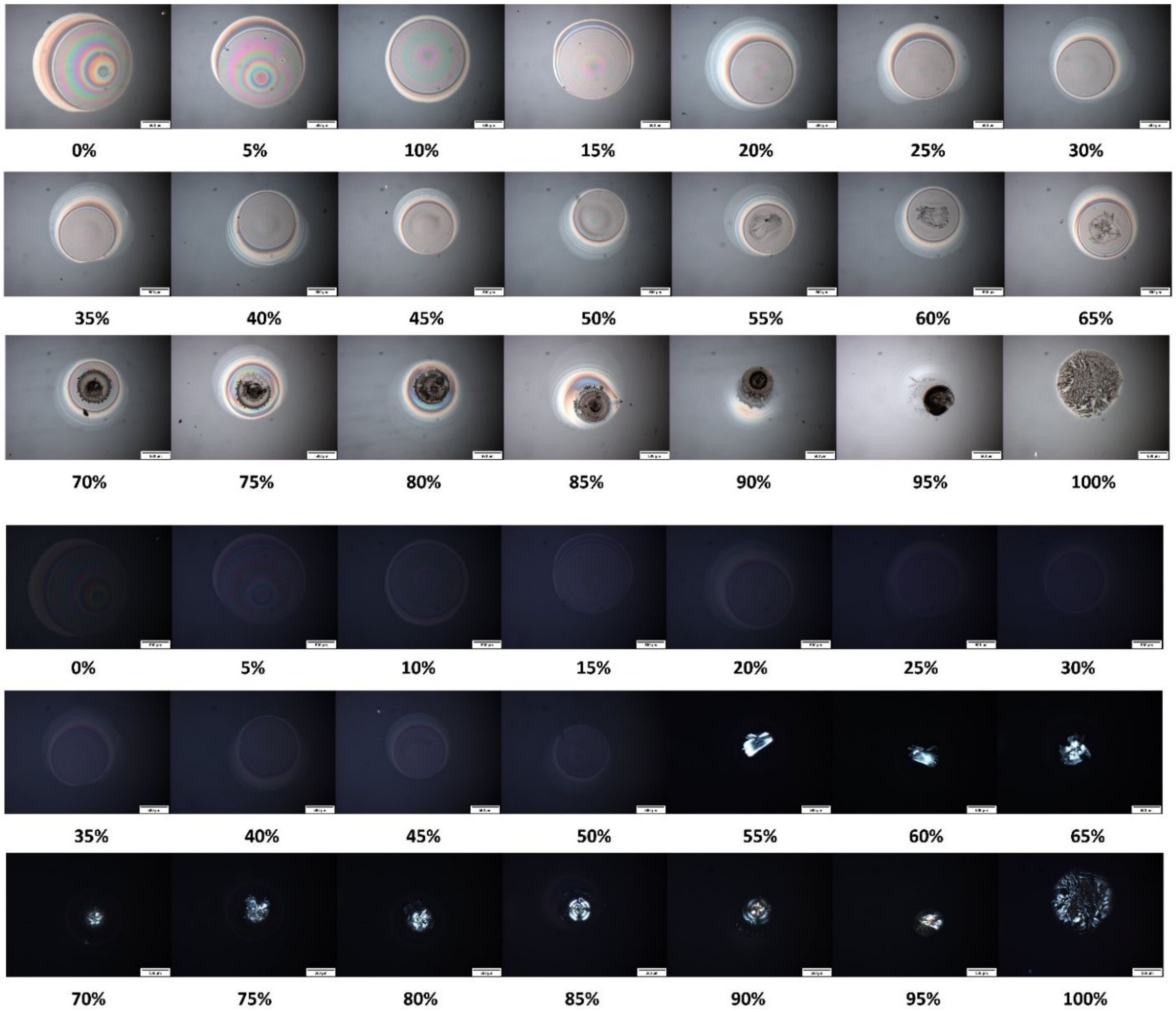

Figure S14. Microarray of Tolbutamide/PVPVA depicted by PLM. All the spots are reported with the bright field in the upper part of the figure and cross-polarised filters in the lower part of the figure. The images show all different ratios of Tolbutamide/PVPVA starting from 0% to 100% with a 5% Tolbutamide increment. The final mass of individual spots is 1000-1100 ng. The scale bar represents 500μm.

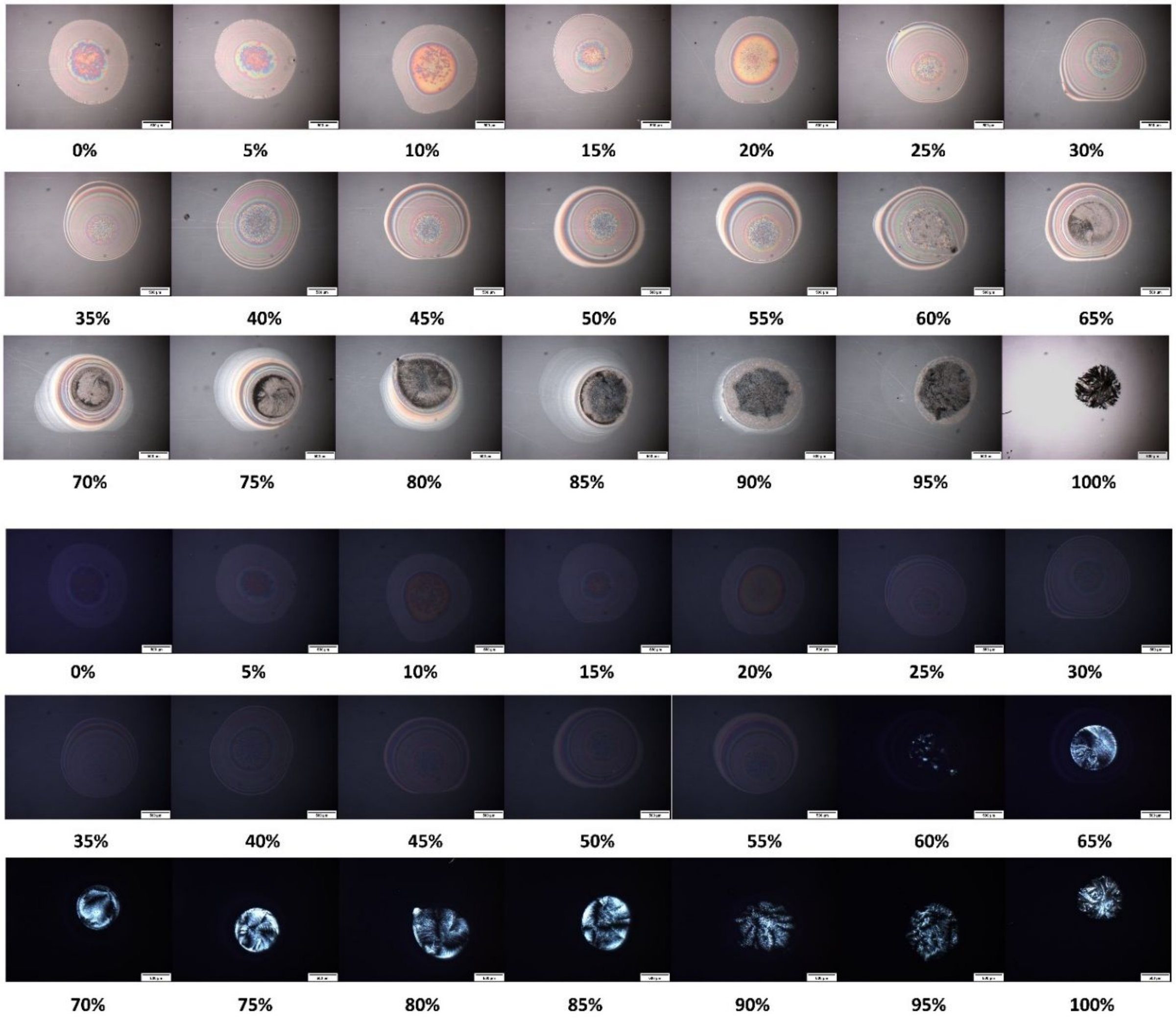

Figure S15. Microarray of Acetylsalicylic acid (Aspirin)/PVPVA depicted by PLM. All the spots are reported with the bright field in the upper part of the figure and cross-polarised filters in the lower part of the figure. The images show all different ratios of Aspirin/PVPVA starting from 0% to 100% with a 5% Aspirin increment. The final mass of individual spots is 1000-1100 ng. The scale bar represents 500μm.

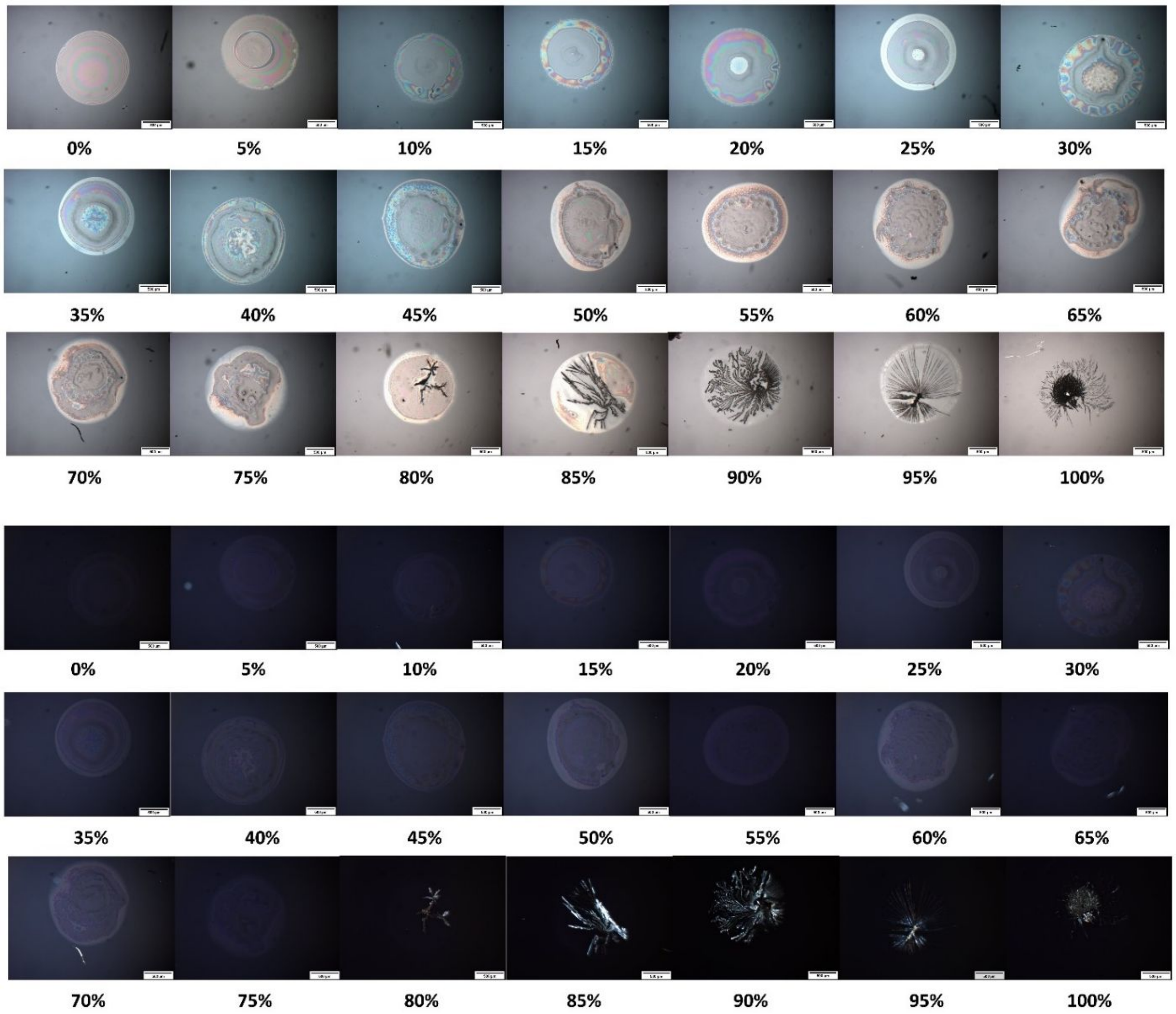

Figure S16. Microarray of Probucol/PVPVA depicted by PLM. All the spots are reported with the bright field in the upper part of the figure and cross-polarised filters in the lower part of the figure. The images show all different ratios of Probucol/PVPVA starting from 0% to 100% with a 5% Probucol increment. The final mass of individual spots is 1000-1100 ng. The scale bar represents 500μm.

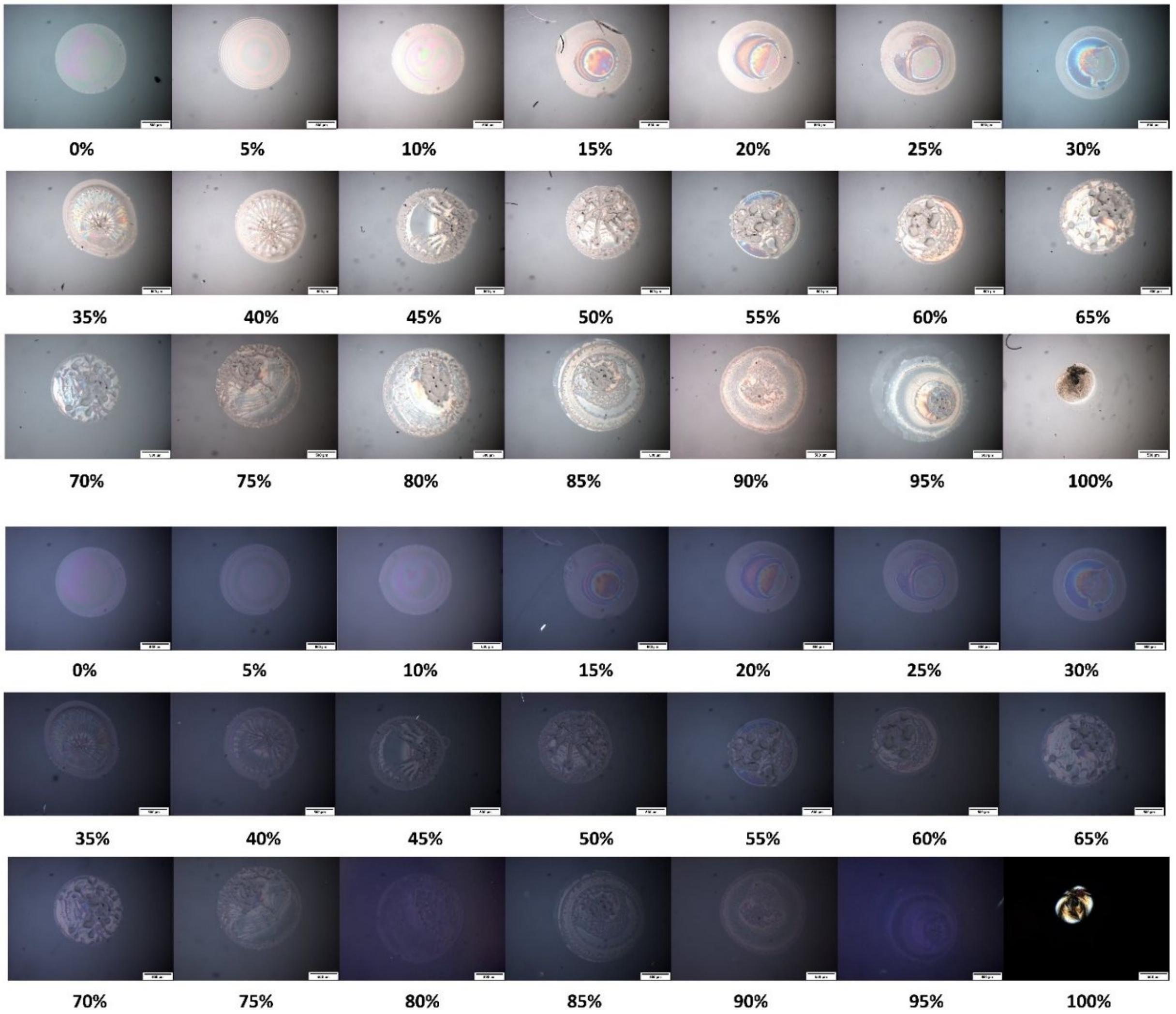

Figure S17. Microarray of Celecoxib/PVPVA depicted by PLM. All the spots are reported with the bright field in the upper part of the figure and cross-polarised filters in the lower part of the figure. The images show all different ratios of Celecoxib/PVPVA starting from 0% to 100% with a 5% Celecoxib increment. The final mass of individual spots is 1000-1100 ng. The scale bar represents 500μm.

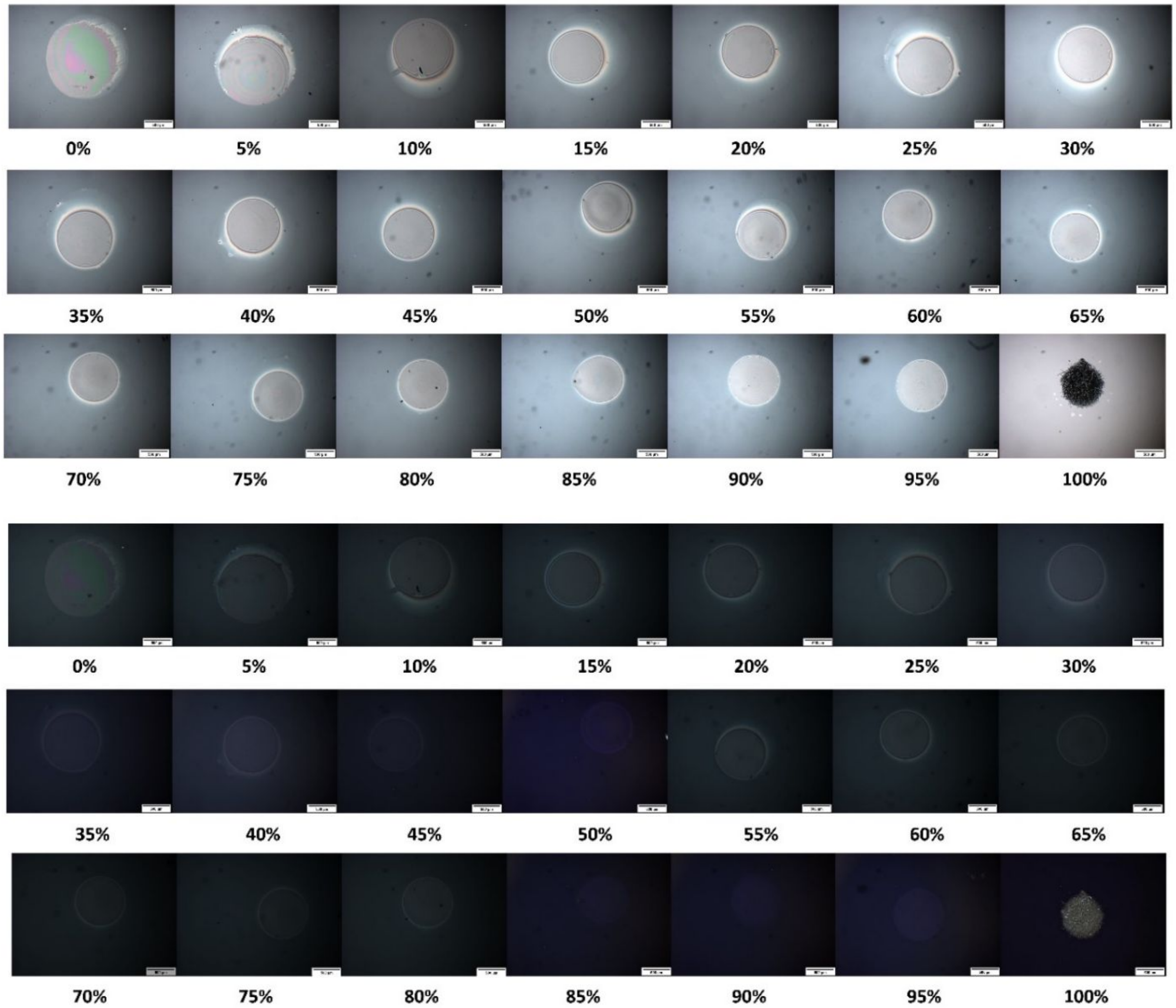

Figure S18. Microarray of Felodipine/PVPVA depicted by PLM. All the spots are reported with the bright field in the upper part of the figure and cross-polarised filters in the lower part of the figure. The images show all different ratios of Felodipine/PVPVA starting from 0% to 100% with a 5% Felodipine increment. The final mass of individual spots is 1000-1100 ng. The scale bar represents 500µm.

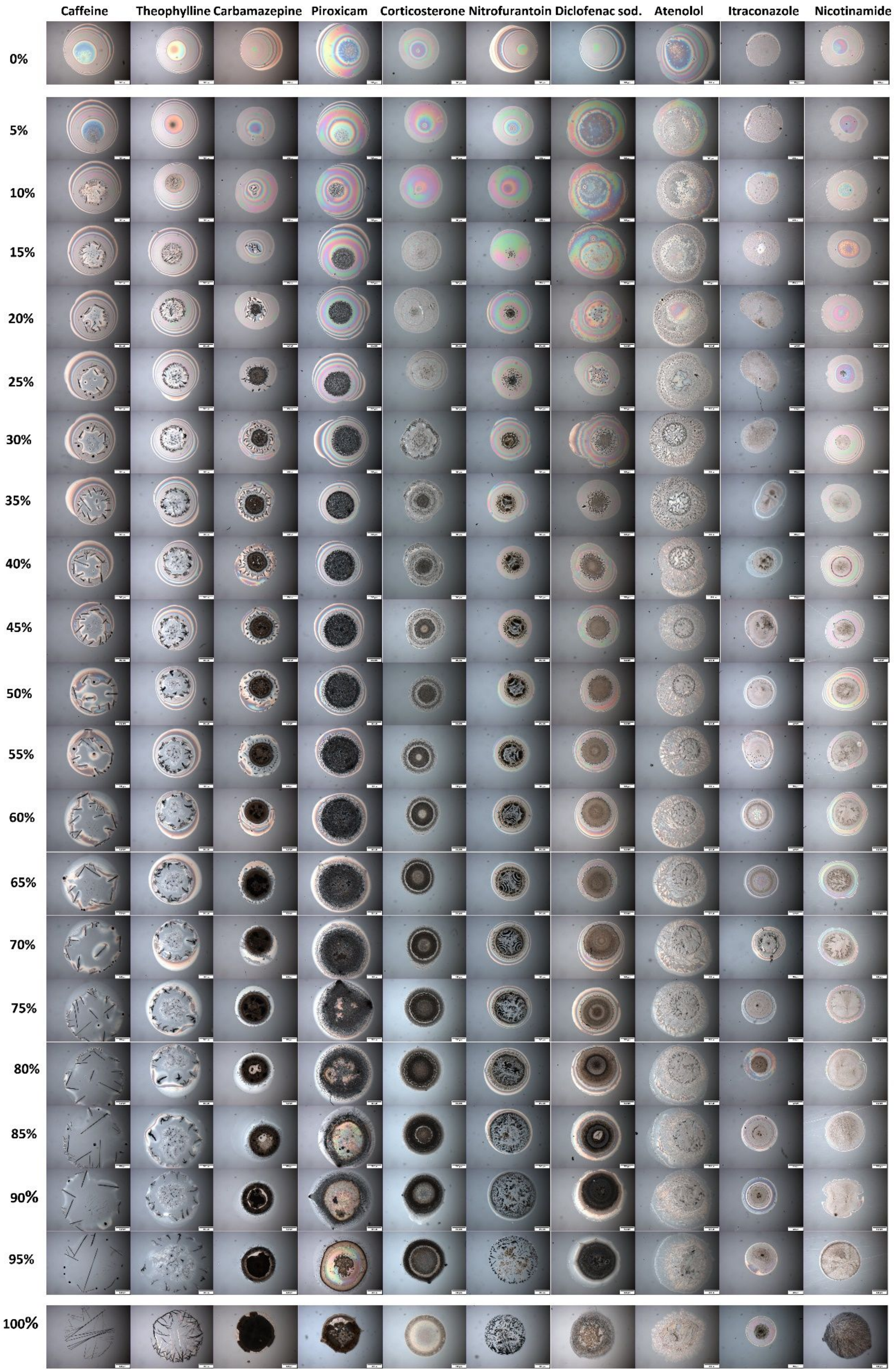
Caffeine
Theophylline
Carbamazepine
Piroxicam
Corticosterone
Nitrofurantoin
Diclofenac sod.
Atenolol
Itraconazole
Nicotinamide
0%
5%
10%
15%
20%
25%
30%
35%
40%
45%
50%
55%
60%
65%
70%
75%
80%
85%
90%
95%
100%

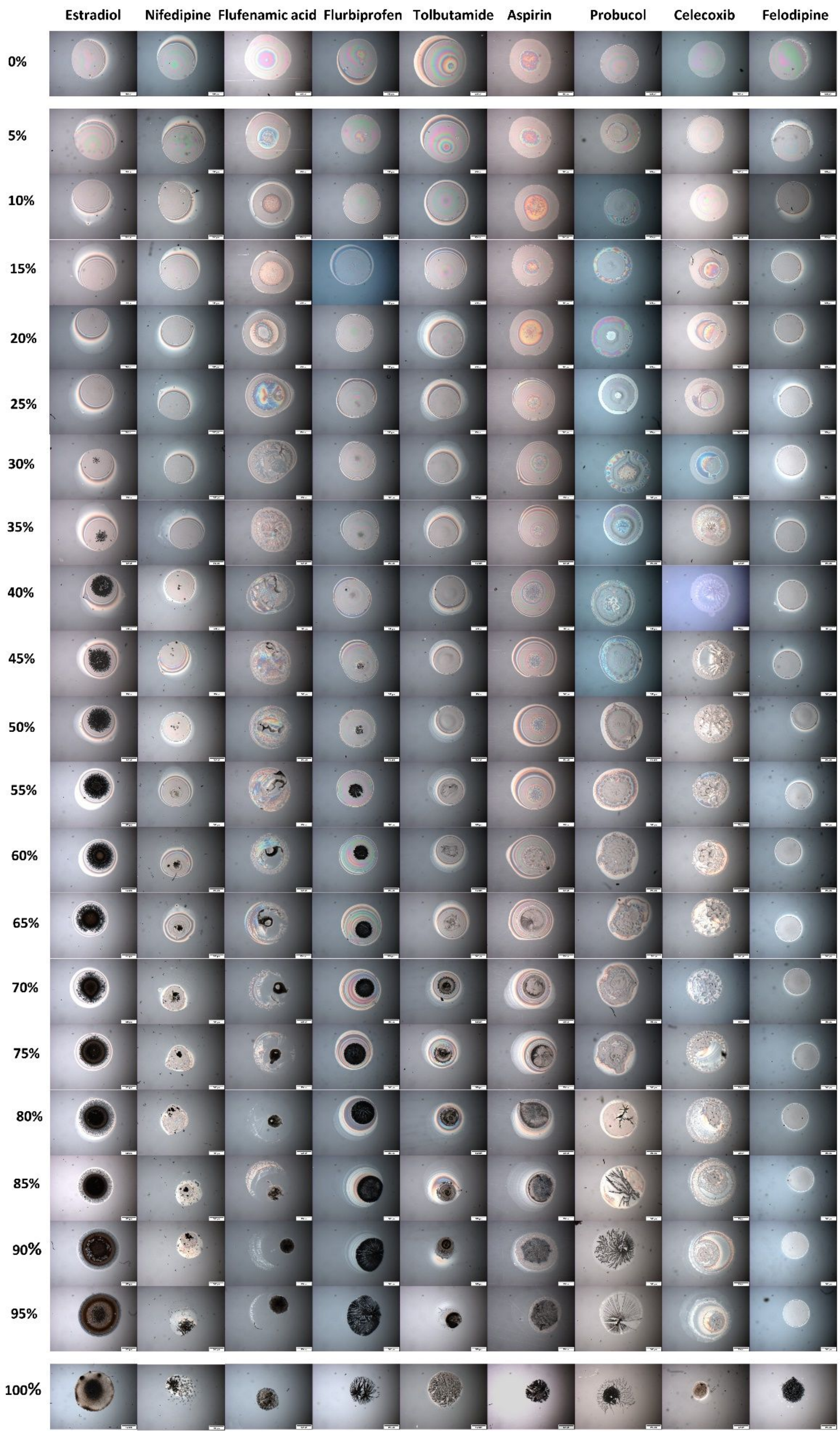
Estradiol
Nifedipine
Flufenamic acid
Flurbiprofen
Tolbutamide
Aspirin
Probucol
Celecoxib
Felodipine
0%
5%
10%
15%
20%
25%
30%
35%
40%
45%
50%
55%
60%
65%
70%
75%
80%
85%
90%
95%
100%

Figure S19. Examples of different APIs/PVPVA printed dispersions, starting from 0% to 100% API using 19 APIs and one polymer (PVPVA). All images are reported without cross-polarised filter. All images are reported after storage of the microarrays in accelerated conditions for 6 months (75% relative humidity and 40˚C in a stability oven). Final mass 1000-1100 ng.

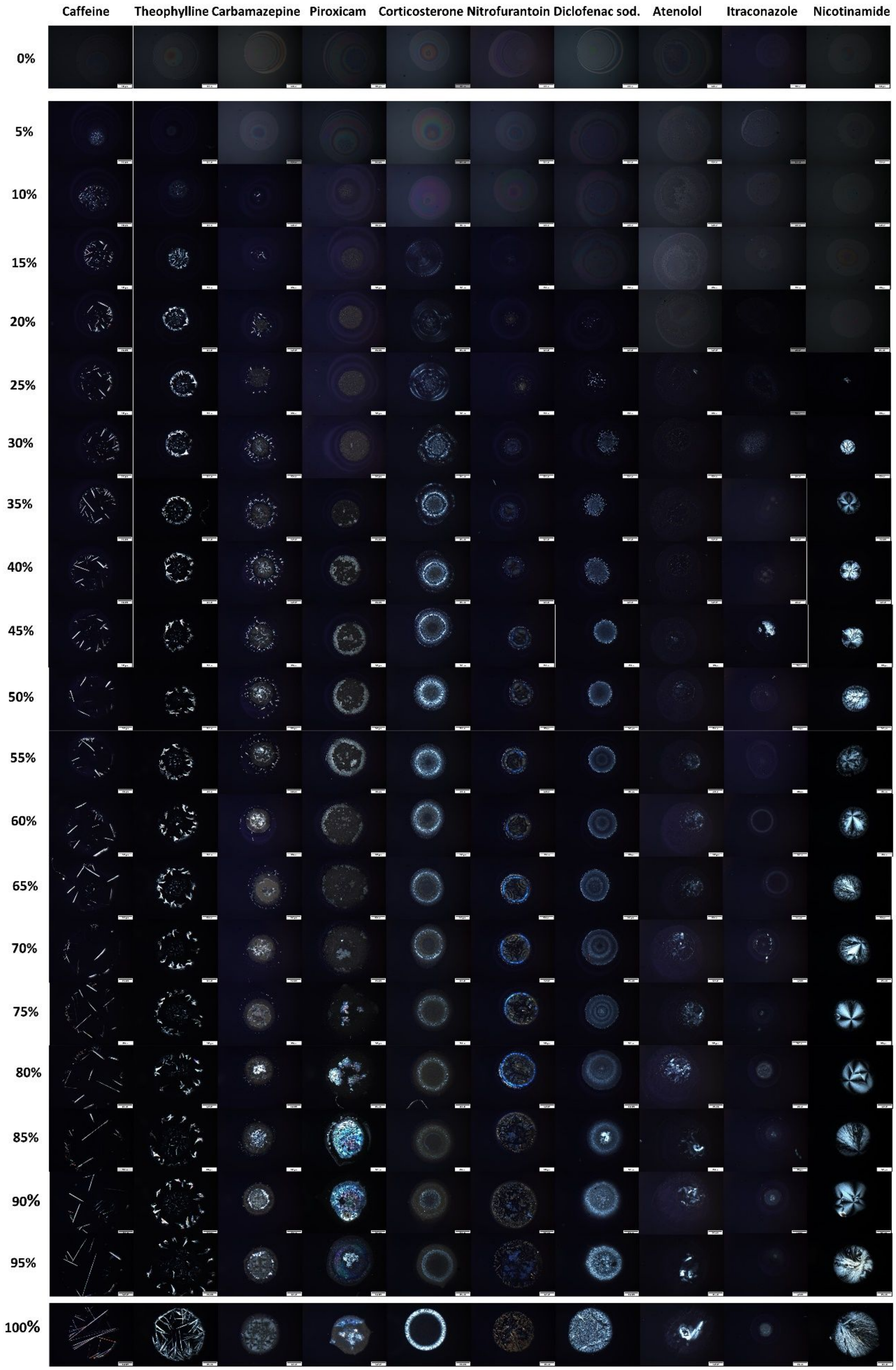
Caffeine
Theophylline
Carbamazepine
Piroxicam
Corticosterone
Nitrofurantoin
Diclofenac sod.
Atenolol
Itraconazole
Nicotinamide
0%
5%
10%
15%
20%
25%
30%
35%
40%
45%
50%
55%
60%
65%
70%
75%
80%
85%
90%
95%
100%

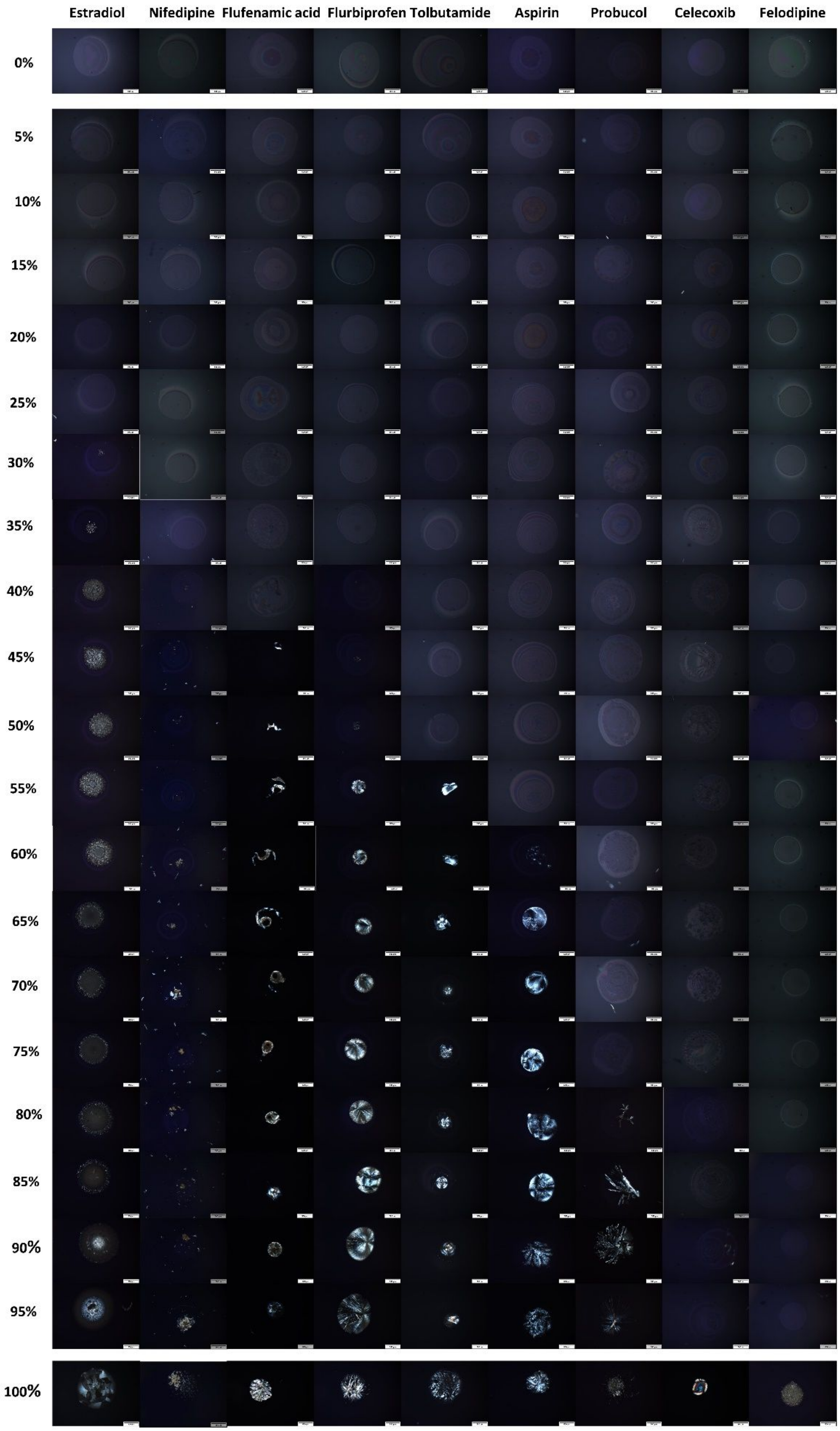
Estradiol
Nifedipine
Flufenamic acid
Flurbiprofen
Tolbutamide
Aspirin
Probucol
Celecoxib
Felodipine
0%
5%
10%
15%
20%
25%
30%
35%
40%
45%
50%
55%
60%
65%
70%
75%
80%
85%
90%
95%
100%

Figure S20. Examples of different APIs/PVPVA printed dispersions using cross-polarised filters, starting from 0% to 100% API using 19 APIs and one polymer (PVPVA). All images are reported after storage of the microarrays in accelerated conditions for 6 months (75% relative humidity and 40˚C in a stability oven). Final mass 1000-1100 ng.

2. Comparing Crystallisation Behavior of 2D inkjet printed APIs in our study to the Spin Coated APIs of Van Edinburgh

Table S1. Comparing crystallisation Behavior Following 2D inkjet printing stored in accelerated conditions for 6 months versus following Spin Coating stored in dry conditions for 7 days.
Class I (rapid crystallisation), APIs as Class II (intermediate crystallisation), and Class III (slow crystallisation).

API	GFA classification according to 2D inkjet printing in our study (6 months accelerated conditions)	GFA classification according to Van Edinburgh Spin Coating (7 days dry conditions)
Caffeine	I	I
Flufenamic acid	I	I
Tolbutamide	I	I
Benzocaine	I	I
Flurbiprofen	I	I
Carbamazepine	I	II
Itraconazole	I	III
Atenolol	I	III
Probucol	II	II
Celecoxib	II	III
Felodipine	II	III
Fenofibrate	III	II
Ritonavir	III	III
Ketoprofen	III	III
Paracetamol	III	III
Theophylline	I	
Piroxicam	I	
Corticosterone	I	
Nitrofurantoin	I	
Diclofenac sodium	I	
Nicotinamide	I	
Estradiol	I	
Aspirin	I	
Nifedipine	II	
Chlorpromazine HCl	III	
Dexamethasone phosphate	III	
Aprepitant	III	
Orlistat	III	
Ampicillin	III	

Coumarin	III	

3. Stability data of printed API/PVPVA microarrays

Table S2. Stability of the printed microarrays of each API with PVPVA in API/polymer loading from 5% to 100% with 5% API increment (the last time point where no crystals were observed represented in days). Microarrays were kept in accelerated conditions (40˚C ± 2˚C/75% RH ± 5% RH) for six months.
N: no crystallisation was monitored for those samples during the whole period of the study.

API/PVPVA loading w/w%	0	5	10	15	20	25	30	35	40	45	50	55	60	65	70	75	80	85	90	95	100
**Caffeine**	N	0	0	0	0	0	0	0	0	0	0	0	0	0	0	0	0	0	0	0	0
**Theophylline**	N	0	0	0	0	0	0	0	0	0	0	0	0	0	0	0	0	0	0	0	0
**Carbamazepine**	N	N	4	3	3	2	2	2	2	2	2	2	2	2	2	2	1	1	0	0	0
**Piroxicam**	N	N	4	2	2	2	2	2	2	2	2	2	2	2	2	2	2	2	1	0	0
**Corticosterone**	N	N	N	4	4	2	2	2	2	2	2	2	2	2	2	2	2	1	1	0	0
**Nitrofurantoin**	N	N	N	3	1	1	0	0	0	0	0	0	0	0	0	0	0	0	0	0	0
**Diclofenac sodium**	N	N	N	N	2	2	2	2	2	2	2	2	2	1	1	0	0	0	0	0	0
**Atenolol**	N	N	N	N	N	2	1	0	0	0	0	0	0	0	0	0	0	0	0	0	0
**Itraconazole**	N	N	N	N	N	55	10	4	2	2	2	2	0	0	0	0	0	0	0	0	0
**Nicotinamide**	N	N	N	N	N	3	3	1	1	0	0	0	0	0	0	0	0	0	0	0	0
**Estradiol**	N	N	N	N	N	N	72	10	4	2	1	1	1	1	1	1	1	1	0	0	0
**Nifedipine**	N	N	N	N	N	N	N	N	138	138	138	138	76	55	55	55	55	12	10	10	4
**Flufenamic acid**	N	N	N	N	N	N	N	N	N	1	1	1	0	0	0	0	0	0	0	0	0
**Flurbiprofen**	N	N	N	N	N	N	N	N	N	4	2	2	2	2	1	1	0	0	0	0	0
**Tolbutamide**	N	N	N	N	N	N	N	N	N	N	N	4	2	2	1	0	0	0	0	0	0
**Aspirin**	N	N	N	N	N	N	N	N	N	N	N	N	0	0	0	0	0	0	0	0	0
**Probucol**	N	N	N	N	N	N	N	N	N	N	N	N	N	N	N	N	81	81	81	72	55
**Celecoxib**	N	N	N	N	N	N	N	N	N	N	N	N	N	N	N	N	N	N	N	N	55
**Felodipine**	N	N	N	N	N	N	N	N	N	N	N	N	N	N	N	N	N	N	N	N	55
**Ritonavir**	N	N	N	N	N	N	N	N	N	N	N	N	N	N	N	N	N	N	N	N	N
**Fenofibrate**	N	N	N	N	N	N	N	N	N	N	N	N	N	N	N	N	N	N	N	N	N
**Aprepitant**	N	N	N	N	N	N	N	N	N	N	N	N	N	N	N	N	N	N	N	N	N
**Orlistat**	N	N	N	N	N	N	N	N	N	N	N	N	N	N	N	N	N	N	N	N	N

4. Drop-Cast technique

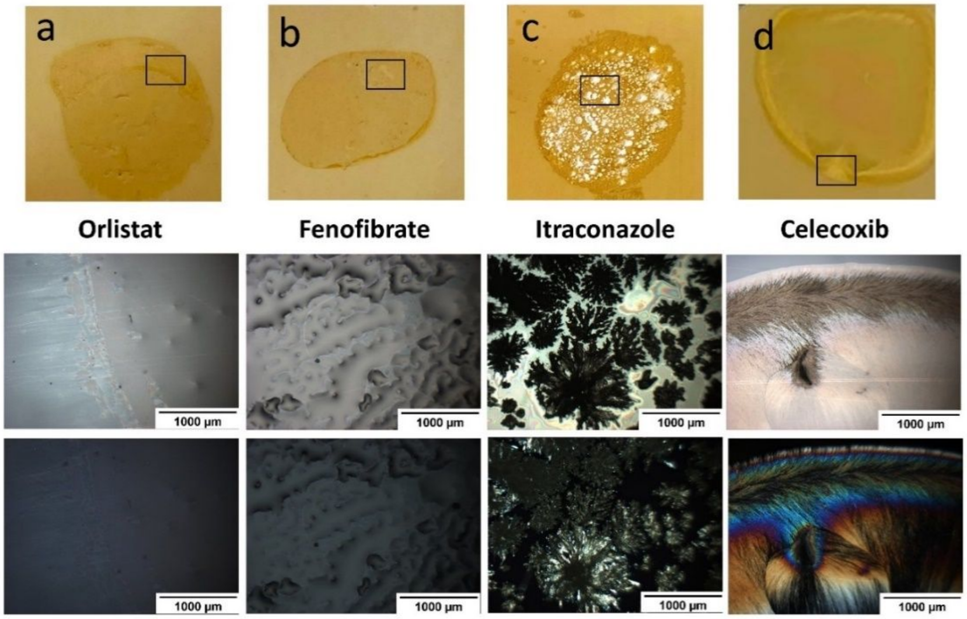

Figure S21. The top row shows the spots by drop cast technique for pure APIs pipetted on gold-coated glass slides as substrate. a) pure Orlistat (0%), b) pure Fenofibrate, c) pure Itraconazole, d) pure Celecoxib. Images were depicted as resembling a small section taken with optical (middle row) and cross-polarised (bottom row).

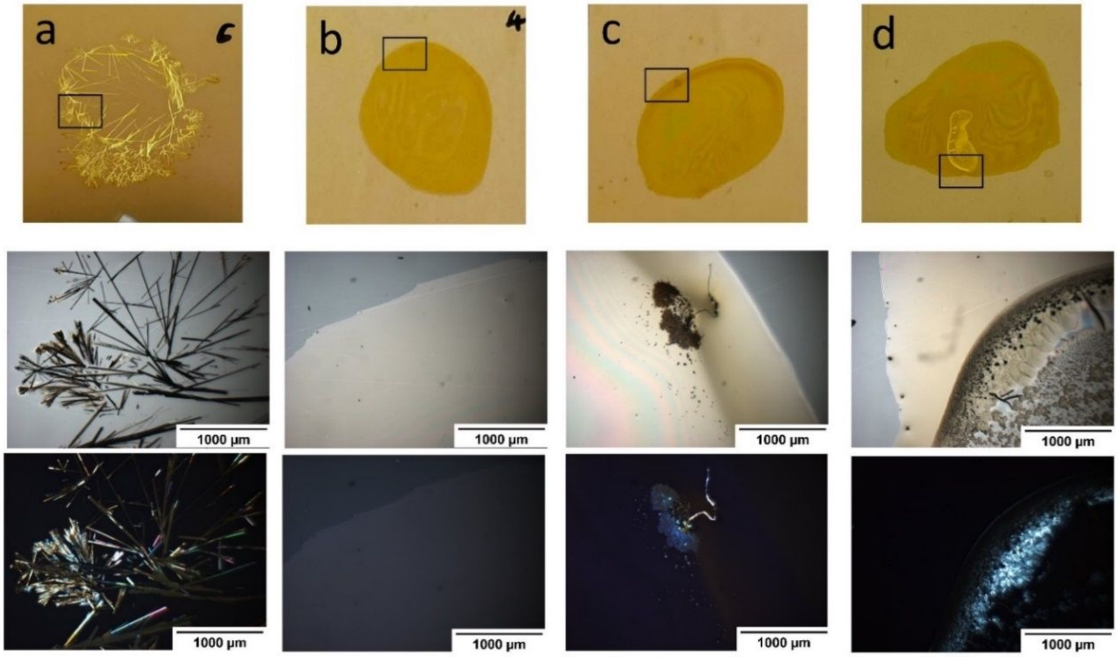

Figure S22. The top row shows the spots by drop cast technique for Nitrofurantoin/PVPVA pipetted on gold-coated glass slides as substrate. a) Pure Nitrofurantoin (0%), b) 10% Nitrofurantoin/PVPVA, c) 15% Nitrofurantoin/PVPVA,

d) 25% Nitrofurantoin/PVPVA. Images were depicted as resembling a small section taken with optical (middle row) and cross-polarised (bottom row).

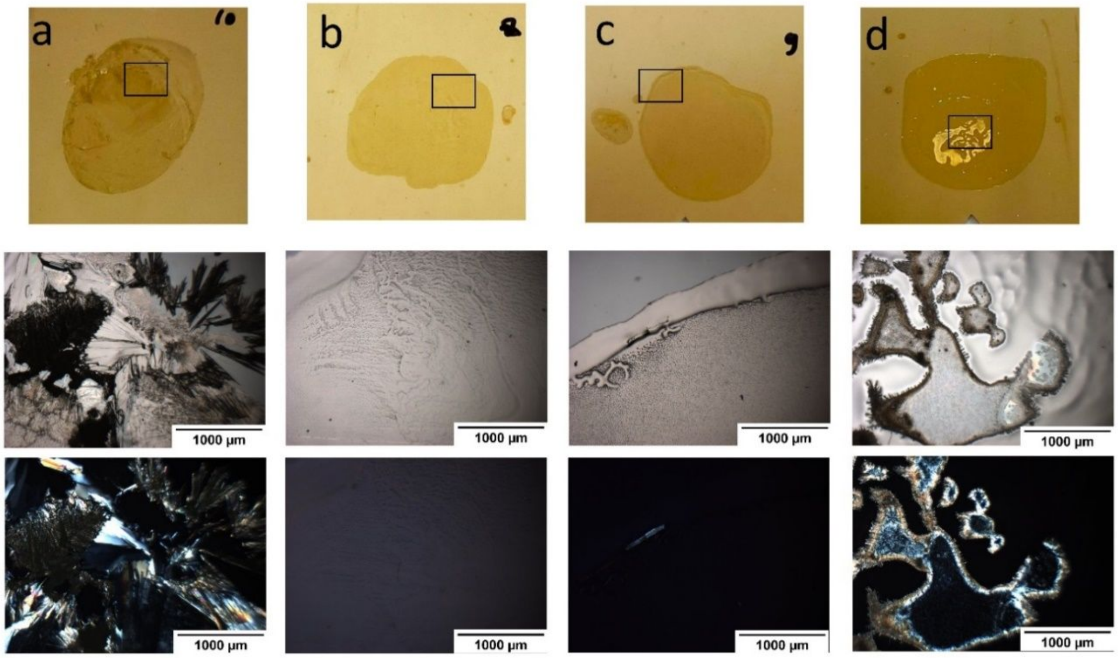

Figure S23. The top row shows the spots by drop cast technique for Flufenamic acid/PVPVA pipetted on gold-coated glass slides as substrate. a) Pure Flufenamic acid (0%), b) 40% Flufenamic acid/PVPVA, c) 45% Flufenamic acid/PVPVA, d) 55% Flufenamic acid/PVPVA. Images were depicted as resembling a small section taken with optical (middle row) and cross-polarised (bottom row).

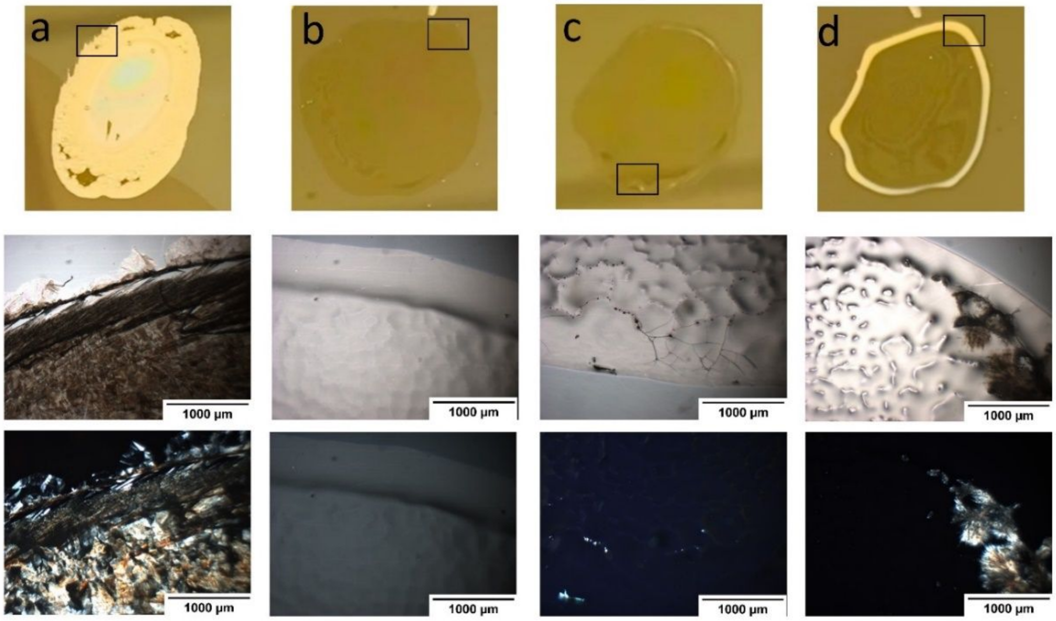

Figure S24. The top row shows the spots by drop cast technique for Estradiol/PVPVA pipetted on gold-coated glass slides as substrate. a) Pure Estradiol (0%), b) 25% Estradiol/PVPVA, c) 30% Estradiol/PVPVA, d) 40%

Estradiol/PVPVA. Images were depicted as resembling a small section taken with optical (middle row) and cross-polarised (bottom row).

5. Multiple Linear Regression Model Development

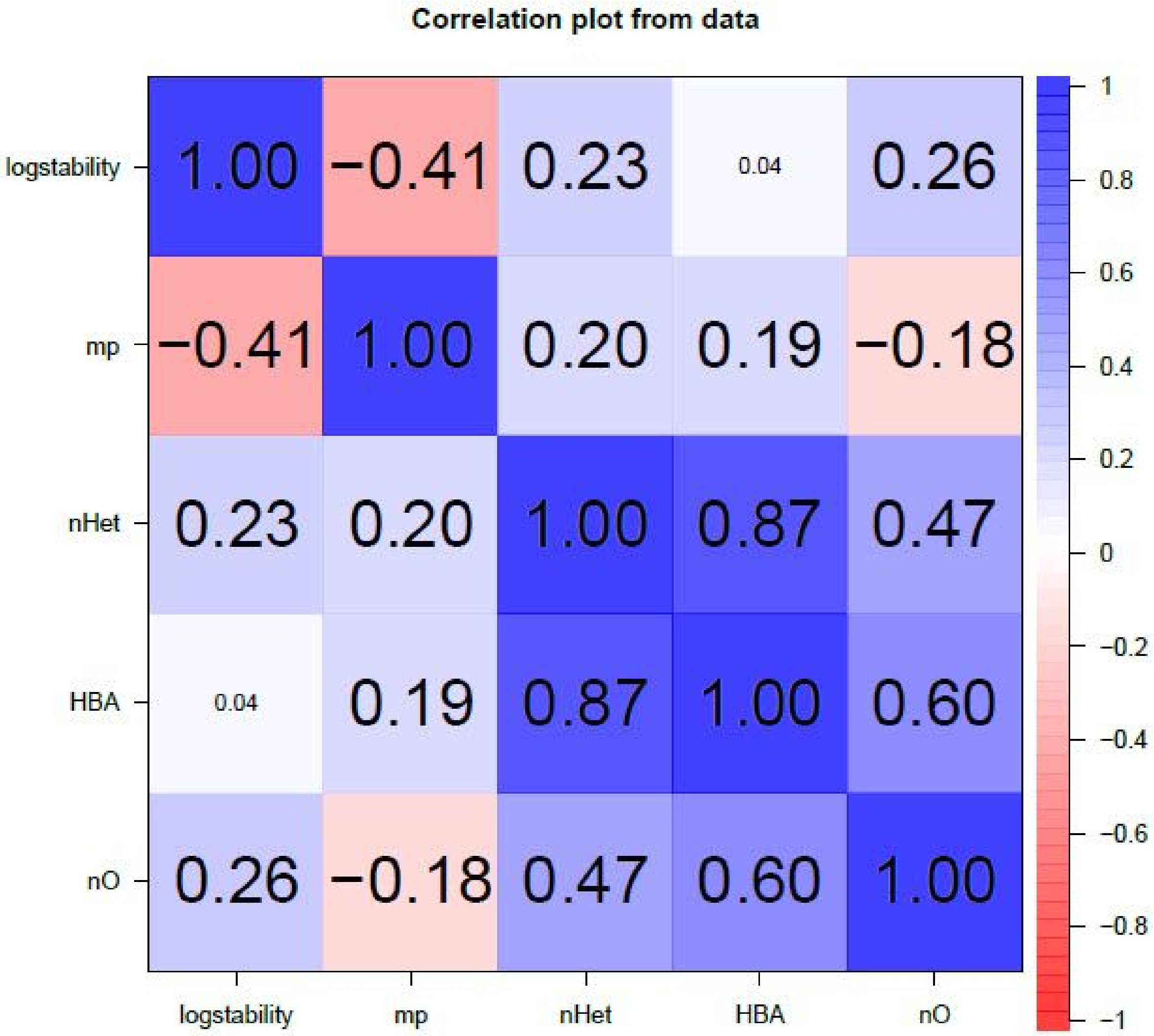

Figure S25. The correlation matrix displays the pairwise correlations between the predictor variables and log stability within PVPVA. Each cell represents the correlation coefficient between the corresponding variables. These correlations are not derived from the linear regression model. (mp: melting point, nHet: number of heteroatoms, HBA: number of hydrogen bond acceptors and nO: number of oxygen atoms).

Table S3. Equations extracted from the model using the PVPVA data set and all drug/polymer loadings 5 to 100%.

Drug/polymer loading	Equation	Adjusted R^2
**5**	logstability= 6.996475 +0.246874 HBA -0.059707 nHet + 0.108801 nO -0.012858 mp	0.1906
**10**	logstability= 6.719668 +0.191279 HBA +0.02490 nHet + 0.130794 nO -0.016460 mp	0.2197
**15**	logstability= 8.506367+0.122059 HBA +0.138404 nHet -0.258290 nO -0.024924 mp	0.3289
**20**	logstability= 9.476716 -0.148043 HBA +0.311623 nHet -0.265762 nO -0.033630 mp	0.5874
**25**	logstability= 7.794643 -0.992177 HBA +0.591371 nHet +0.227512 nO -0.028057 mp	0.5338
**30**	logstability= 7.704040 -1.270611 HBA+ 0.699285 nHet+ 0.310144 nO -0.028329 mp	0.6001
**35**	logstability= 7.802053 -1.482707 HBA+ 0.786873 nHet + 0.332984 nO -0.028699 mp	0.6241
**40**	logstability= 6.272624 -1.775292 HBA+ 0.921450 nHet + 0.575989 nO -0.025085 mp	0.6546
**45**	logstability= 3.690459 -1.865890 HBA+ 0.936704 nHet+ 0.948554 nO -0.018149 mp	0.6354
**50**	logstability= 3.376875 -1.900602 HBA+ 0.959294 nHet + 0.992693 nO -0.017569 mp	0.6343
**55**	logstability= 2.869633 -1.851043 HBA+0.972986 nHet + 0.918478 nO -0.016115 mp	0.6187

**60**	logstability= 2.738579 -2.057051 HBA+ 1.050360 nHet + 0.903640 nO -0.014844 mp	0.6217
**65**	logstability= 2.816804 -2.055402 HBA+ 1.056535 nHet+ 0.885434 nO -0.015492 mp	0.6278
**70**	logstability= 2.579960 -2.072312 HBA+1.101958 nHet+ 0.819226 nO -0.014995 mp	0.6342
**75**	logstability= 2.583526 -2.057554 HBA+ 1.099058 nHe + 0.830711 nO -0.015733 mp	0.6321
**80**	logstability= 1.852182 -2.018366 HBA+ 1.100582 nHet+ 0.900466 nO -0.014425 mp	0.6629
**85**	logstability= 2.287267 -2.029673 HBA+1.142115 nHet + 0.711278 nO -0.015562 mp	0.6782
**90**	logstability= 2.085831 -2.002394 HBA+ 1.146997 nHet+ 0.724524 nO -0.015922 mp	0.6867
**95**	logstability= 2.263351 -2.069877 HBA+ 1.181978 nHet + 0.696395 nO -0.016566 mp	0.691
**100**	logstability= 2.154057 -1.843040 HBA+ 1.074906 nHet+ 0.628304 nO -0.016293 mp	0.6749

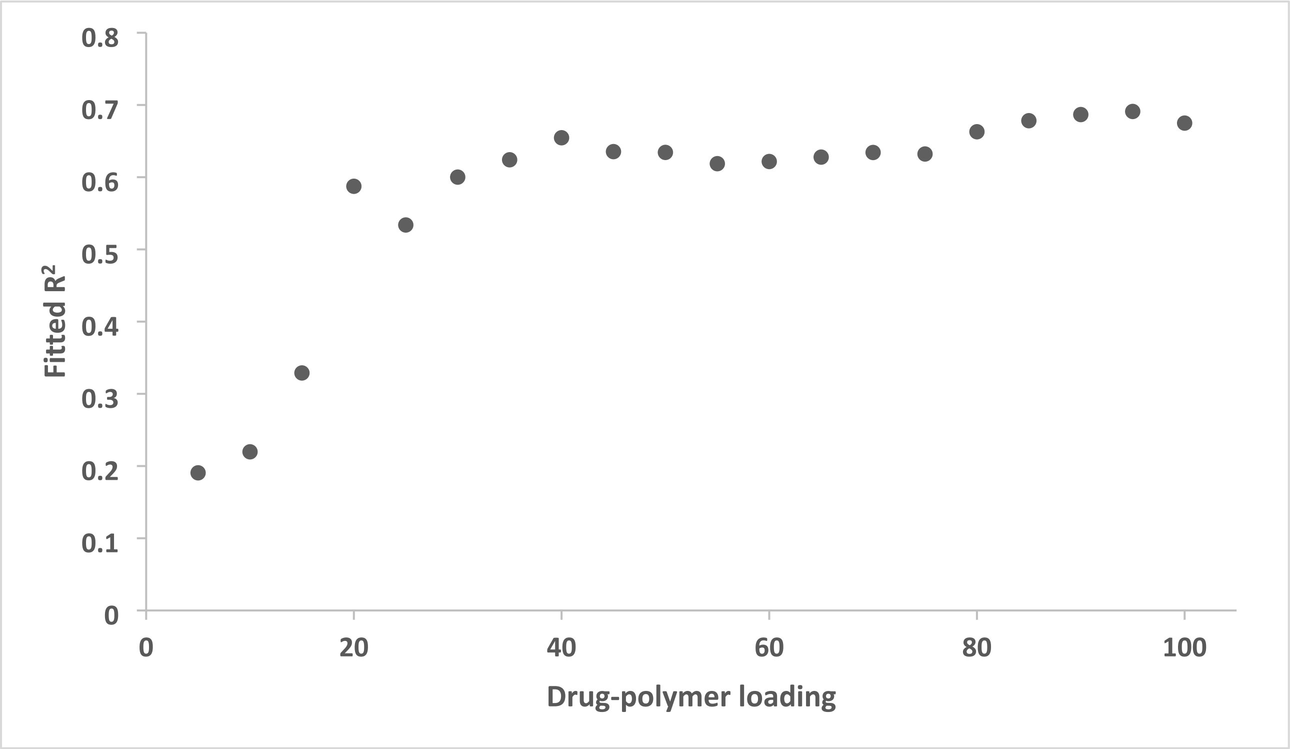

Figure S26. Fitted R^2 extracted from the multiple linear regression model for the PVPVA data set, using all drug/polymer loadings (5-100% with 5% increment) for 23 employed APIs.

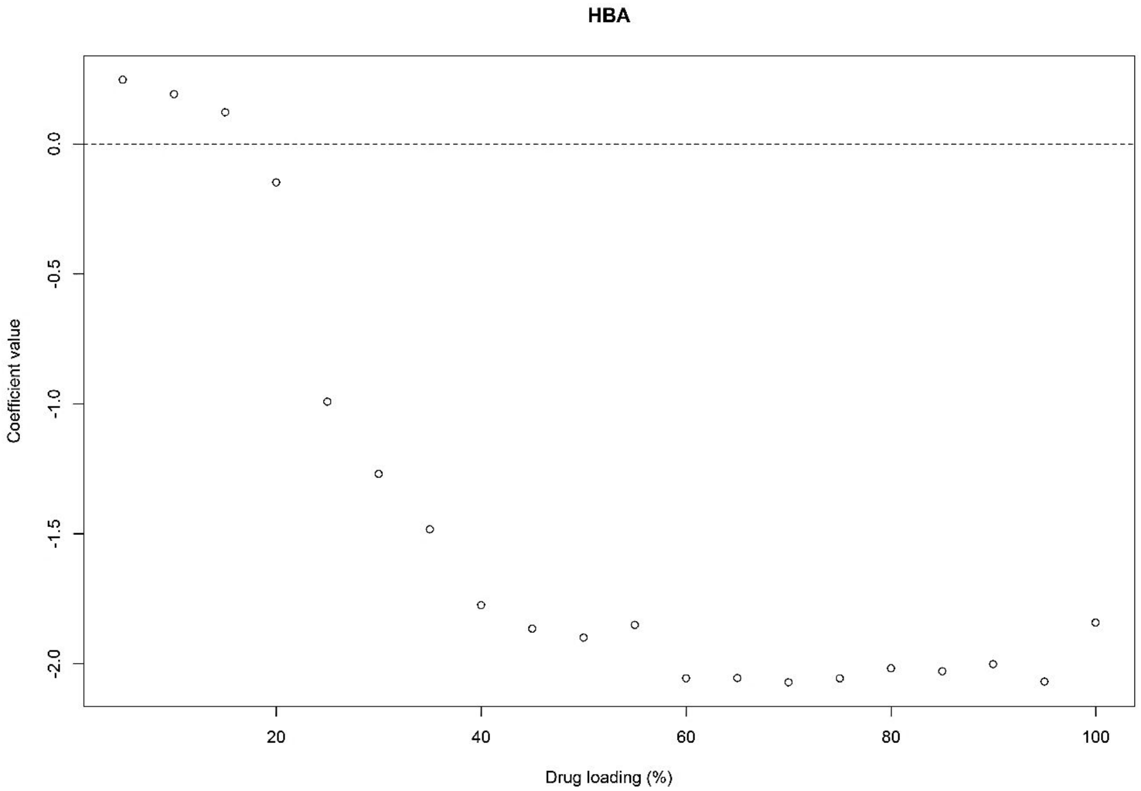

Figure S27. Scatter plot of coefficient values for hydrogen bond acceptors (HBA) variable obtained from multiple linear regression analyses across 23 different drugs at varying drug/polymer loadings from 5 to 100% using the data collected from the two polymers.

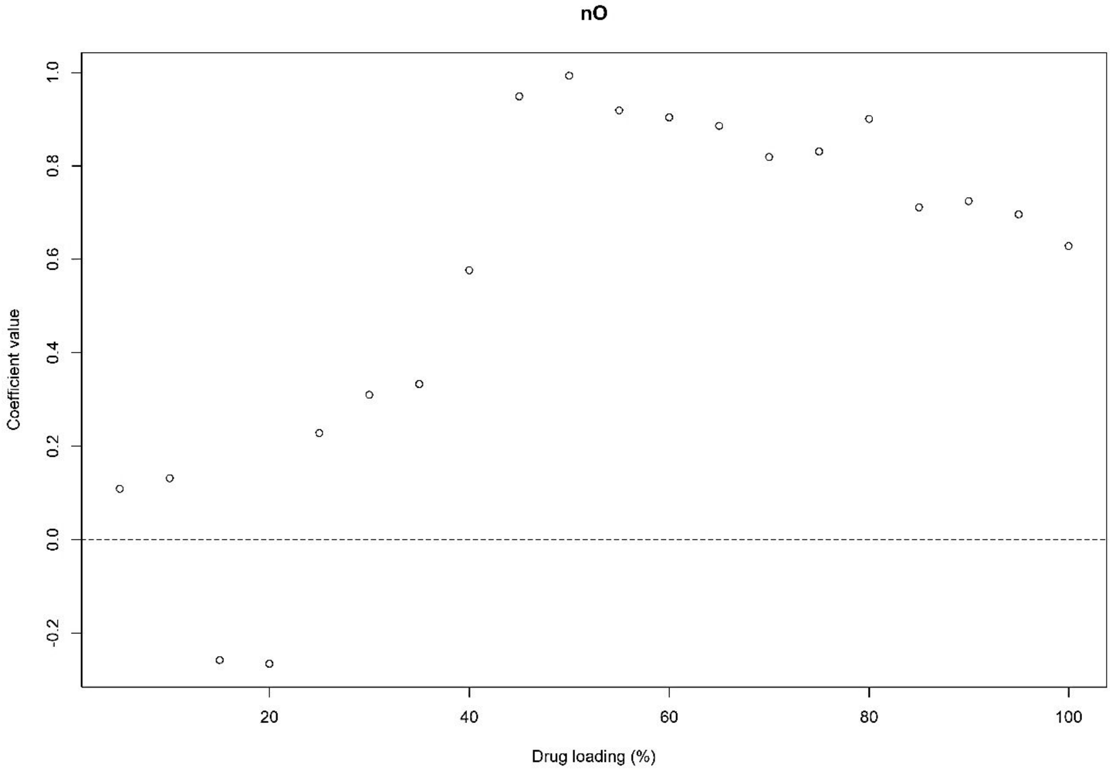

Figure S28. Scatter plot of coefficient values for the number of Oxygen atoms (nO) variable obtained from multiple linear regression analyses across 23 different drugs at varying drug/polymer loadings from 5 to 100%.

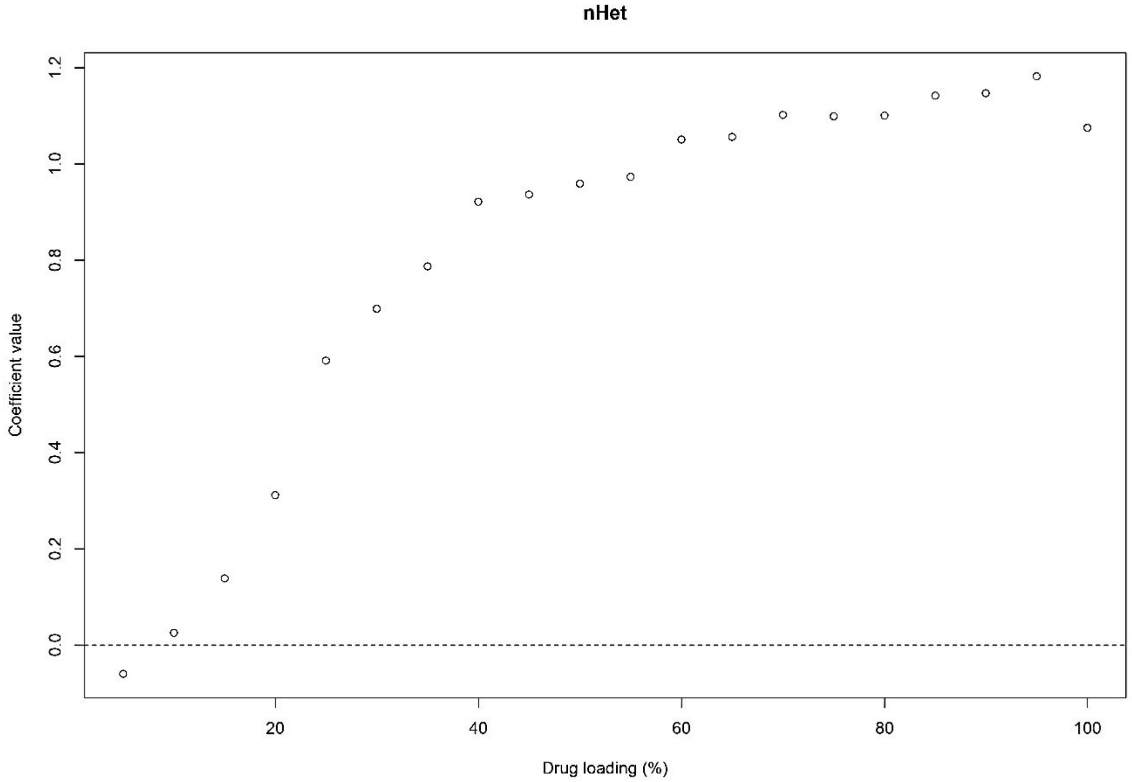

Figure S29. Scatter plot of coefficient values for the number of heteroatoms (nHet) variable obtained from multiple linear regression analyses across 23 different drugs at varying drug/polymer loadings from 5 to 100%.

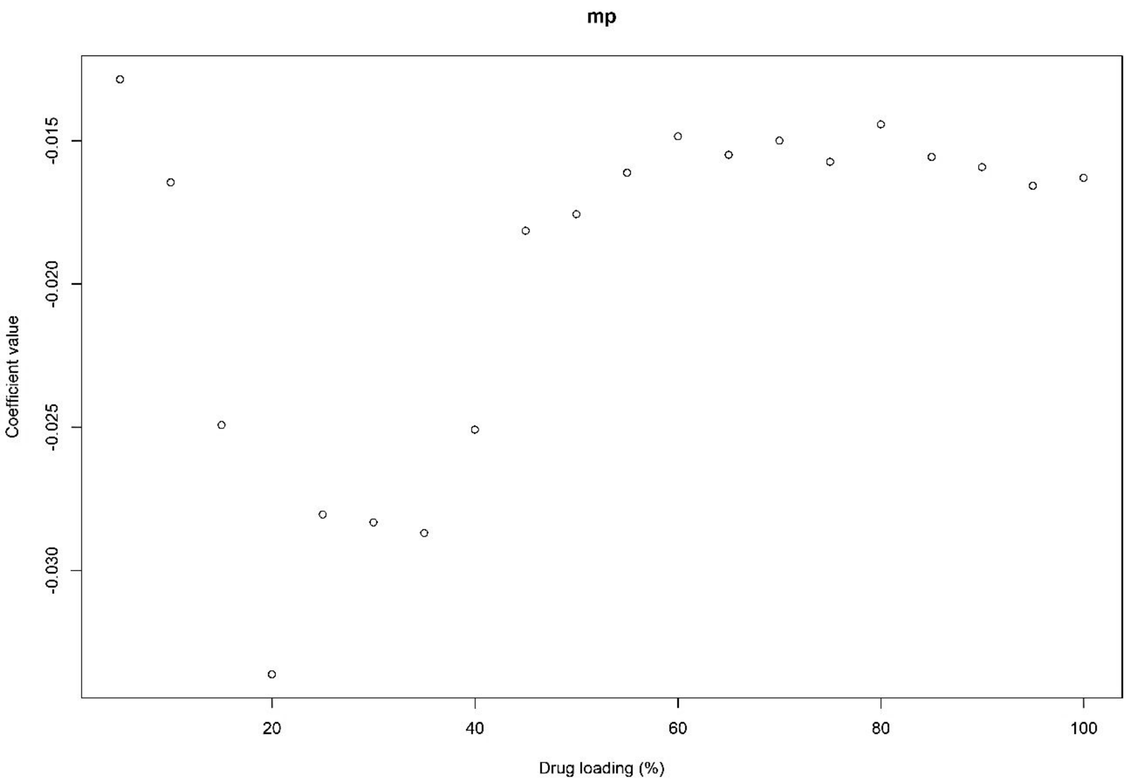

Figure S30. Scatter plot of coefficient values melting point (mp) variable obtained from multiple linear regression analyses across 23 different drugs at varying drug/polymer loadings from 5 to 100%.

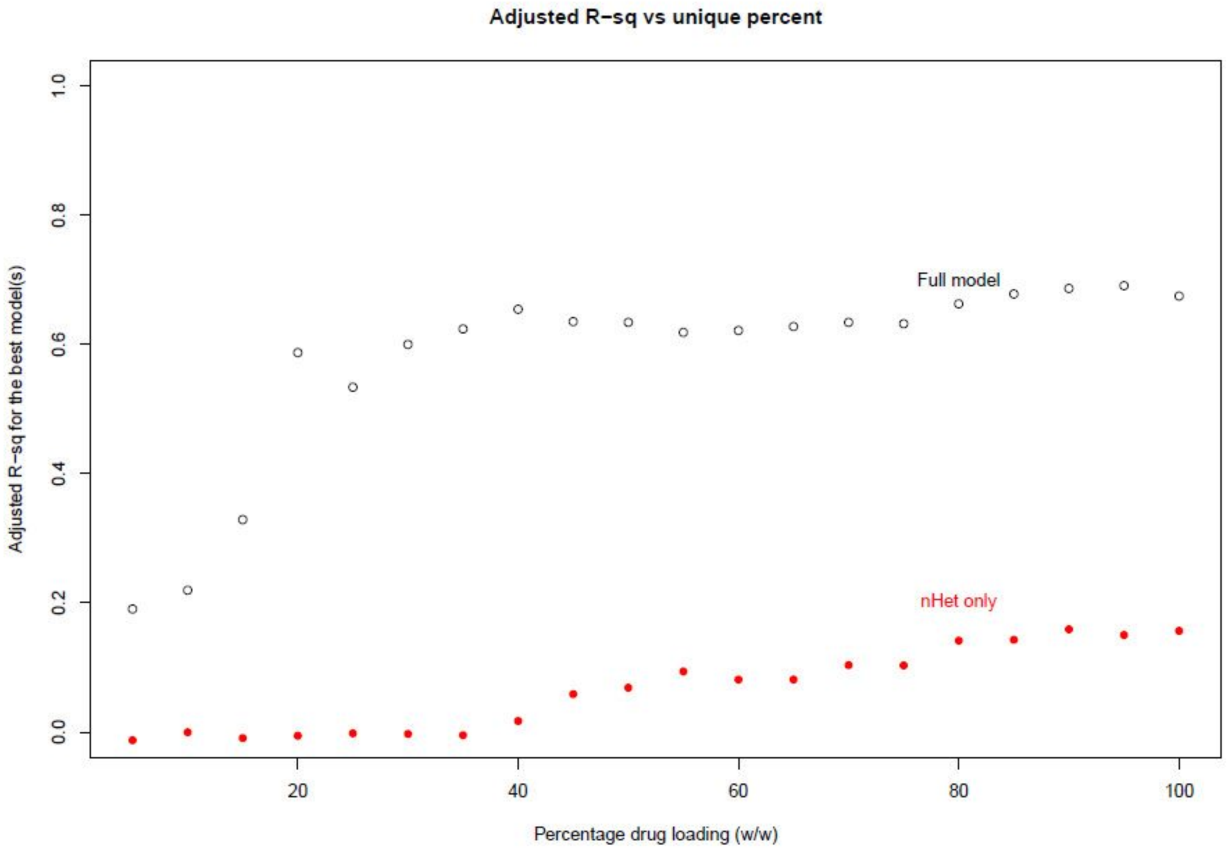

Figure S31. Fitted R2 extracted from the multiple linear regression model for the PVPVA data set, using all drug/polymer loadings (5-100% with 5% increment) for all the employed APIs. Comparing fitted/adjusted R2 Using a multivariable model (shown at the top in black) versus using a single-variable model based solely on nHet at the bottom in red.

To evaluate the impact of multicollinearity, a test using a single-variable model based solely on nHet was conducted. The results in this figure indicate that the single-variable model utilising nHet alone offers a significantly inadequate representation of our data, especially compared to the comprehensive multivariable model presented in this study. This highlights the necessity of employing a multivariable approach to achieve accurate predictive capabilities.

6. Comparison of predicted log stability, determined using the LOOCV method based on the multiple linear regression model, versus measured log stability from experimental microarray data across a range of drug loadings within the PVPVA polymeric matrix

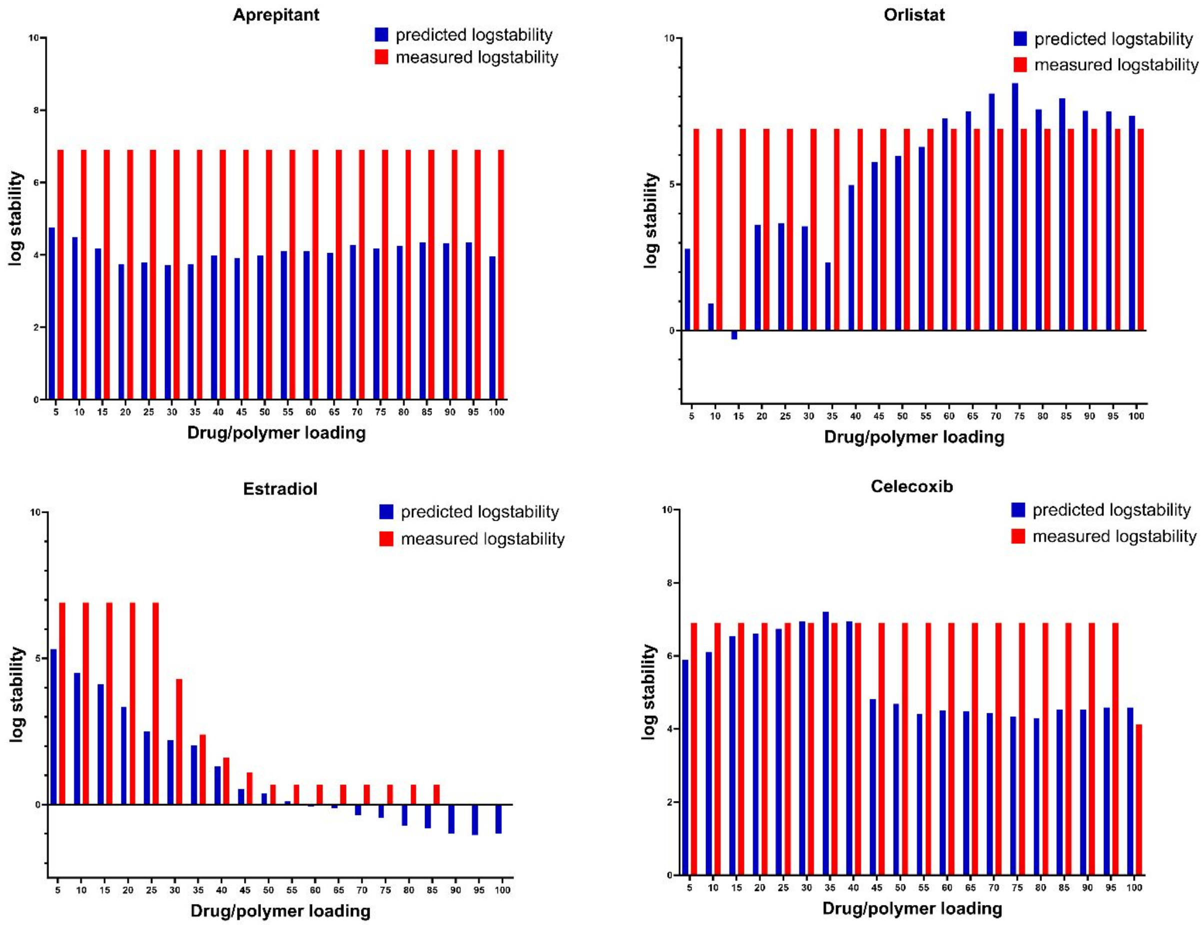

Figure S32. Comparison of predicted log stability, determined using the LOOCV method based on the multiple linear regression model, versus measured log stability from experimental microarray data for Aprepitant Orlistat, Estradiol, and Celecoxib across a range of drug loadings within the PVPVA polymeric matrix. The blue bars indicate the predicted stability, while the red bars show the measured stability, allowing for an evaluation of the predictive model's accuracy at different drug loadings.

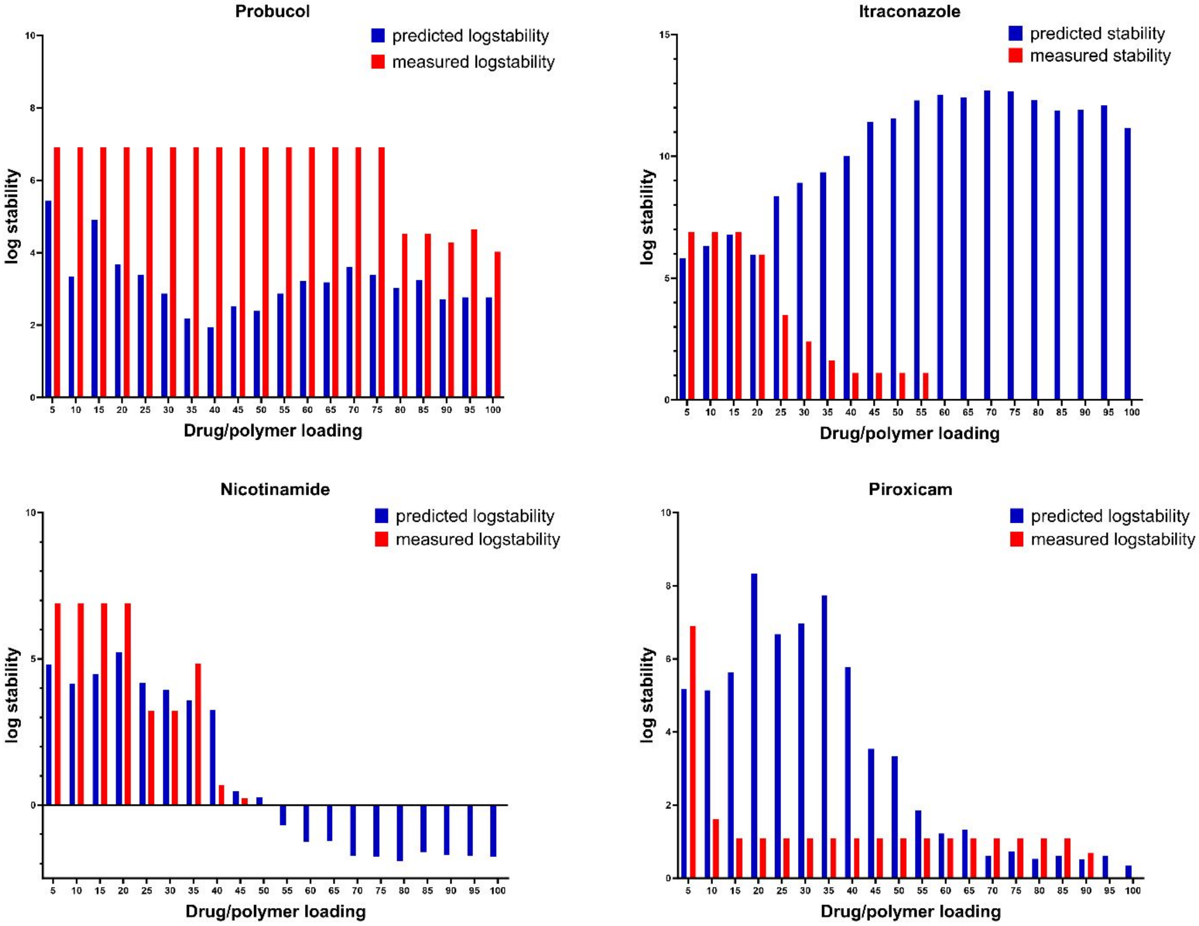

Figure S33. Comparison of predicted log stability, determined using the LOOCV method based on the multiple linear regression model, versus measured log stability from experimental microarray data for Probucol, Itraconazole, Nicotinamide, and Piroxicam across a range of drug loadings within the PVPVA polymeric matrix. The blue bars indicate the predicted stability, while the red bars show the measured stability, allowing for an evaluation of the predictive model's accuracy at different drug loadings.

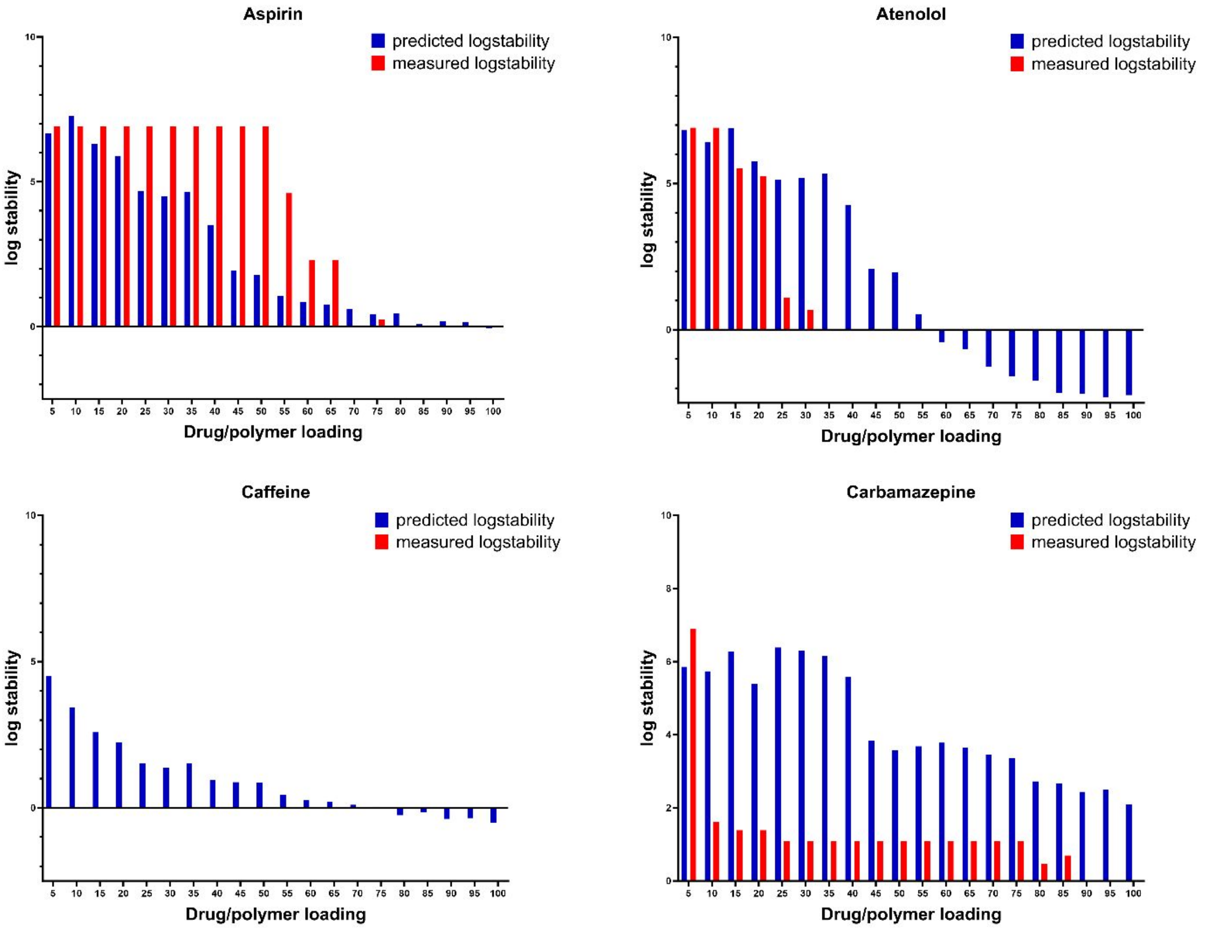

Figure S34. Comparison of predicted log stability, determined using the LOOCV method based on the multiple linear regression model, versus measured log stability from experimental microarray data for Aspirin, Atenolol, Caffeine, and Carbamazepine across a range of drug loadings within the PVPVA polymeric matrix. The blue bars indicate the predicted stability, while the red bars show the measured stability, allowing for an evaluation of the predictive model's accuracy at different drug loadings.

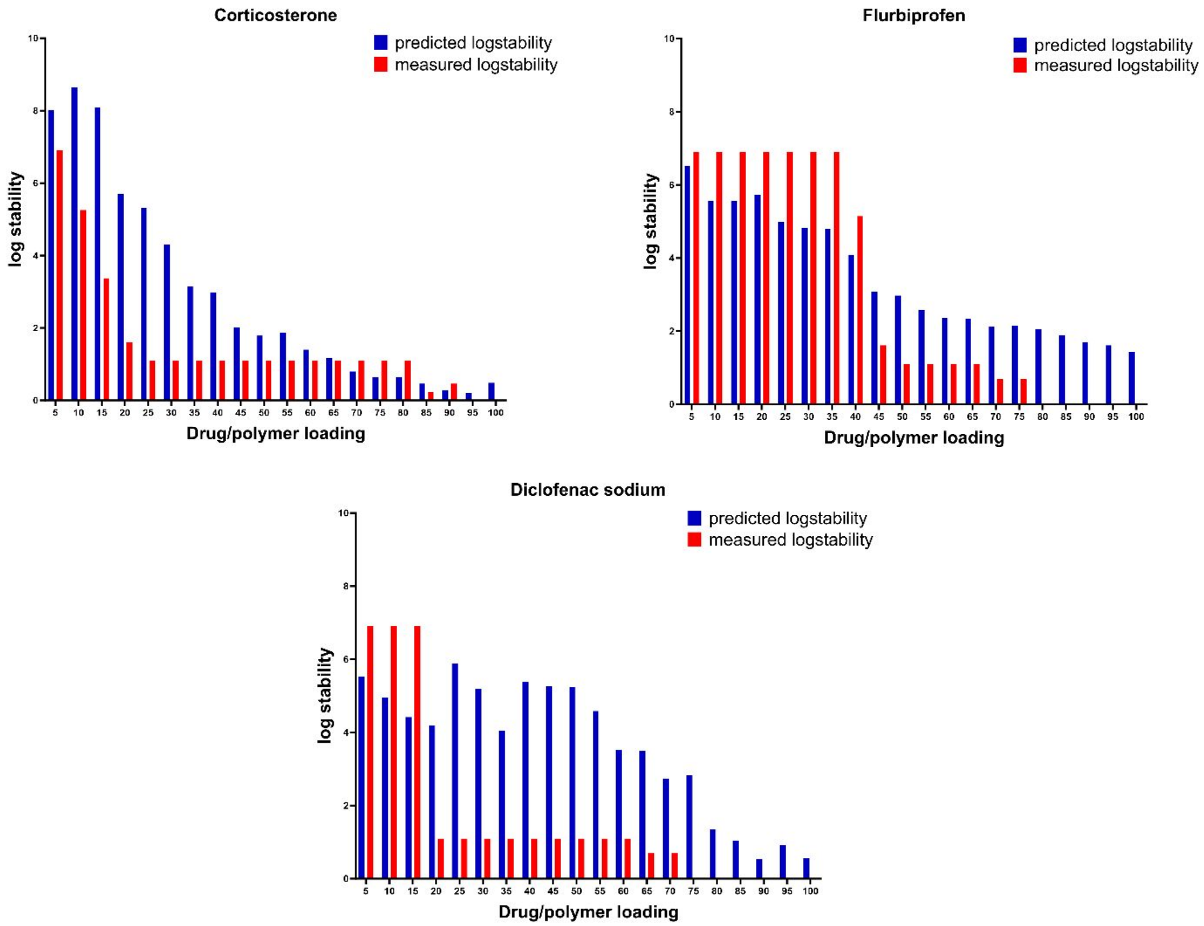

Figure S35. Comparison of predicted log stability, determined using the LOOCV method based on the multiple linear regression model, versus measured log stability from experimental microarray data for Corticosterone, Flurbiprofen and Diclofenac Sodium across a range of drug loadings within the PVPVA polymeric matrix. The blue bars indicate the predicted stability, while the red bars show the measured stability, allowing for an evaluation of the predictive model's accuracy at different drug loadings.
